# A systematic review of statistical models and outcomes of predicting fatal and serious injury crashes from driver crash and offense history data

**DOI:** 10.1186/s13643-020-01475-7

**Published:** 2020-09-28

**Authors:** Reneta Slikboer, Samuel D. Muir, S. S. M. Silva, Denny Meyer

**Affiliations:** Centre for Mental Health, Faculty of Health Arts and Design, Swinburne University of Technology, PO Box 218, Mail H31, John St, Hawthorn, Victoria 3122 Australia

**Keywords:** Systematic review, Quality assessment tool, Crash, Traffic, Offense, Statistics, Statistical modeling, Driver offenses, Crash history

## Abstract

**Background:**

Expenditure on driver-related behavioral interventions and road use policy is often justified by their impact on the frequency of fatal and serious injury crashes. Given the rarity of fatal and serious injury crashes, offense history, and crash history of drivers are sometimes used as an alternative measure of the impact of interventions and changes to policy. The primary purpose of this systematic review was to assess the rigor of statistical modeling used to predict fatal and serious crashes from offense history and crash history using a purpose-made quality assessment tool. A secondary purpose was to explore study outcomes.

**Methods:**

Only studies that used observational data and presented a statistical model of crash prediction from offense history or crash history were included. A quality assessment tool was developed for the systematic evaluation of statistical quality indicators across studies. The search was conducted in June 2019.

**Results:**

One thousand one hundred and five unique records were identified, 252 full texts were screened for inclusion, resulting in 20 studies being included in the review. The results indicate substantial and important limitations in the modeling methods used. Most studies demonstrated poor statistical rigor ranging from low to middle quality. There was a lack of confidence in published findings due to poor variable selection, poor adherence to statistical assumptions relating to multicollinearity, and lack of validation using new data.

**Conclusions:**

It was concluded that future research should consider machine learning to overcome correlations in the data, use rigorous vetting procedures to identify predictor variables, and validate statistical models using new data to improve utility and generalizability of models.

**Systematic review registration:**

PROSPERO CRD42019137081

## Background

Expenditure on driver-related behavioral interventions and road use policy is often justified by their impact on the frequency of fatal and serious injury (FSI) crashes. Fatal and serious injury includes death and injuries that endanger human life, including fetuses. Injury can be acute, cumulative, and protracted [1]. Due to the increased rarity of FSI crashes, because of factors such as improved vehicle design and road infrastructure, reliably evaluating the short- and medium-term impact of interventions on FSI crashes is challenging. On the other hand, traffic offenses (e.g., speeding and disobeying traffic lights) are much more frequent than FSI crashes [2, 3]. This has led to organizations using offending patterns as a proxy measure to evaluate the effectiveness of new interventions and policies targeting the reduction of FSI crashes.

Predictive models of FSI crashes can be complex and include variables from multiple domains. Environmental factors, road conditions, legal factors, licensing factors, and driver characteristics have all been found to contribute to FSI crash involvement [4–12]. Offense history (i.e., the number of traffic infringements a driver has incurred) and crash history (i.e., the number of crashes a driver has been involved in) have also frequently been found to be useful predictors of future FSI crashes [13–15]. Offense histories that include repeated violations over time, such as exceeding the speed limit and failure to obey road signs, have been found to increase crash risk. The increase in crash risk is particularly high when repeated violations lead to license suspension or revocation. Involvement in multiple crashes over time is a stronger predictor of future crash involvement than traffic violations [13, 14, 16]. Indeed, even particularly risky offenses do not appear to always increase the risk of FSI crashes. For example, Leal and Watson [17] reported that for those who engage in illegal street racing only 3.7% of offenses result in crashes, none of them being fatal.

A handful of reviews have investigated the modeling of FSI crashes [18–22]. A common critique in these reviews concerns the suitability of the traditional statistical techniques that have been applied to this data [22]. Considering the wide-ranging implications for developing and employing a statistical model to help inform decisions around policy and funding, it is vital that models are developed using rigorous and suitable methods, producing models that can be understood by non-technical audiences. Our review emphasizes the statistical approaches and methodologies applied to modelling FSI crash data. However, we were unable to find an existing quality assessment tool that met this need and have therefore developed our own.

Further, while statistical heterogeneity between primary studies is regularly noted as a limitation in systematic reviews and meta-analyses, detail such as the rigor and suitability of the modeling methods, as well as the ease of model interpretation is rarely discussed [9, 23–27]. In this review, we focus on an area of importance to policy makers. We focus exclusively on how offense history and crash history predict future FSI crashes and how such associations are best modeled. No previous reviews have had this focus and a new quality assessment tool has been developed specifically for this review because nothing suitable could be found.

### Aims

The overall research question was to assess the state of evidence for the prediction of FSI crashes from offense history and/or crash history by completing a systematic review of published literature and grey literature. Based on the failure to identify a systematic review focused on our research question and failure to identify a statistical quality assessment tool, two specific aims were formed. The first aim was to determine the type and quality of statistical analyses applied to the prediction of FSI crashes from driver offense and crash history. The quality of the papers included in the review was assessed based on the reporting of statistical assumptions, the reporting of statistical results, and the reporting of considerations specific to the statistical methods used. The second aim was to summarize the evidence and outcomes of studies that include offense history and crash history as predictors of FSI crashes.

## Methods

### Protocol and registration

The Preferred Reporting Items for Systematic Reviews and Meta-Analyses (PRISMA) were followed for the review [28]. The protocol was registered with the International Prospective Register of Systematic Reviews (PROSPERO), registration number: CRD42019137081.

### Inclusion and exclusion criteria

#### Individuals and outcome of interest

There were no restrictions on the type of individuals (i.e., drivers) included in the review. Efficacy and outcomes of interventions were not the subject of this review. Rather, we were interested in how the prediction of FSI road crashes has been modeled when described by driver offense and crash history. FSI crashes are the outcome of interest. A fatality is defined as a death that results from a crash, while serious injury resulting from a crash is defined as long-term impairment or loss of body function, permanent serious disfigurement, severe long-term mental, or severe long-term behavioral disturbance or disorder, or loss of a fetus [1]. Studies that examined crashes that did not result in fatalities or serious injury were not included in this review.

#### Data

Only models based on observational data were included. Data derived from laboratory tests, simulated data, self-report measures, and driving simulations were excluded. Studies that presented models built on qualitative data, mixed qualitative and quantitative data, and simulated data were excluded. Instantaneous traffic data used in real-time traffic crash prediction were excluded because these models typically apply only to a small section of the road.

#### Model specifications

To be included in the review studies must have presented a quantitative model predicting FSI crashes for individual drivers. That is, FSI crashes must be the outcome variable. A model was excluded from the review if the dependent variable included only minor crashes, such as when those involved in the accident received cuts and bruises and the vehicle had minor/repairable damage. When the dependent variable included a combination of serious and minor crashes, the model was excluded. Models had to describe crash risk based on driver offense and/or crash history. Models using longitudinal data were included. Models that considered only summarized country or state level offense and crash data, rather than individual driver data as the unit of interest, were excluded.

#### Study design

A study or report was included if it was a primary study that presented a quantitative model predicting FSI crashes. Study designs that were excluded included those which reported only qualitative data, reviews, meta-analyses, case studies, and any study that did not present a model for crash prediction.

#### Publication date

Studies and reports published prior to 1984 were excluded because data without a digital format was rare prior to this date, making statistical analysis difficult.

### Search strategy

Four academic databases were systematically searched: Australian Transport Index: ATRI database (via informat); Transportation Research International Documentation (ITRD) database; Scopus; and Web of Science. Search terms were chosen to identify papers that included statistical models, serious crashes, and variables related to the driver (Appendix S1). For example for Scopus we used the following search string: (predict* OR model) AND (“serious crash*” OR “serious accident” OR “serious collision” OR “fatal accident” OR “fatal crash” OR “fatal collision” OR “road deaths” OR “road fatal*” OR “traffic fatal*” OR “collision fatal*” OR “accident fatal*”) AND (driver).

Grey literature was also searched for government reports of crash prediction. The grey literature search was focused on, but not limited to, high-income countries that have low road traffic fatality rates, as indicated by the World Health Organization [29] (i.e., < 19.9 deaths per 100,000 population per annum). This was to increase the chance of identifying relevant reports in relation to the time spent searching. The search for grey literature utilized Google and Google Scholar to identify relevant international organizations, their websites, and information repositories. A total of 58 official government and statistic websites were searched from 38 countries (Appendix S2).

A forward and backward search was conducted on the articles that met the inclusion criteria. Forward searching involved identifying articles that cited the included study. Forward searching was conducted in Scopus. The backward search consisted of screening the references of the included studies (Appendix S3).

### Selection of studies

Initial search records were recorded in a Word document. Every article title and the first author from each article were systematically entered into the “find” function to identify and remove duplicates. The first author conducted all searches, eliminated duplicates, screened titles, and abstracts against the inclusion/exclusion criteria, and produced a list for full-text screening. If the full text was not available, the record was excluded from the review at the title/abstract screen stage. An auditing trail was created using an inclusion/exclusion checklist for the full-text screen in Excel, in which reasons for exclusion were recorded. Two authors screened studies at the full-text stage in Excel; discrepancies were resolved by a third author.

### Quality assessment tool

Statistical models were assessed in three areas using a purpose-made statistical quality assessment tool. Firstly, the degree to which assumptions were met and whether the method used in the study was appropriate. Secondly, the validation of the statistical model presented including whether the model had been validated using fresh data allowing an evaluation of generalizability. Thirdly, how adequately the authors reported the analysis procedures used and the results.

The quality assessment tool was based on the Statistical Analyses and Methods in the Published Literature (SAMPL) guidelines [30]. Items from the guidelines were rewritten as questions and additional items were added when required (Table 1). The tool was divided into three sub-sections. The “Reporting of statistical methods” sub-section included assessments of data quality (5 items), preliminary analyses (1 item), primary analyses (11 items), and supplemental analyses (4 items). The “Reporting of results” sub-section was made up of items assessing the reporting of numbers, descriptive statistics and averages (8 items), reporting risk ratios (5 items), and validation (2 items). The third section assessed “Method specific quality indicators” for regression analysis (10 items), survival analysis (13 items), and structural equation modeling (5 items). Studies were not assessed on items irrelevant to the methods used.

**Table 1:** Quality assessment tool

Sub-section and area of interest	Items
Section 1—Reporting of statistical analysis	
Data quality	Does the study report information about the research population? Does the study report what type of data has been used for the analysis, whether it is primary data (collected initially for the study) or secondary data (from a different source)? If the study used secondary data does the study name, the databases which have been used for the analysis? If the study has used different databases, does the study describe any linkage between the databases? Does the study report whether the data set is a representative data set?
Preliminary analyses	Does the study report any statistical procedures used to modify raw data before the analysis?
Primary analyses	Does the study describe about the purpose of the analysis? Does the study identify the variables used in the analysis and summarize each with descriptive statistics? Does the study describe the main methods fully, for analyzing the primary objectives of the study? Is the study clear on which method is used for each analysis, rather than just listing all statistical methods used in one place? If the method includes any assumptions, does the study verify that the data conformed to the assumptions of the test used to analyze them? Does the study indicate whether and how any allowance or adjustments were made for multiple comparisons (performing multiple hypothesis tests on the same data)? Does the study report how it deals with missing data? If relevant, does the study report how any outlying data were treated in the analysis? Does the study report the alpha level (e.g., 0.05) that defines statistical significance? Does the study report the name of the statistical package or program used in the analysis? If the study needs to control any variables for its objective, does the study report it properly?
Supplementary analyses	Does the study describe sensitivity analyses if applicable? Does the study test for the underlying assumptions of the methods used in the analysis? Does the study identify post hoc analyses, including unplanned subgroup analyses, as exploratory? If there is an imbalance that exists in the outcome variable of the data set, does the study report how the training phase overcome this issue?
Section 2—Reporting of results	
Reporting numbers and descriptive stat avg	Does the study report numbers—especially measurements—with an appropriate degree of precision. For ease of comprehension and simplicity, rounded to a reasonable extent? Does the study report total sample and group sizes for each analysis? Does the study report numerators and denominators for all percentages? Does the study summarize data that are approximately normally distributed with means and standard deviations (SD)? Use the form: mean (SD), not mean ± SD? Does the study summarize data that are not normally distributed with medians and interpercentile ranges, ranges, or both (report the upper and lower boundaries of interpercentile ranges and the minimum and maximum values of ranges, not just the size of *t*)? Does the study report the variability of the data set using either standard deviations, inter-percentile ranges, or ranges (the SE is an inferential statistic—it is about a 68% confidence interval—not a descriptive statistic)? Does the study display summarized or exact data in tables? Does the study display data in figures? Tables present exact values, and figures provide an overall assessment of the data?
Reporting risk and ratios	Does the study describe the type of rate (e.g., incidence rates; survival rates), ratio (e.g., odds ratios; hazard ratios), or risk (e.g., absolute risks; relative risk differences), being reported? Does the study describe the quantities represented in the numerator and denominator? Does the study report the time period over with each rate applies? Does the study report any unit of population (that is, the unit multiplier: e.g., × 100; × 10,000) associated with the rate? Does the study consider reporting a measure of precision (a confidence interval) for estimated risks, rates, and ratios?
Validation	Does the study describe methods of validation used in the training phase (e.g., cross validation, use of test/hold-out sample)? Does the study describe the attempts to generalize the model beyond the immediate context?
Section 3—Method specific quality indicators	
Regression analysis	Does the study describe the purpose of the analysis? Does the study confirm that the assumptions of the analysis were met? For example, in linear regression indicate whether an analysis of residuals confirmed the assumptions of linearity. Does the study report the regression equation for either simple or multiple (multivariable) regression analyses? For primary comparisons analyzed with simple linear regression analysis, does the study consider reporting the results graphically, in a scatter plot showing the regression line and its confidence bounds? Does the study report the alpha level used in the univariate analysis? Does the study report whether the variables were assessed for collinearity? Does the study report whether variables were assessed for interactions? Does the study describe the variable selection process by which the final model was developed (e.g., forward stepwise; best subset). Does the study report the regression coefficients (beta weights) of each explanatory variable and the associated confidence intervals and *P* values, preferably in a table? Does the study provide a measure of the model’s “goodness-of-fit” to the data (the coefficient of determination, r2, for simple regression and the coefficient of multiple determination, R2, for multiple regression)?
Survival analysis	Does the study describe the purpose of the analysis? Does the study describe the dates or events that mark the beginning and the end of the time period analyzed? Does the study specify the circumstances under which data were censored? Does the study specify the statistical methods used to estimate the survival rate? Does the study confirm that the assumptions of survival analysis were met? For each group, give the estimated survival probability at appropriate follow-up times, with confidence intervals, and the number of participants at risk for death at each time. It is often more helpful to plot the cumulative probability of not surviving. For each group, give the estimated survival probability at appropriate follow-up times, with confidence intervals, and the number of participants at risk for death at each time. It is often more helpful to plot the cumulative probability of not surviving. Reporting median survival times, with confidence intervals, is often useful to allow the results to be compared with those of other studies? Does the study present the full results in a graph (e.g., a Kaplan-Meier plot) or table? Does the study specify the statistical methods used to compare two or more survival curves? Does the study report the *P* value, when comparing two or more survival curves with hypothesis tests? Does the study report the regression model used to assess the associations between the explanatory variables and survival or time-to-event? Does the study report a measure of risk (e.g., a hazard ratio) for each explanatory variable, with a confidence interval?
SEM models	Does the study report all the parameters and their standard errors? Does the study report the reason for the choice of a clear and complete form of path model structure? Does the study report the global indices of fit? Does the study provide reasons as justification for omitted directed and non-directed arcs? Does the study report alternative and equivalent models?

### Quality assessment procedure

Included studies were quality assessed by two independent authors (SM and SS) using the purpose-made quality assessment tool. The quality assessment tool was completed as a Google doc and automatically exported into an Excel file for analysis. Disagreements were resolved by a third author (DM). Items were allocated a score of 1 for yes, 0 for no, or NA for not applicable with higher quality papers receiving higher quality scores. Sub-section quality scores were calculated by averaging responses to reveal a quality score ranging from 1 when all quality indicators were met, to 0 when no quality indicators were met. When an item was not applicable, that item was excluded from the mean score of that study’s rating. Then each reviewers’ sub-section quality scores were averaged to get a final quality score for every study independently. Finally, the independently reviewed quality scores of the two reviewers were averaged to get an overall quality measure for each study. Studies were then categorized as scoring low (0 to 0.333), medium (0.334 to 0.666), and high (0.667 to 1.00); i.e., 1/3 = 0.333 with one being the totality of all studies included in the review.

## Results

**Fig. 1:**
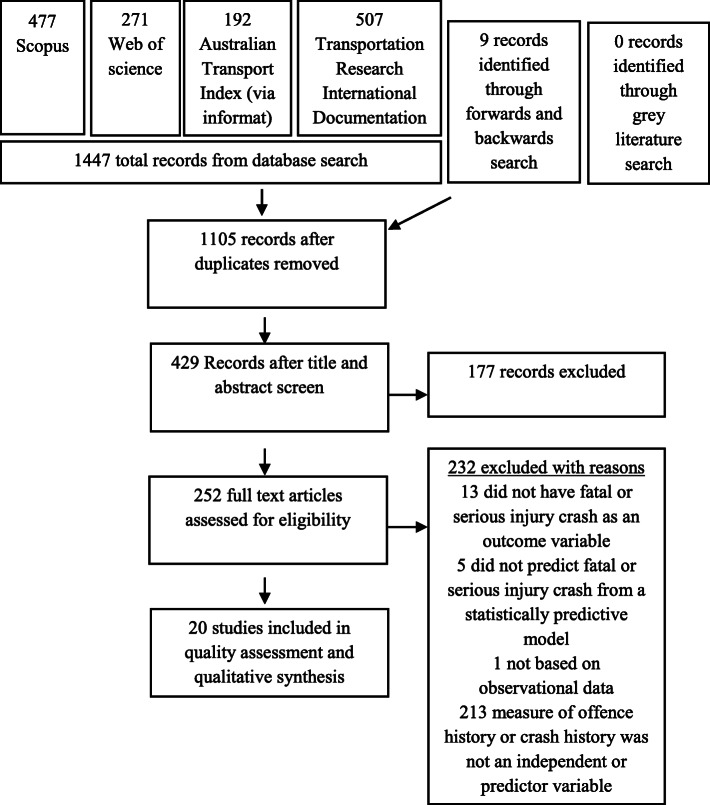
PRISMA flowchart

The flow of studies from identification, through screening for eligible inclusion, to the final synthesis, is presented in the PRISMA flowchart, Fig. 1. One thousand one hundred and five records were identified. Of the 252 studies included in the full-text screen (Appendix S4), 243 were identified in the database search and nine were identified in the forwards and backward search. Agreement between the two reviewers (RS and SM) of the full texts for inclusion was low (*k* = 0.469, *p* < 0.00). These disagreements were resolved by a third author (SM), resulting in twenty studies being included in the quality assessment, comprising of data from a total of 2,379,862 individuals. The statistical techniques, findings, and characteristics of the included studies are presented in Table 2.

**Table 2:** Details of study characteristics

Study, country, vehicle type, quality	Design, data type, number of drivers/crashes	Independent predictor	Confounders controlled	Dependent variable	Statistical technique used, measure of risk	Main finding of interest
Lui and Marchbanks [31], USA, cars, high	Longitudinal, FARS (1984‑1986), population, drivers that have had a speeding conviction (*n* = 46,266)	Date of prior crash, suspension or conviction	None	Date of fatal crash	Survival analysis	Involvement in a fatal crash will occur by 5 years post the prior crash, suspension, or conviction.
Perneger and Smith [32], USA, cars and light pick ups, mid	Matched-pairs design, FARS (1986), population, two-car crashes (*n* = 6506)	Invalid license, prior DWI, prior suspension, prior speeding, prior crash within 12 months, 13‑24 and 25‑36 months ago	Environment, exposure to traffic, differences in case fatality	Culpable or non-culpable drivers	Logistic regression, odds ratio	Invalid driving license, prior DWI, and prior license suspension increases the likelihood of initiating a fatal crash.
Rajalin [33], Finland, truck, bus, and drivers over 65 years not included, low	Case-control design, (Study 1) VALT and traffic offense register, sample, drivers involved in fatal crash (*n* = 615), random selection of license holders (*n* = 776)	Offense rate	Distance driven	Drivers in fatal crashes or control	Logistic regression, odds ratio	Drivers involved in a fatal crash have an offense rate 1.51 times higher than control drivers.
Cooper [34], Canada, all vehicles, mid	Case-control design, police crash data and driver records in British Columbia (1991‑1994), population, with speeding conviction (*n* = 877,758), with other conviction (*n* = 154,103)	Thirteen types of prior traffic convictions (e.g., speeding, not obeying signal, failure to yield)	None	Rate of crashes per driver	Logistic regression, estimates	All conviction types contribute to increasing the chance of being in a subsequent FSI crash, convictions associated with speeding and DWI were the most important predictors.
Wundersitz et al. [35], Australia, all vehicles, low	Matched-pairs design (drivers paired from same fatal crash group), TARS (1999 to 2002), South Australian population, Culpable drivers (*n* = 182), non-culpable drivers (*n* = 206)	Four offense types and grouped offenses over 5 years prior to the fatal crash, crashes and culpable crashes over 5 years prior to fatal crash	None	Culpable vs non-culpable involvement in fatal crash	Not reported, odds ratio	Not significant
Kim et al. [36], USA, all vehicles, mid	Case-control design, NPTS and FARS (1995‑1997) population, *n* not reported	Previous traffic offense yes/no	None	Probability of survival, probability of crash	Logistic regression, odds ratio	If a previous traffic offense is present it decreases the likelihood of surviving a one or two-vehicle crash but increases the likelihood of surviving a multi-vehicle crash. A previous traffic offense increases the likelihood of having a crash for one, two, and multi-vehicle crashes.
Blower and Green [37], USA, buses, mid	Case-control design, BIFA from FARS (1999‑2005), population, drivers (*n* = 2102)	Previous violation or not in previous 3 years, previous crashes or not in previous 3 years	Type of bus service: school, intercity, charter/tour, other	Driver error in fatal crash or not	Logistic regression, odds ratio	Previous violations and crashes each increase the chance of the driver making an error by 1.3 times. Drivers with a violation on their record are 27% more likely to commit an error in a fatal crash than those without a violation.
Malchose and Vachal [38], USA, all vehicles, mid	Case-control design (involved in FSI crash or not), NDDL, (2006 to 2009), North Dakota population, crashes (*n* = 317), teen drivers aged between 14 to 17 years (*n* = 20,392)	No-risk convictions (e.g., parking), risk convictions (e.g., speeding), previous property damage only crash, in the first year or prior to first fatal or injury crash, previous at-fault property damage only crash, in the first year or prior to first fatal	Gender, age risk convictions, rural/urban	Involvement in a fatal or injury crash in the first year of licensure or not	Logistic regression, log odds	Drivers with previous convictions are 0.5 times; and drivers with previous property damage only crash histories are 25.5 times likely to be involved in a fatal or injury crash.
Lueck and Murry [39], USA, truck involved crashes, low	Case-control design, MCMIS and CDLIS, population, crashes (*n* = 30,090), drivers in a crash or roadside inspection (*n* = 587,772)	The ability of those with and without a violation/conviction and crash history (2008)	None	Crashes (2009)	Not reported, reported percentages	Twenty-three of the 34 independent variables significantly increased the chance of being in a crash, increases ranged from 18% to 96% Any previous conviction increased the chance of being in a crash by 56% Prior crash increased the chance of being in a future crash by 88%
Gates et al. [40], USA, heavy trucks, > 26,000 lbs, mid	Case-control design, FARS (1993‑2008), population, driver > 20 years old involved in a fatal crash (*n* = 65,867)	Stimulants present or absent	Past 3 years, prior crashes, DWI, speeding infractions, other infractions, suspensions	Responsibility for crash measured by UDA preceding the crash	Logistic regression, reported percentages	Odds of an UDA increased by: 30% one or more prior crash 24% other moving conviction 26% one prior suspension 14% previous speeding conviction
Factor [41], Israel, all vehicles, mid	Case-control design, ICBS (2002‑2008), 20% representative sample, drivers (*n* = 409,051)	Traffic tickets per year (over 13 years)	Daily distance traveled, gender, age, religion, education, social class, vehicle type	Driver involve in FSI crash or not	Logistic regression, odds ratio	As traffic tickets increase so too do the odds of being in an FSI crash.
Reguly et al. [42], USA, heavy trucks, > 26,000 lbs, mid	Case-control design, FARS (1993‑2008), population, negative drug test (*n* = 8325), positive drug test (*n* = 102)	Opioid analgesic (drivers positive or negative drug test)	Collisions, DWI convictions, other convictions, speeding, license suspensions	Responsibility for crash measured by UDA preceding the crash	Logistic regression, estimate result, odds ratio	Odds of an unsafe driver action increased if the driver had a previous crash, a driving infraction or speeding infraction in the past 3 years.
Dubois et al. [43], USA; passenger vehicles, sport-utility vehicles, and light trucks; high	Case-control design, FARS (1991‑2008), population, driver > 20 years old involved in a fatal crash (*n* = 150,010)	BAC, cannabis, BAC and cannabis (drivers positive or negative drug test)	History of one, two, or three or more of crashes, DWI, speeding, suspensions	Responsibility for crash measured by an UDA preceding the crash	Logistic regression, odds ratio	Odds of an UDA increased by: 13% one prior crash 39% three or more prior crashes 26% one prior suspension 33% three or more prior suspensions
Kumfer et al. [44], USA, all vehicles, high	Case-control design, FARS (2010‑2012) California, Michigan, New York, North Carolina, Texas, and Washington, representative sample of USA, driver in a single-vehicle crash (*n* = 5110), driver in a multivehicle vehicle crash (*n* = 9986) (passengers not included)	History of suspensions or revocations (none, one, other), year of last crash or license suspension (no record, two since 2005, other)	None	Multivehicle or single vehicle	Logistic regression, odds ratio	Not significant
Feng et al. [45], USA, bus involved crashes, mid	Case-control design, BIFA from FARS (2006 to 2010) population, drivers (*n* = 1380)	Cluster 1 “middle age drivers with history of driving violations” Cluster 2 “young and elderly drivers with history of driving violations” Cluster 3 “drivers without history of driving violations” Valid license or not	None	Level 1 crashes < = 2 fatalities Level 2 crashes > 2, < 3 fatalities Level 3 crashes > = 3 fatalities	Logistic regression, estimate result and odds ratio	Cluster 1, low chance of being involved in level 2 and 3 crashes; cluster 2, high chance of being in level 1 and 2 crashes; cluster 3, ns
Li et al. [46], USA, all vehicles except heavy trucks, mid	Matched-pairs design (drivers paired from same fatal two-vehicle crash), FARS (1993 to 2014), population, culpable driver (*n* = 14,742), non-culpable driver (*n* = 14,742)	Concurrent alcohol and marijuana use	Previous 3-year crash history, previous 3-year DWI conviction, previous 3-year speeding conviction	Culpability or non-culpability in a fatal crash	Logistic regression, odds ratio	Having a crash history, a DWI conviction, a speeding conviction, and license suspension in the previous 3 years increases the likelihood of culpability in a fatal crash.
Hamzeie et al. [47], USA, all vehicles, mid	Case control design, FARS fatal crashes (2010 to 2014), population, drivers with known injury severity (*n* = 74,632)	Cannabis use, five levels of injury severity*	Number of previous license suspensions, number of previous speeding violations	Cannabis use yes/no	Logistic regression, odds ratio	Those with speeding violations and those who have had their license suspended are associated with higher levels of injury in fatal crashes.
Stringer [48], USA, all vehicles, high	Longitudinal, FARS and GSS (1993 to 2015), population, drivers (*n* = 2326)	Normative behavior, values and beliefs, local attitudes	Non-DUI fatal crashes, repeat DUI offender crashes	Total frequency of DUI fatal crashes in each county	Poisson multi-level growth curve	Non-DUI fatal crashes and repeat DUI offender crashes significantly predict future DUI fatal crashes
Mashhadi et al. [49], USA, trucks, mid	Case-control design, CARE and WCRVD (2011‑2014), Wyoming data from 3 interstate highways, single truck crash at fault (*n* = 1654), multi-vehicle crash truck not at fault (*n* = 696), multi-vehicle crash truck at fault (*n* = 847)	Violation record	None	Fatality/injury level, being in a single truck crash	Logistic regression	Not significant
Yuan et al. [50], USA, truck involved crashes, mid	Within group (involved in FSI crashes), TIFA and FARS (2010), population, crashes (*n* = 1555), drivers/occupants n not reported	Driver factors (latent factor) made up of five observed measures; belt use, driving experience, history of conviction, history of crash, gender, valid license or not	Other latent variables	Truck occupant injury factors (latent), accident size (latent)	Structural equation modeling, standardized regression weights	Crash history (measured) has a large effect on driver factors (latent) which decreases accident size (latent). Prior suspension, speeding, and convictions (measured) make a fatal accident more severe.

*Country* the country from which the data was collected; *TIFA database* trucks involved in fatal accidents database, *FARS* fatality analysis reporting system, *BIFA* buses involved in fatal accidents database, * the KABCO scale, *ns* not significant; *MCMIS* motor carrier management information system database, *CDLIS* commercial driver’s license information system, *NDDL* North Dakota Drivers’ License data; *TARS* Traffic Accident Reporting System, *NPTS* Nationwide Personal Transportation Survey, *VALT* Traffic Safety Committee of Insurance Companies, *GSS* General Social Survey from National Opinion Research Center, *ICBS* Israel Central Bureau of Statistics, *CARE* Critical Analysis Reporting Environment, *WCRVD* Wyoming court reported violation database, *DUI* driving under the influence, *DWI* driving while intoxicated, *FSI* fatal or serious injury crash, *UDA* unsafe driving action (used as a proxy measure)

### Vehicle type and population

Nine out of the 20 reviewed studies included all vehicle types (i.e., cars, motorcyclists, and trucks). The remaining 11 studies explored the prediction of FSI crashes from offense history and crash history using data related to distinct vehicle types: buses (1 study), heavy trucks (5 studies), and cars and light trucks (4 studies). Seventeen studies used data representing the population, three countries were represented—USA, Finland, and Canada, and two states were represented—North Dakota (USA) and South Australia (Australia). Of the studies using sample data, one used a 20% representative sample based on Israeli census data, one used data from six states in the USA, and a third study selected various highways within the USA state of Wyoming (Appendix S5).

### Quality assessment

**Fig 2:**
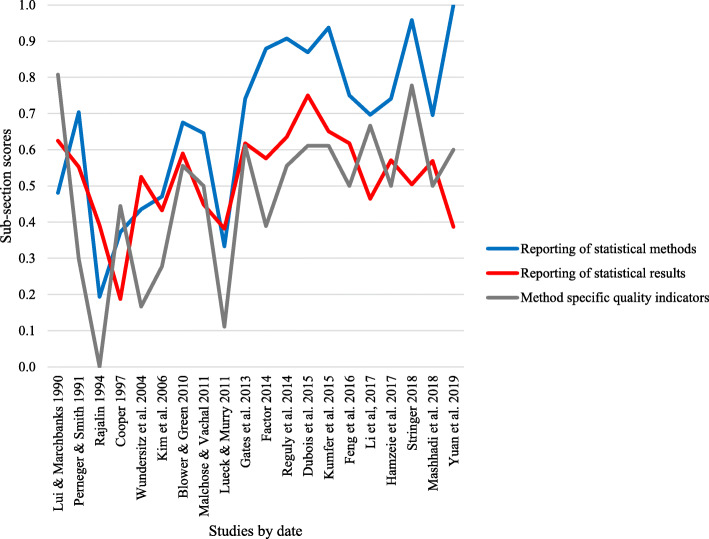
Quality assessment sub-section scores for included studies

Overall, there was high agreement between the two reviewers of statistical quality (inter-class correlation coefficient 0.919; Appendix S6). Fifteen studies used logistic regression and most of these studies received quality scores in the middle range (low = 3, middle = 12, high = 2). The only study to use structural equation modeling also received a quality score in the middle range. One study used a survival analysis, and one used a Poisson multi-level growth curve model, both receiving a high-quality score. Quality scores tended to be consistently higher for more recently published studies (Table 3). The improvement in quality seems to be led predominantly by improved “Reporting of statistical methods,” while the method specific quality indicators provide the poorest results (Fig. 2).

**Table 3:** Quality tool assessment results

Studies grouped by statistical technique	Reporting of statistical methods	Reporting of statistical results	Method specific quality indicators	Overall quality score
*Logistic regression*				
Perneger and Smith 1991	0.704	0.552	0.299	0.463
Rajalin 1994	0.193	0.391	0.000	0.146
Cooper 1997	0.372	0.188	0.444	0.362
Wundersitz et al. 2004	0.435	0.525	0.167	0.323
Kim et al. 2006	0.470	0.432	0.278	0.365
Blower and Green 2010	0.675	0.590	0.556	0.594
Malchose and Vachal 2011	0.646	0.449	0.500	0.524
Lueck and Murry 2011	0.333	0.382	0.111	0.234
Gates et al. 2013	0.741	0.617	0.611	0.645
Factor 2014	0.880	0.576	0.389	0.558
Reguly et al. 2014	0.907	0.636	0.556	0.664
Dubois et al. 2015	0.869	0.750	0.611	0.710
Kumfer et al. 2015	0.938	0.651	0.611	0.703
Feng et al. 2016	0.750	0.617	0.500	0.592
Li et al. 2017	0.697	0.464	0.997	0.755
Hamzeie et al. 2017	0.741	0.571	0.500	0.578
Mashhadi et al. 2018	0.696	0.569	0.500	0.566
*Structural equation modeling*				
Yuan et al. 2019	1.000	0.387	0.600	0.647
*Survival analysis*				
Lui and Marchbanks 1990	0.481	0.625	0.808	0.680
*Poisson multi-level growth curve*				
Stringer 2018	0.958	0.504	0.778	0.755

### Qualitative synthesis of study outcomes

Using binary logistic regression analyses, seven studies reported that offense history predicts FSI crash involvement. Perneger and Smith [32] found that having an invalid driving license or license suspension, or a prior DWI (driving while intoxicated) conviction increases the likelihood of initiating a fatal crash. Cooper [34] found that fourteen conviction types increase the chance of being in a subsequent FSI crash; speeding and DWI convictions were the most important. These findings are supported by Factor [41], who used traffic infringement tickets as a predictor variable, and Rajalin [33], who compared drivers who had had an FSI crash to those who had not. Further nuance to the role of offense history is added by Kim et al. [36]. Offense history increased the likelihood of having a crash regardless of the number of vehicles involved. Additionally, those with previous traffic offenses were less likely to survive a one or two-vehicle crash, but more likely to survive a crash involving three or more vehicles. Speeding violations and a license suspension, rather than any other type of offense, were associated with increased injury severity [47].

Six studies using binary logistic regression reported that both crash history and offense history increase the likelihood of FSI crash involvement. The odds of a driver making an error directly leading to an FSI crash increased by approximately 27% if they had been responsible for a previous crash, a driving infraction/violation, or speeding infraction [37, 38, 42]. Specifically, driver error leading to an FSI crash increased by 13% if the driver had one prior crash [43], 30% if the driver had one or more prior crashes [40], and 39% if the driver had three or more prior crashes [43]. Prior license suspension was a strong predictor of driver error leading to an FSI crash: one suspension increased the odds by 26% [40, 43] and three or more prior suspensions increased the odds by 33% [43]. Not only are offense history and crash history associated with driver errors causing an FSI crash, but they are also associated with the likelihood of the driver being legally culpable. Predictors of being legally culpable include a crash history, a DWI conviction, a speeding conviction, and a license suspension in the 3 years prior to the crash [46]. Property damage only crashes also predicted FSI crash involvement [38]. Feng et al. [45] took a unique approach by grouping drivers on several characteristics. Middle-aged drivers with a history of convictions had a low chance of being involved in a crash involving more than two fatalities. Young and elderly drivers with a history of violations had a high chance of being involved in a crash involving less than 3 fatalities [45].

Analyses other than logistic regression produced comparable findings. Based on longitudinal data, Stringer [48] found that crash history and offense history significantly predict future fatal crashes with crash history being the more important predictor. Yuan et al. [2019] found that prior suspension and speeding convictions are associated with fatal accidents. Finally, Lui and Marchbanks [31] found that should a fatal crash occur after license suspension, a conviction, or prior crash, it is likely to occur within 5 years. While the predictors of FSI crashes have been identified, these findings cannot be verified given that most of these studies were assessed as having only low to middle quality in our quality assessment.

## Critical discussion

### Strengths of logistic regression in this context

There are several reasons for the popularity of logistic regression. Logistic regression has many different forms allowing its use for conditional logistic regression with matched-pairs data [32, 45]; ordinal and nominal logistic regression when the dependent variable has several categories [45, 47]; direct logistical regression when no predictor variables are considered more or less important than the others [37]; sequential logistic regression when confounding variables need to be controlled [41, 43]; stepwise logistic regression when an exploratory approach is needed [49]; and censored regression when data for the dependent variable is incomplete [36]. Further, the assumptions for logistic regression are lenient. Also, logistic regression can perform several functions. It can predict group membership, identify important predictors, identify interactions among predictors and provide odds ratios for quantifying the effects of predictors. Moreover, the accuracy of logistic regression models is easily assessed using a variety of measures including the proportion of variance in the dependent variable explained by the predictors, using pseudo *R* squared values.

### Weaknesses of the use of logistic regression in this review

Most of the papers that applied a logistic regression model in this review was rated as “medium” quality. There were striking statistical deficiencies found in these studies. Firstly, the selection of predictor variables and the number of predictors included often appeared to be made post hoc, i.e., inclusion was justified only after the model was created. Given the scope of variables available from large population databases recording fatal crashes, a surprising observation was that only five studies [32, 37, 40, 43, 49] described the variable selection process used. While the practice of post hoc justification of predictors is common, it is damaging to the integrity of findings and real-world implications. Logistic regression is often used to inform life and death decisions; therefore, inadequate or poor a priori variable selection may indeed lead to unwanted consequences.

Secondly, of vital importance to the quality of analyses and subsequent confidence in findings is the adherence to underlying statistical assumptions. Only two studies using logistic regression confirmed that the assumptions were met [46, 47]. Particularly problematic within the studies using logistic regression was the failure to check for correlations between predictor variables (multicollinearity) and interactions. Of studies using logistic regression, only two studies reported checking multicollinearity [44, 46], and only five studies, checked for interactions [40, 42–44, 46]. The presence of multicollinearity and interactions within the predictor variables artificially increases their ability to predict FSI crashes and makes interpretation of odds ratios problematic. Not meeting these assumptions raises questions about the validity of the results.

A critical observation repeatedly made by reviewers of statistical models is the inclusion of unwanted correlations in data. Examples are, serial correlation, i.e., correlation over time [18], spatial and temporal correlation between predictor variables, and correlation between predictor variables and error [20]. The inclusion of these correlations in data results in incorrect inferences being made from the results. To illustrate the problem, helmet use may appear to reduce crash fatalities, yet when a biological or psychological predisposition to risk predicts both helmet use and crash involvement, helmet use may no longer be a significant predictor of fatalities. This argument is supported by a clear relationship between sensation seeking and risky driving [51]. The problem of unwanted correlation in the data is relevant in this review, when contemporaneous offense and crash data are considered. It is important to note that there is likely to be a two-way relationship between offense history and crash history in this context. Not only is someone with an extensive offense history more likely to have a crash. In addition, it is likely that a crash caused by a driver will lead to the driver being charged with an offense [49].

The third weakness identified in the reviewed studies using logistic regression is the lack of validation. For example, none of the studies reported the results of a Hosmer Lemeshow goodness of fit test or areas under the receiver operating characteristic curve. Validation of final models using new data and the use of validation during model creation was not conducted anywhere in the reviewed literature. Given the pervasive international interest and enormous volume of publications on FSI crashes, the lack of validated models that predict FSI crashes from offense history, crash history, and licensing variables is an important limitation. However, one of the papers in this review [46] did conduct two sensitivity analyses for their models. One of these sensitivity analyses tested their model with data from the USA states that accounted for more than 80% of FSI crashes in the USA and the second sensitivity analysis tested the model on data for two different time periods.

The fourth major limitation is lack of effective calibration of the models, considering any imbalance in the data in order to provide accurate estimates of classification accuracy. Indeed, no study in this review presented a confusion/classification matrix to evaluate how well the model classified participants into the correct categories. Both validation and calibration must be conducted after a model has been created to confirm its classification accuracy [52].

Taken together these four deficits within the literature using logistic regression models, drastically lowers confidence in the overall findings that offense history or crash history can accurately predict future FSI crashes.

### Critical summary of other modeling techniques utilized in this review

Three studies used a modeling technique other than logistic regression: survival analysis [31], Poisson multi-level growth curve modeling [48], and structural equation modeling [50]. The use of survival analysis was largely responsible for Lui and Marchbanks article [31] receiving a high-quality score, with many statistical considerations not being applicable. However, lacking within the study was a description of how raw data was treated, whether preliminary analyses were conducted, and the reporting of descriptive statistics. These limitations reduce the generalization and utility of the model.

A Poisson multi-level growth curve model was used by Stringer [48] to address the question, “What is the probability that in a given period an FSI crash will occur?” Stringer [48] received the highest quality score among the included studies, suggesting that this paper was the most rigorous. The weaknesses identified in Stringer [48] did not seriously undermine the validity of the results. The most important result was based on longitudinal data; crash history and offense history significantly predict *future* fatal crashes with crash history being the more important predictor. Weaknesses were failure to report the statistical packages used and descriptive statistics for the included variables. In addition, there was no model validation attempt so the extent to which the model has general application is unknown. Notable strengths of the study were the use of a Poisson distribution to avoid overestimation of zero values, the use of longitudinal data, and rigorous checks of the model assumptions.

Yuan et al. [50] used structural equation modeling to make sense of a broad range of variables involved in FSI crashes. Structural equation modeling allows for the modeling of latent constructs using measurement models and for path models used to assess relationships between variables that are measured (observed) or latent (not observed) or both. An example of a latent variable constructed in this paper is “Driver Factors” made up from the following measured variables-seatbelt use, driving expertise, offense history, crash history, and gender (measurement model). “Driver Factors” was used to predict two other latent variables, “Truck Occupant” and “Accident Size,” using a path model. This technique was appropriately used by Yuan et al. (2019) as they had a large data set, appropriate measures for the latent variables which met the underlying model assumptions, and appropriate values for the goodness of fit indices.

## Recommendations for future research of FSI crashes using offense history and crash history as predictor variables

There are multiple recommendations for future statistical modeling of FSI crashes from offense and crash history based on the reviewed literature. Firstly, when using population data, and data containing a large number of variables, the use of statistical significance indicators such as p-values are inappropriate. For example, tests of predictor coefficients will inevitably result in multiple type 1 errors. Instead, researchers need to follow and report rigorous methods of variable selection prior to commencing the main analysis. Secondly, future research needs to test and report the checking of statistical assumptions and models need to be validated using fresh data. In addition, classification accuracy needs to be reported considering any imbalance in the crash data. The underreporting of crashes has previously been argued to create a non-random data set, violating traditional statistical assumptions [20], but this is unlikely for FSI crashes.

Thirdly, machine learning should be utilized to overcome the problem of unwanted correlations in the data when there are very large sample sizes. These methods do not rely on *p* values for model selection purposes. For example, random forest can accommodate interactions between predictor variables and can sort the predictor variables according to their importance [21]. Importance ranking is crucial when there are many predictor variables and there is a need for organizations to identify the most important variables for policy changes. Similarly, to random forest models, gradient boosting algorithms may be useful in that they can handle large sets of predictor variables and interactions. The advantage of these models is their ability to more accurately classify minority classes. However, careful considerations for the tuning of the parameters during the training phase, often involving cross-validation, is needed in order to avoid overfitting. Unfortunately, interpretation of random forests and gradient boosting is difficult. This makes the results harder to explain to non-technical audiences compared to the results of traditional statistical models such as logistic regression models which provide odds ratios with 95% confidence intervals.

Another prominent issue faced by researchers analyzing FSI crash data is the prevalence of highly imbalanced data sets with respect to the class of the dependent variable. Most drivers have no history of being involved in an FSI crash. This imbalance in the number of drivers who have and who have not been involved in an FSI crash is problematic for statistical models attempting to classify drivers as being at risk of FSI crash involvement as models developed using imbalanced data tend to struggle to correctly classify those in the minority class (i.e., drivers most at risk of FSI crash involvement). In order to overcome this problem, researchers should consider using techniques such as the synthetic minority over sampling technique (SMOTE) which allows oversampling of the minority class in a representative manner. Another consideration for researchers is the use of hidden Markov models or recurrent neural networks to identify drivers more a risk of an FSI crash. These methods could use the past sequence of offenses to predict the probability of a future FSI crash.

Finally, there is a need for the use of statistical quality assessment tools in future reviews of the crash literature. It is hoped that future reviews of the FSI and broader crash and transportation literature will refine and use our tool to assess the quality of statistical analyses. This is important because a failure to identify poor statistical analysis in reviews may lead to incorrect conclusions and misinformed policy. The use of a standardized assessment tool improves the objectivity of a review’s findings. Such tools are easy and quick to use, also allowing comparisons between reviewer scores and ensuring that all studies are assessed on the same characteristics.

### Limitations of this review

Language and geographical bias limited this review. Similar to other reviews of related topics [20, 53], the bulk of the research was conducted in the USA. We considered only articles written in English and the grey literature review was confined to higher-income countries with lower road death rates. The review was limited to a qualitative synthesis of included studies as a meta-analysis was deemed inappropriate. This was because most models reviewed included many predictor variables in addition to our predictors of interest, namely, offense history and crash history. Further limitations were the time and scope of the search protocol. Despite searching four prominent databases, it is possible that further applicable publications were missed. The lack of search terms for specific offenses (e.g., “speeding,” “drink driving”) may have limited the identification of additional studies present in the databases that were searched. Additionally, one author conducted the screening of identified records and abstracts potentially introducing bias and missed studies. The applicability of our findings is limited to high income developed countries, as FSI crashes are not rare and offense history may not be reliable in countries with low and middle incomes.

## Conclusions

This review contributed to the literature in multiple ways. The study developed a statistical quality assessment tool and demonstrated how it can be utilized when presenting evidence of FSI crash prediction. The review identified that multicollinearity, model validation, and appropriate methods for the selection of predictor variables remain problematic in studies predicting FSI crashes from offense and crash history. However, the most recent studies reported more rigorous modeling practices. Future studies modeling FSI crash risk using offense and/or crash history should consider employing machine learning methods to overcome some of the key limitations of the traditionally used statistical techniques identified in this review. Seven out of the 15 studies using logistic regression reported an association between offense history and FSI crashes. Suspension bans and crash history were also commonly reported as having an association with FSI crashes.

## Supplementary information

**Additional file 1.** Data base search strings 7/6/2019. Appendix S1-S6

## Data Availability

All data analyzed during this study are included in this published article and its supplementary contents.

## Abbreviations

FSI: Fatal and serious injury
PRISMA: Preferred Reporting Items for Systematic Reviews and Meta-analyses
PROSPERO: International prospective register of systematic reviews
ATRI: Australian transport index
ITRD: Transportation Research International Documentation database
SAMPL: Statistical Analysis and Methods in the Published Literature
SM: Samuel Muir
SS: S. S. M. Silva
DM: Denny Myer
TIFA database: Trucks Involved in Fatal Accidents database
FARS: Fatality Analysis Reporting System
BIFA: Buses Involved in Fatal Accidents database
KABCO scale: Fatal, incapacitating injury, non-incapacitating injury, possible injury, property damage only scale
MCMIS: Motor Carrier Management Information System database
CDLIS: Commercial Driver’s License Information System
NDDL: North Dakota Drivers’ License data
TARS: Traffic Accident Reporting System
NPTS: Nationwide Personal Transportation Survey
VALT: Traffic Safety Committee of Insurance Companies
GSS: General Social Survey from National Opinion Research Center
ICBS: Israel Central Bureau of Statistics
CARE: Critical Analysis Reporting Environment
WCRVD: Wyoming Court Reported Violation Database
DUI: Driving under the influence
DWI: Driving while intoxicated
UDA: Unsafe driving action
USA: United States of America
