# Supplementary material for: A systematic review of statistical models and outcomes of predicting fatal and serious injury crashes from driver crash and offense history data
Source: Syst Rev. 2020 Sep 28;9:220. doi: 10.1186/s13643-020-01475-7 (PMC7523043; doi:10.1186/s13643-020-01475-7)
Supplement: Supplementary file 1 — Additional file 1. Data base search strings 7/6/2019. Appendix S1-S6 [file 13643_2020_1475_MOESM1_ESM.docx]

**Appendix S1: Data base search strings 7/6/2019**

- **Australian transport index: ATRI data base (via informit) = 191**

(prediction OR model*) AND (crash OR accident OR collision) AND (driver) AND (traffic OR road OR lane*) AND (OR fatal* OR death) (from 1984)

- **Transportation Research International Documentation (ITRD) database = 502**

Includes records from Transportation Research Information Services (TRIS) and the OECD’s joint Transport Research Centre’s International Transport Research Documentation data base (ITRD).

(predict* OR model*) AND (crash OR accident OR collision) AND (driver) AND (road) AND (fatal* OR death) (English) (from 1984)

- **Scopus = 430**

(predict* OR model) AND (“serious crash*” OR “serious accident” OR “serious collision” OR “fatal accident” OR “fatal crash” OR “fatal collision” OR “road deaths” OR “road fatal*” OR “traffic fatal*” OR “collision fatal*” OR “accident fatal*”) AND (driver) (since 1984)

- **Web of science = 269**

(predict* OR model) AND (“serious crash*” OR “serious accident” OR “serious collision” OR “fatal accident” OR “fatal crash” OR “fatal collision” OR “road deaths” OR “road fatal*” OR “traffic fatal*” OR “collision fatal*” OR “accident fatal*”) AND (driver) (from 1984)

**Appendix S2: Grey literature search**

**High income level countries with a death rate of ≤ 10 deaths per 100,000**

<http://gamapserver.who.int/gho/interactive_charts/road_safety/road_traffic_deaths2/atlas.html>

International sites:

<http://www.oecd.org/>

<https://etsc.eu/euroadsafetydata/>

<https://www.who.int/news-room/fact-sheets/detail/road-traffic-injuries>

<https://www.asirt.org/safe-travel/road-safety-facts/>

<https://www.cdc.gov/features/globalroadsafety/index.html>

<https://www.itf-oecd.org/>

Antigua and Barbuda:

<https://ab.gov.ag/>

Australia:

[https://www.bitre.gov.au](https://www.bitre.gov.au/statistics/safety/)

[https://www.ntc.gov.au](https://www.ntc.gov.au/)

<https://www.tac.vic.gov.au/>

Austria:

<https://www.bundeskanzleramt.gv.at/>

Barbados:

<https://www.gov.bb/>

Belgium:

<https://www.belgium.be/en>

Canada:

<http://www.tc.gc.ca/en/services/road.html>

Croatia:

[https://mup.gov.hr/UserDocsImages//dokumenti//14_Prilog4_NPSCPeng_16_02_15GracDarko.pdf](https://mup.gov.hr/UserDocsImages/dokumenti/14_Prilog4_NPSCPeng_16_02_15GracDarko.pdf)

<https://vlada.gov.hr/documents/15140>

Cyprus:

[http://www.cyprus.gov.cy](http://www.cyprus.gov.cy/)

Czech Republic:

<https://www.vlada.cz/en/>

Denmark:

<http://um.dk/en/>

<https://www.dst.dk/en>

Estonia:

<https://www.stat.ee/34674>

Finland:

<http://www.stat.fi/index_en.html>

France:

<https://www.gouvernement.fr/en/news>

Germany:

<https://www.bundesregierung.de/breg-en>

Greece:

<https://www.hellenicparliament.gr/en/>

Iceland:

<https://www.government.is/>

Ireland:

<https://www.gov.ie/en/>

Israel:

<https://www.gov.il/en/>

Italy:

<http://www.camera.it/leg18/1>

<https://www.istat.it/en/>

Japan:

<https://www.japan.go.jp/>

<https://www.stat.go.jp/english/>

Latvia:

<https://www.mk.gov.lv/en>

Lithuania:

<https://lrv.lt/en>

Luxembourg:

<http://luxembourg.public.lu/en/>

Malta:

<https://www.gov.mt/en/Pages/gov.mt%20homepage.aspx>

Netherlands:

<https://www.government.nl/government>

<https://www.cbs.nl/>

New Zealand:

<https://www.govt.nz/>

<https://www.stats.govt.nz/>

Norway:

<https://www.regjeringen.no/en/id4/>

<https://www.ssb.no/en>

Poland:

<https://stat.gov.pl/>

Portugal:

<https://ine.pt/xportal/xmain?xpgid=ine_main&xpid=INE>

Qatar:

Republic of Korea:

<http://kostat.go.kr/portal/eng/index.action>

<https://data.oecd.org/korea.htm>

San Marino:

<http://www.sanmarino.sm/on-line/en/home.html>

Singapore:

<https://www.singstat.gov.sg/>

Slovakia:

[https://slovak.statistics.sk](https://slovak.statistics.sk/)

Slovenia:

<https://www.stat.si/statweb/en/home>

Spain:

<https://www.ine.es/en/>

<https://data.oecd.org/spain.htm>

Sweden:

<https://www.scb.se/en/>

<https://data.oecd.org/sweden.htm>

Switzerland:

<https://www.bfs.admin.ch/bfs/en/home/fso/official-statistics.html>

<https://data.oecd.org/switzerland.htm>

United Kingdom:

<https://www.ons.gov.uk/>

<https://www.gov.uk/government/statistics>

**Appendix S3: Results of the forward and backwards search**

**107. Exploring traffic safety culture and drunk driving: An examination of the community and DUI related fatal crashes in the U.S. (1993–2015).** [**Stringer, R.J.**](https://www-scopus-com.ezproxy.lib.swin.edu.au/authid/detail.uri?origin=resultslist&authorId=57190745354&zone=) **2018.** [**Transportation Research Part F: Traffic Psychology and Behaviour**](https://www-scopus-com.ezproxy.lib.swin.edu.au/sourceid/20897?origin=resultslist)**, 56, pp. 371-380**

Reference search (74): 0

Citation search (1): 0

**123. Role of alcohol and marijuana use in the initiation of fatal two-vehicle crashes.** [**Li, G.**](https://www-scopus-com.ezproxy.lib.swin.edu.au/authid/detail.uri?origin=resultslist&authorId=55547060800&zone=)**,**[**Chihuri, S.**](https://www-scopus-com.ezproxy.lib.swin.edu.au/authid/detail.uri?origin=resultslist&authorId=57063490100&zone=)**,**[**Brady, J.E.**](https://www-scopus-com.ezproxy.lib.swin.edu.au/authid/detail.uri?origin=resultslist&authorId=15828779600&zone=) **2017,** [**Annals of Epidemiology**](https://www-scopus-com.ezproxy.lib.swin.edu.au/sourceid/19569?origin=resultslist)**, 27(5), pp. 342-347.e1**

Reference search (53): 0

Citation search (4): 0

**132. Child restraint use and driver screening in fatal crashes involving drugs and alcohol.** [**Huang, Y.**](https://www-scopus-com.ezproxy.lib.swin.edu.au/authid/detail.uri?origin=resultslist&authorId=57191040939&zone=)**,**[**Liu, C.**](https://www-scopus-com.ezproxy.lib.swin.edu.au/authid/detail.uri?origin=resultslist&authorId=57191037641&zone=)**,**[**Pressley, J.C.**](https://www-scopus-com.ezproxy.lib.swin.edu.au/authid/detail.uri?origin=resultslist&authorId=6701413252&zone=) **2016.** [**Pediatrics**](https://www-scopus-com.ezproxy.lib.swin.edu.au/sourceid/15756?origin=resultslist)**, 138(3),e20160319**

Reference search (23): 0

Citation search (4): 0

**143. The combined effects of alcohol and cannabis on driving: Impact on crash risk.** [**Dubois, S.**](https://www-scopus-com.ezproxy.lib.swin.edu.au/authid/detail.uri?origin=resultslist&authorId=7005784023&zone=)**,**[**Mullen, N.**](https://www-scopus-com.ezproxy.lib.swin.edu.au/authid/detail.uri?origin=resultslist&authorId=24402570600&zone=)**,**[**Weaver, B.**](https://www-scopus-com.ezproxy.lib.swin.edu.au/authid/detail.uri?origin=resultslist&authorId=7102533570&zone=)**, [Bédard, M.](https://www-scopus-com.ezproxy.lib.swin.edu.au/authid/detail.uri?origin=resultslist&authorId=35594547000&zone=) 2015,** [**Forensic Science International**](https://www-scopus-com.ezproxy.lib.swin.edu.au/sourceid/27743?origin=resultslist)**, 248, pp. 94-100**

Reference search (58): 0

Citation search (39): 0

**167. The influence of stimulants on truck driver crash responsibility in fatal crashes.** [**Gates, J.**](https://www-scopus-com.ezproxy.lib.swin.edu.au/authid/detail.uri?origin=resultslist&authorId=55657844600&zone=)**,**[**Dubois, S.**](https://www-scopus-com.ezproxy.lib.swin.edu.au/authid/detail.uri?origin=resultslist&authorId=7005784023&zone=)**,**[**Mullen, N.**](https://www-scopus-com.ezproxy.lib.swin.edu.au/authid/detail.uri?origin=resultslist&authorId=24402570600&zone=)**,**[**Weaver, B.**](https://www-scopus-com.ezproxy.lib.swin.edu.au/authid/detail.uri?origin=resultslist&authorId=7102533570&zone=)**, [Bédard, M.](https://www-scopus-com.ezproxy.lib.swin.edu.au/authid/detail.uri?origin=resultslist&authorId=35594547000&zone=) 2013,** [**Forensic Science International**](https://www-scopus-com.ezproxy.lib.swin.edu.au/sourceid/27743?origin=resultslist)**, 228(1-3), pp. 15-20**

Reference search (36): 0

Citation search (22): 1

244. High prevalence of previous arrests for illicit drug use and/or impaired driving among drivers killed in motor vehicle crashes in Sweden with amphetamine in blood at autopsy. [Jones, A. W.](https://www-scopus-com.ezproxy.lib.swin.edu.au/authid/detail.uri?authorId=7407102740&amp;eid=2-s2.0-84937525872) [Holmgren, A.](https://www-scopus-com.ezproxy.lib.swin.edu.au/authid/detail.uri?authorId=23004527100&amp;eid=2-s2.0-84937525872), [Ahlner, J.](https://www-scopus-com.ezproxy.lib.swin.edu.au/authid/detail.uri?authorId=24438918600&amp;eid=2-s2.0-84937525872) [International Journal of Drug Policy](https://www-scopus-com.ezproxy.lib.swin.edu.au/sourceid/25290?origin=recordpage), Volume 26, Issue 8, 1 August 2015, Pages 790-793

**189. Factors associated with automobile accidents and survival.** [**Kim, H.S.**](https://www-scopus-com.ezproxy.lib.swin.edu.au/authid/detail.uri?origin=resultslist&authorId=7410128767&zone=)**,**[**Kim, H.J.**](https://www-scopus-com.ezproxy.lib.swin.edu.au/authid/detail.uri?origin=resultslist&authorId=34770459800&zone=)**,**[**Son, B.**](https://www-scopus-com.ezproxy.lib.swin.edu.au/authid/detail.uri?origin=resultslist&authorId=8985750900&zone=) **2006,** [**Accident Analysis and Prevention**](https://www-scopus-com.ezproxy.lib.swin.edu.au/sourceid/19532?origin=resultslist)**, 38(5), pp. 981-987**

Reference search (13): 0

Citation search (14): 0

**225. THE CONNECTION BETWEEN RISKY DRIVING AND INVOLVEMENT IN FATAL ACCIDENTS. By:**[**RAJALIN, S**](http://apps.webofknowledge.com.ezproxy.lib.swin.edu.au/DaisyOneClickSearch.do?product=WOS&search_mode=DaisyOneClickSearch&colName=WOS&SID=C5jzgzYMvSSmhbkgBbF&author_name=RAJALIN,%20S&dais_id=4260062&excludeEventConfig=ExcludeIfFromFullRecPage)**.** [**ACCIDENT ANALYSIS AND PREVENTION**](javascript:;)**Volume: 26   Issue: 5   Pages: 555-562   Published: OCT 1994**

Reference search (29): 1

Citation search (68): 4

245. [Estimating the accident potential of an Ontario driver](https://www-scopus-com.ezproxy.lib.swin.edu.au/record/display.uri?eid=2-s2.0-0026143173&origin=reflist&sort=plf-f&cite=2-s2.0-0028523227&src=s&imp=t&sid=88458d68ea412c14ac352f674c79ad6b&sot=cite&sdt=a&sl=0&recordRank=). Hauer, E., Persaud, B.N., Smiley, A., Duncan, D. (1991) Accident Analysis and Prevention, 23 (2-3), pp. 133-152. doi: 10.1016/0001-4575(91)90044-6

## 246. The effect of traffic tickets on road traffic crashes. Factor, R. [Accident Analysis and Prevention](https://www-scopus-com.ezproxy.lib.swin.edu.au/sourceid/19532?origin=recordpage), Volume 64, March 2014, Pages 86-91

247. [Risky driving and recorded driving offences: A 24-year follow-up study](https://www-scopus-com.ezproxy.lib.swin.edu.au/record/display.uri?eid=2-s2.0-84906830342&origin=resultslist&sort=plf-f&cite=2-s2.0-0028523227&src=s&imp=t&sid=88458d68ea412c14ac352f674c79ad6b&sot=cite&sdt=a&sl=0&relpos=16&citeCnt=8&searchTerm=).  [Summala, H.](https://www-scopus-com.ezproxy.lib.swin.edu.au/authid/detail.uri?origin=resultslist&authorId=56246028500&zone=), [Rajalin, S.](https://www-scopus-com.ezproxy.lib.swin.edu.au/authid/detail.uri?origin=resultslist&authorId=6602737330&zone=), [Radun, I.](https://www-scopus-com.ezproxy.lib.swin.edu.au/authid/detail.uri?origin=resultslist&authorId=8541783200&zone=) 2014. [Accident Analysis and Prevention](https://www-scopus-com.ezproxy.lib.swin.edu.au/sourceid/19532?origin=resultslist) 73, pp. 27-33

248. [Annual mileage, driving violations, and accident involvement in relation to drivers' sex, age, and level of education](https://www-scopus-com.ezproxy.lib.swin.edu.au/record/display.uri?eid=2-s2.0-0033190728&origin=resultslist&sort=plf-f&cite=2-s2.0-0028523227&src=s&nlo=&nlr=&nls=&imp=t&sid=88458d68ea412c14ac352f674c79ad6b&sot=cite&sdt=a&sl=0&relpos=58&citeCnt=102&searchTerm=). [Lourens, P.F.](https://www-scopus-com.ezproxy.lib.swin.edu.au/authid/detail.uri?origin=resultslist&authorId=6602138861&zone=), [Vissers, J.A.M.M.](https://www-scopus-com.ezproxy.lib.swin.edu.au/authid/detail.uri?origin=resultslist&authorId=57197439048&zone=), [Jessurun, M.](https://www-scopus-com.ezproxy.lib.swin.edu.au/authid/detail.uri?origin=resultslist&authorId=6506724992&zone=) 1999. [Accident Analysis and Prevention](https://www-scopus-com.ezproxy.lib.swin.edu.au/sourceid/19532?origin=resultslist), 31(5), pp. 593-597

249. [The relationship between speeding behaviour (as measured by violation convictions) and crash involvement](https://www-scopus-com.ezproxy.lib.swin.edu.au/record/display.uri?eid=2-s2.0-0037750356&origin=resultslist&sort=plf-f&cite=2-s2.0-0028523227&src=s&nlo=&nlr=&nls=&imp=t&sid=88458d68ea412c14ac352f674c79ad6b&sot=cite&sdt=a&sl=0&relpos=64&citeCnt=46&searchTerm=). [Cooper, P.J.](https://www-scopus-com.ezproxy.lib.swin.edu.au/authid/detail.uri?origin=resultslist&authorId=55414815000&zone=). 1997. [Journal of Safety Research](https://www-scopus-com.ezproxy.lib.swin.edu.au/sourceid/29284?origin=resultslist), 28(2), pp. 83-95

**81. Impact of enforcement on traffic accidents and fatalities: A multivariate multilevel analysis. Safety Science, Volume 46, Issue 5, 2008, pp 738-750**

Reference search (22): 0

Citation search (32): 0

**127. State-level comparison of traffic fatality data in consideration of marijuana laws.** [**Hamzeie, R.**](https://www-scopus-com.ezproxy.lib.swin.edu.au/authid/detail.uri?origin=resultslist&authorId=57193604872&zone=)**,**[**Thompson, I.**](https://www-scopus-com.ezproxy.lib.swin.edu.au/authid/detail.uri?origin=resultslist&authorId=57196954203&zone=)**,**[**Roy, S.**](https://www-scopus-com.ezproxy.lib.swin.edu.au/authid/detail.uri?origin=resultslist&authorId=57196941191&zone=)**,**[**Savolainen, P.T.**](https://www-scopus-com.ezproxy.lib.swin.edu.au/authid/detail.uri?origin=resultslist&authorId=35410522800&zone=) **2017,** [**Transportation Research Record**](https://www-scopus-com.ezproxy.lib.swin.edu.au/sourceid/27418?origin=resultslist)**, 2660, pp. 78-85**

Reference search (19): 0

Citation search (1): 0

**146. Effects of demographic and driver factors on single-vehicle and multivehicle fatal crashes investigation with multinomial logistic regression.** [**Kumfer, W.**](https://www-scopus-com.ezproxy.lib.swin.edu.au/authid/detail.uri?origin=resultslist&authorId=57189298915&zone=)**,**[**Wei, D.**](https://www-scopus-com.ezproxy.lib.swin.edu.au/authid/detail.uri?origin=resultslist&authorId=55957897400&zone=)**,**[**Liu, H.**](https://www-scopus-com.ezproxy.lib.swin.edu.au/authid/detail.uri?origin=resultslist&authorId=7409748749&zone=) **2015,** [**Transportation Research Record**](https://www-scopus-com.ezproxy.lib.swin.edu.au/sourceid/27418?origin=resultslist)**, 2518, pp. 37-45**

Reference search (29): 1

Citation search (2): 0

250. [Crash involvement of drivers with multiple crashes](https://www-scopus-com.ezproxy.lib.swin.edu.au/record/display.uri?eid=2-s2.0-33644887444&origin=reflist&sort=plf-f&src=s&st1=Effects+of+demographic+and+driver+factors+on+single&st2=&sid=956ec9ffdc2f8c7acab0cd10e84d7d7e&sot=b&sdt=b&sl=58&s=TITLE%28Effects+of+demographic+and+driver+factors+on+single%29&recordRank=). Chandraratna, S., Stamatiadis, N., Stromberg, A. (2006) Accident Analysis and Prevention, 38 (3), pp. 532-541. [Cited 29 times](https://www-scopus-com.ezproxy.lib.swin.edu.au/search/submit/citedby.uri?eid=2-s2.0-84980383413&refeid=2-s2.0-33644887444&src=s&origin=reflist&refstat=core). doi: 10.1016/j.aap.2005.11.011

**151. Examining the impact of opioid analgesics on crash responsibility in truck drivers involved in fatal crashes.** [**Reguly, P.**](https://www-scopus-com.ezproxy.lib.swin.edu.au/authid/detail.uri?origin=resultslist&authorId=55955399400&zone=)**,**[**Dubois, S.**](https://www-scopus-com.ezproxy.lib.swin.edu.au/authid/detail.uri?origin=resultslist&authorId=7005784023&zone=)**, [Bédard, M.](https://www-scopus-com.ezproxy.lib.swin.edu.au/authid/detail.uri?origin=resultslist&authorId=35594547000&zone=) 2014,** [**Forensic Science International**](https://www-scopus-com.ezproxy.lib.swin.edu.au/sourceid/27743?origin=resultslist)**, 234, pp. 154-161**

Reference search (51): 0

Citation search (14): 0

**176. Type of Motor Carrier and driver history in fatal bus crashes.** [**Blower, D.**](https://www-scopus-com.ezproxy.lib.swin.edu.au/authid/detail.uri?origin=resultslist&authorId=8844546000&zone=)**,**[**Green, P.E.**](https://www-scopus-com.ezproxy.lib.swin.edu.au/authid/detail.uri?origin=resultslist&authorId=36999771600&zone=) **2010,** [**Transportation Research Record**](https://www-scopus-com.ezproxy.lib.swin.edu.au/sourceid/27418?origin=resultslist)**, (2194), pp. 37-43**

Reference search (7): 0

Citation search (16): 0

**2. Analysis of the Risk Factors Affecting the Size of Fatal Accidents Involving Trucks Based on the Structural Equation Model. Transportation Research Record: Journal of the Transportation Research Board, Issue 0, 2019**

Reference search (28): 0

Citation search (0): 0

**30. Risk factors affecting fatal bus accident severity: Their impact on different types of bus drivers. Accident Analysis & Prevention, Volume 86, 2016, pp 29-39**

Reference search (80): 1

Citation search (20): 0

251. [Previous convictions or accidents and the risk of subsequent accidents of older drivers](https://www-scopus-com.ezproxy.lib.swin.edu.au/record/display.uri?eid=2-s2.0-0036489132&origin=reflist&sort=plf-f&src=s&st1=Risk+factors+affecting+fatal+bus+accident+severity%3a+Their+impact+on+different+types+of+bus+drivers&st2=&sid=fee734b0ffc10df576335ee252571d98&sot=b&sdt=b&sl=105&s=TITLE%28Risk+factors+affecting+fatal+bus+accident+severity%3a+Their+impact+on+different+types+of+bus+drivers%29&recordRank=). Daigneault, G., Joly, P., Frigon, J.-Y. (2002) Accident Analysis and Prevention, 34 (2), pp. 257-261. doi: 10.1016/S0001-4575(01)00014-8

**64. Predicting Truck Crash Involvement: Linking Driver Behaviors to Crash Probability. Journal of Transportation Law, Logistics and Policy, Volume 78, Issue 2, 2011, pp 109-128**

Reference search (7): 0

Citation search (?): ?

**65. Identifying Factors That Predict Teen Driver Crashes. (2011). Malchose, D and Vachal, K. Rural Transportation Safety and Security Center, Upper Great Plains Transportation Institute, North Dakota State University.**

Reference search (23): 1

Citation search (?): ?

## 252. A study of the time between previous traffic infractions and fatal automobile crashes, 1984–1986. [K.-J.Lui](https://www-sciencedirect-com.ezproxy.lib.swin.edu.au/science/article/pii/002243759090001R#!)^[1](https://www-sciencedirect-com.ezproxy.lib.swin.edu.au/science/article/pii/002243759090001R#!)^[P.A.Marchbanks](https://www-sciencedirect-com.ezproxy.lib.swin.edu.au/science/article/pii/002243759090001R#!)^[2](https://www-sciencedirect-com.ezproxy.lib.swin.edu.au/science/article/pii/002243759090001R#!)^. [Journal of Safety Research](https://www-sciencedirect-com.ezproxy.lib.swin.edu.au/science/journal/00224375). [Volume 21, Issue 2](https://www-sciencedirect-com.ezproxy.lib.swin.edu.au/science/journal/00224375/21/2), Summer 1990, Pages 45-51

**86. Relationships between prior driving record, driver culpability, and fatal crash involvement. ROAD SAFETY RESEARCH, POLICING AND EDUCATION CONFERENCE, 2004, PERTH, WESTERN AUSTRALIA, AUSTRALIA, Volume 1, 2004, 10P**

Reference search (22): 0

Citation search (?): ?

**242. A comprehensive study of single and multiple truck crashes using violation and crash data Mashhadi, M.M.R., Wulff, S.S., Ksaibati, K. 2018. Open Transportation Journal. 12, pp. 43-56**

Reference search (40): 0

Citation search (?): ?

**199. The driver's role in fatal two-car crashes: A paired "case-control" study.** [**Perneger, T.**](https://www-scopus-com.ezproxy.lib.swin.edu.au/authid/detail.uri?origin=resultslist&authorId=7102259897&zone=)**,**[**Smith, G.S.**](https://www-scopus-com.ezproxy.lib.swin.edu.au/authid/detail.uri?origin=resultslist&authorId=7406738484&zone=) **1991,** [**American Journal of Epidemiology**](https://www-scopus-com.ezproxy.lib.swin.edu.au/sourceid/27058?origin=resultslist)**, 134(10), pp. 1138-1145**

Reference search (27): 0

Citation search (?): ?

**Appendix S4: Database full text research results**

# [Factors contributing to injury severity in work zone related crashes in New Zealand](https://trid.trb.org/View/1596228)

2019-02, International Journal of Sustainable Transportation, Volume 13, Issue 2, 2019, pp 148-154

# [Analysis of the Risk Factors Affecting the Size of Fatal Accidents Involving Trucks Based on the Structural Equation Model](https://trid.trb.org/View/1592119)

Transportation Research Record: Journal of the Transportation Research Board, Issue 0, 2019

# [Investigating Problem of Distracted Drivers on Louisiana Roadways](https://trid.trb.org/View/1577029)

TRID, 2018, 58p

# [Application of multinomial and ordinal logistic regression to model injury severity of truck crashes, using violation and crash data](https://trid.trb.org/View/1571022)

Journal of Modern Transportation, Volume 26, Issue 4, 2018, pp 268-277

# [Machine Learning Methods to Analyze Injury Severity of Drivers from Different Age and Gender Groups](https://trid.trb.org/View/1495782)

Transportation Research Record: Journal of the Transportation Research Board, Volume 2672, Issue 38, 2018, pp 171-183

# [Design and experiment verification of a novel analysis framework for recognition of driver injury patterns: From a multi-class classification perspective](https://trid.trb.org/View/1538355)

Accident Analysis & Prevention, Volume 120, Issue 0, 2018, pp 152-164

# [Examining driver injury severity in intersection-related crashes using cluster analysis and hierarchical Bayesian models](https://trid.trb.org/View/1532832)

Accident Analysis & Prevention, Volume 120, Issue 0, 2018, pp 139-151

# [Wrong-way driving crashes: A random-parameters ordered probit analysis of injury severity](https://trid.trb.org/View/1513990)

Accident Analysis & Prevention, Volume 117, Issue 0, 2018, pp 128-135

# [Spatio-temporal pattern of vulnerable road user’s collisions hot spots and related risk factors for injury severity in Tunisia](https://trid.trb.org/View/1516112)

Transportation Research Part F: Traffic Psychology and Behaviour, Volume 56, 2018, pp 477-495

# [Exploring Crash Characteristics and Injury Outcomes among Older Truck Drivers: An Analysis of Truck-Involved Crash Data in the United States](https://trid.trb.org/View/1511024)

Safety Science, Volume 106, Issue 0, 2018, pp 140-145

# [Gendered analysis of fatal crashes among young drivers in Alabama, USA](https://trid.trb.org/View/1527911)

Safety, Volume 4, Issue 3, 2018, Article ID 29

# [Investigation of Gender Differences in Large-Truck Crash Injury Severity in Missouri](https://trid.trb.org/View/1522218)

TRID, 2018, 32p

# [Prevalence of Alcohol Impairment and Odds of a Driver Injury or Fatality in On-Road Farm Equipment Crashes](https://trid.trb.org/View/1505492)

Traffic Injury Prevention, Volume 19, Issue 3, 2018, pp 230-234

# [Aging Road User Survey and Crash Analysis to Identify Issues and Applicable Improvement Strategies for Kansas Conditions](https://trid.trb.org/View/1502834)

TRID, 2018, 119p

# [Identification of significant factors in fatal-injury highway crashes using genetic algorithm and neural network](https://trid.trb.org/View/1498155)

Accident Analysis & Prevention, Volume 111, 2018, pp 354-363

# [Interactions between the built and socio-economic environment and driver demographics: spatial econometric models of car crashes in the Columbus Metropolitan Area](https://trid.trb.org/View/1507857)

International Journal of Urban Sciences, Volume 22, Issue 1, 2018, pp 17-37

# [Contributing Factors to Run-Off-Road Crashes Involving Large Trucks under Lighted and Dark Conditions](https://trid.trb.org/View/1486856)

Journal of Transportation Engineering, Part A: Systems, Volume 144, Issue 1, 2018, Content ID 04017066

# [Forecasting of road accident in the DVRE system](https://trid.trb.org/View/1577566)

Transportation Research Procedia, Volume 36, Issue 0, 2018, pp 380-385

# [Formulating alcohol-influenced driver’s injury severities in intersection-related crashes](https://trid.trb.org/View/1570132)

Transport, Volume 33, Issue 1, 2018, pp 165-176

# [Data-Mining Techniques for Traffic Accident Modeling and Prediction in the United Arab Emirates](https://trid.trb.org/View/1460444)

Journal of Transportation Safety & Security, Volume 9, Issue 2, 2017, pp 146-166

# [Modeling single-vehicle run-off-road crash severity in rural areas: Accounting for unobserved heterogeneity and age difference](https://trid.trb.org/View/1459260)

Accident Analysis & Prevention, Volume 101, 2017, pp 124-134

# [Socioeconomic and Sociodemographic Inequalities and Their Association with Road Traffic Injuries](https://trid.trb.org/View/1467700)

Journal of Transport & Health, Volume 4, 2017, pp 152-161

# [An Application of Decision Tree Models to Examine Motor Vehicle Crash Severity Outcomes](https://trid.trb.org/View/1567472)

Journal of the Transportation Research Forum, Volume 56, Issue 2, 2017, pp 73-91

# [Examining driver injury severity outcomes in rural non-interstate roadway crashes using a hierarchical ordered logit model](https://trid.trb.org/View/1424777)

Accident Analysis & Prevention, Volume 96, 2016, pp 79-87

# [Factors associated with single-vehicle and multi-vehicle road traffic collision injuries in Ireland](https://trid.trb.org/View/1425828)

International Journal of Injury Control and Safety Promotion, Volume 23, Issue 4, 2016, pp 351-361

# [Fatal and serious road crashes involving young New Zealand drivers: a latent class clustering approach](https://trid.trb.org/View/1425836)

International Journal of Injury Control and Safety Promotion, Volume 23, Issue 4, 2016, pp 427-443

# [Comparing the characteristics of the target and bullet vehicle for injury severity in two vehicle crashes](https://trid.trb.org/View/1436035)

Australasian Road Safety Conference, 2016, Canberra, ACT, Australia, 2016, 5p

# [Heterogeneous impacts of gender-interpreted contributing factors on driver injury severities in single-vehicle rollover crashes](https://trid.trb.org/View/1416628)

Accident Analysis & Prevention, Volume 94, 2016, pp 28-34

# [An explanatory analysis of driver injury severity in rear-end crashes using a decision table/Naïve Bayes (DTNB) hybrid classifier](https://trid.trb.org/View/1401020)

Accident Analysis & Prevention, Volume 90, 2016, pp 95-107

# [Risk factors affecting fatal bus accident severity: Their impact on different types of bus drivers](https://trid.trb.org/View/1376891)

Accident Analysis & Prevention, Volume 86, 2016, pp 29-39

# [Correlation of Driver Gender with Injury Severity in Large Truck Crashes in Missouri](https://trid.trb.org/View/1424720)

Transportation Research Record: Journal of the Transportation Research Board, Issue 2585, 2016, pp 49-58

# [Differences in Factors Affecting Various Crash Types with High Numbers of Fatalities and Injuries in China](https://trid.trb.org/View/1420934)

PLoS One, Volume 11, Issue 7, 2016, n.p.

# [What Role Do Precrash Driver Actions Play in Work Zone Crashes? Application of Hierarchical Models to Crash Data](https://trid.trb.org/View/1402298)

Transportation Research Record: Journal of the Transportation Research Board, Issue 2555, 2016, pp 1-11

# [A multinomial logit model-Bayesian network hybrid approach for driver injury severity analyses in rear-end crashes](https://trid.trb.org/View/1355007)

Accident Analysis & Prevention, Volume 80, Issue 0, 2015, pp 76-88

# [Quantifying the influence of safe road systems and legal licensing age on road mortality among young adolescents: Steps towards system thinking](https://trid.trb.org/View/1334921)

Accident Analysis & Prevention, Volume 74, Issue 0, 2015, pp 306-313

# [The Road Traffic Crashes as a Neglected Public Health Concern; An Observational Study From Iranian Population](https://trid.trb.org/View/1326970)

Traffic Injury Prevention, Volume 16, Issue 1, 2015, pp 36-41

# [Analysis of Motorcyclists Driving Behavior in Bandung City](https://trid.trb.org/View/1378350)

Journal of the Eastern Asia Society for Transportation Studies, Volume 11, Issue 0, 2015, pp 2070-2086

# [Examining the Differences Between Contributing Factors Affecting the Severity of Single and Multi-Vehicle Crashes](https://trid.trb.org/View/1338201)

Transportation Research Board 94th Annual Meeting, 2015, 18p

# [Exploring the Nature and Severity of Heavy Truck Crashes in Abu Dhabi, United Arab Emirates](https://trid.trb.org/View/1336692)

Transportation Research Record: Journal of the Transportation Research Board, Issue 2517, 2015, pp 1–9

# [Effects of excessive speeding and falling asleep while driving on crash injury severity in Ethiopia: A generalized ordered logit model analysis](https://trid.trb.org/View/1317221)

Accident Analysis & Prevention, Volume 71, Issue 0, 2014, pp 15-21

# [Explaining Chile's Traffic Fatality and Injury Reduction for 2000–2012](https://trid.trb.org/View/1323959)

Traffic Injury Prevention, Volume 15, Issue sup1, 2014, pp S56-S63

# [Characteristics of the Road and Surrounding Environment in Metropolitan Shopping Strips: Association with the Frequency and Severity of Single-Vehicle Crashes](https://trid.trb.org/View/1323938)

Traffic Injury Prevention, Volume 15, Issue sup1, 2014, pp S74-S80

# [A Comparison of Contributing Factors between Alcohol Related Single Vehicle Motorcycle and Car Crashes](https://trid.trb.org/View/1316448)

Journal of Safety Research, Volume 49, Issue 0, 2014, pp 129-135

# [Analysis of factors associated with injury severity in crashes involving young New Zealand drivers](https://trid.trb.org/View/1304998)

Accident Analysis & Prevention, Volume 65, Issue 0, 2014, pp 142-155

# [Risk Factors Associated with Crash Severity on Low-Volume Rural Roads in Denmark](https://trid.trb.org/View/1263878)

Journal of Transportation Safety & Security, Volume 6, Issue 1, 2014, pp 1-20

# [Exploring the Risk Factors Associated with the Size and Severity of Roadway Crashes in Riyadh](https://trid.trb.org/View/1285674)

Journal of Safety Research, Volume 47, Issue 0, 2013, pp 67-74

# [Comparing single vehicle and multivehicle fatal road crashes: A joint analysis of road conditions, time variables and driver characteristics](https://trid.trb.org/View/1278860)

Accident Analysis & Prevention, Volume 60, Issue 0, 2013, pp 466-471

# [Fatality Trends and Projections for Drivers and Passengers: Differences between Observed and Expected Fatality Rates with a Focus on Older Adults](https://trid.trb.org/View/1258788)

Safety Science, Volume 59, Issue 0, 2013, pp 106-115

# [Analyzing the severity of accidents on the German Autobahn](https://trid.trb.org/View/1253015)

Accident Analysis & Prevention, Volume 57, Issue 0, 2013, pp 40-48

# [An examination of the environmental, driver and vehicle factors associated with the serious and fatal crashes of older rural drivers](https://trid.trb.org/View/1239387)

Accident Analysis & Prevention, Volume 50, Issue 0, 2013, pp 768-775

# [Modeling Injury Outcomes of Crashes Involving Heavy Vehicles on Texas Highways](https://trid.trb.org/View/1243051)

Transportation Research Record: Journal of the Transportation Research Board, Issue 2388, 2013, pp 28–36

# [Holistic Approach to Reduce Rural Roadway Departure Crashes](https://trid.trb.org/View/1241194)

Transportation Research Record: Journal of the Transportation Research Board, Issue 2364, 2013, pp 23–28

# [Aggressive Driving Behaviour in Young Drivers (Aged 16 through 25) Involved in Fatal Crashes](https://trid.trb.org/View/1237952)

Journal of Safety Research, Volume 43, Issue 5-6, 2012, pp 333-338

# [Analysis of factors that increase motorcycle rider risk compared to car driver risk](https://trid.trb.org/View/1238177)

Accident Analysis & Prevention, Volume 49, Issue 0, 2012, pp 23-29

# [Factors Affecting Accident Severity Inside and Outside Urban Areas in Greece](https://trid.trb.org/View/1226325)

Traffic Injury Prevention, Volume 13, Issue 5, 2012, pp 458-467

# [Older drivers' crashes in Queensland, Australia](https://trid.trb.org/View/1147779)

Accident Analysis & Prevention, Volume 48, Issue 0, 2012, pp 423-429

# [An Investigation of the Risk Factors Causing Severe Injuries in Crashes Involving Gravel Trucks](https://trid.trb.org/View/1226313)

Traffic Injury Prevention, Volume 13, Issue 4, 2012, pp 355-363

# [Fatal crash involvement of unlicensed young drivers: County level differences according to material deprivation and urbanicity in the United States](https://trid.trb.org/View/1132881)

Accident Analysis & Prevention, Volume 45, Issue 0, 2012, pp 291-295

# [National Evaluation of the Effect of Graduated Driver Licensing Laws on Teenager Fatality and Injury Crashes](https://trid.trb.org/View/1135236)

Journal of Safety Research, Volume 43, Issue 1, 2012, pp 29-37

# [Commercial Driver Factors in Run-off-Road Crashes](https://trid.trb.org/View/1218132)

Transportation Research Record: Journal of the Transportation Research Board, Issue 2281, 2012, pp 128-132

# [Severity Models of Cross-Median and Rollover Crashes on Rural Divided Highways in Pennsylvania](https://trid.trb.org/View/1125784)

Journal of Safety Research, Volume 42, Issue 5, 2011, pp 375-382

# [Risk-Based Advisory Prevention System for Commercial Trucks Under Hazardous Conditions](https://trid.trb.org/View/1126662)

TRID, 2011, 33p

# [Investigating the Relative Risk Factors of Injuries Caused by Accidents on Roads in the Mashhad Area in 2007](https://trid.trb.org/View/1146799)

Iranian Red Crescent Medical Journal, Volume 13, Issue 8, 2011, 6p

# [Predicting Truck Crash Involvement: Linking Driver Behaviors to Crash Probability](https://trid.trb.org/View/1116834)

Journal of Transportation Law, Logistics and Policy, Volume 78, Issue 2, 2011, pp 109-128

# [Identifying Factors That Predict Teen Driver Crashes](https://trid.trb.org/View/1103939)

TRID, 2011, 29p

# [Factors associated with motorcycle crashes in New South Wales, Australia, 2004-2008](https://trid.trb.org/View/1148690)

Transportation Research Board 90th Annual Meeting, 2011, 16p

# [The Accident Risk–measuring Model for Urban Arterials](https://trid.trb.org/View/1286973)

3rd International Conference on Road Safety and Simulation, 2011, 13p

# [Traffic Violations Versus Driving Errors: Implications for Older Female Drivers](https://trid.trb.org/View/1101769)

Transportation Research Board Conference Proceedings, Volume 2, Issue 46, 2011, pp 55-63

# [Analysis of Large-Truck Crash Severity Using Heteroscedastic Ordered Probit Models](https://trid.trb.org/View/1091940)

Transportation Research Board 90th Annual Meeting, 2011, 30p

# [An Integrated Approach for Studying the Safety of Road Networks: Logistic Regression Models Between Traffic Accident Occurrence and Behavioural, Environmental and Infrastructure Parameters](https://trid.trb.org/View/1095793)

In: The Sustainable World, WIT Press, 2011, pp 525-536

# [The Expected Number of Road Traffic Casualties Using Stratified Data](https://trid.trb.org/View/927727)

Safety Science, Volume 48, Issue 9, 2010, pp 1123-1133

# [Declines in Fatal Crashes of Older Drivers: Changes in Crash Risk and Survivability](https://trid.trb.org/View/923810)

TRID, 2010, 24p

# [Characteristics and Contributory Causes Related to Large Truck Crashes (Phase I)](https://trid.trb.org/View/1537146)

TRID, 2010, 87p

# [Road Crash Trends for Young Drivers in New South Wales, Australia, from 1997 to 2007](https://trid.trb.org/View/914736)

Traffic Injury Prevention, Volume 11, Issue 1, 2010, pp. 8-15

# [Fatality Risk of Intersection Crashes on Rural Undivided Highways in Alberta, Canada](https://trid.trb.org/View/909430)

Transportation Research Record: Journal of the Transportation Research Board, Issue 2148, 2010, pp 107-115

# [The Impact of Underage Drinking Laws on Alcohol-Related Fatal Crashes of Young Drivers](https://trid.trb.org/View/899541)

Alcoholism: Clinical and Experimental Research, Volume 33, Issue 7, 2009, pp 1208-1219

# [Reducing Fatalities and Severe Injuries on Florida’s High-Speed Multi-Lane Arterial Corridors; Part I: Preliminary Severity Analysis of Driver Crash Involvements](https://trid.trb.org/View/894540)

TRID, 2009, 367p

# [The Effect of State Regulations on Truck-Crash Fatalities](https://trid.trb.org/View/890939)

American Journal of Public Health, Volume 99, Issue 3, 2009, pp 408-415

# [A safe road transport system: factors influencing injury outcome for car occupants](https://trid.trb.org/View/905254)

TRID, 2009, 45p+app

# [Use of fatal real-life crashes to analyse a safe road transport system model, including the road user, the vehicle, and the road](https://trid.trb.org/View/1151782)

Traffic Injury Prevention, Volume 9, Issue 5, 2008, 463-71

# [Impact of enforcement on traffic accidents and fatalities: A multivariate multilevel analysis](https://trid.trb.org/View/863485)

Safety Science, Volume 46, Issue 5, 2008, pp 738-750

# [Highway accident severities and the mixed logit model: An exploratory empirical analysis](https://trid.trb.org/View/849727)

Accident Analysis & Prevention, Volume 40, Issue 1, 2008, pp 260-266

# [Ageing Drivers: Storm in a Teacup?](https://trid.trb.org/View/767784)

Accident Analysis & Prevention, Volume 38, Issue 1, 2006, pp 112-121

# [Identification of Factors Leading to High Severity of Crashes in Rural Areas](https://trid.trb.org/View/790355)

Journal of the Transportation Research Forum, Volume 45, Issue 2, 2006, pp 87-101

# [ELDERLY LICENSURE LAWS AND MOTOR VEHICLE FATALITIES](https://trid.trb.org/View/704666)

JAMA: Journal of the American Medical Association, Volume 291, Issue 23, 2004, 7 p.

# [Relationships between prior driving record, driver culpability, and fatal crash involvement](https://trid.trb.org/View/771088)

ROAD SAFETY RESEARCH, POLICING AND EDUCATION CONFERENCE, 2004, PERTH, WESTERN AUSTRALIA, AUSTRALIA, Volume 1, 2004, 10P

# [DEVELOPMENT OF A SAFETY RESOURCE-ALLOCATION MODEL IN MICHIGAN](https://trid.trb.org/View/705180)

Transportation Research Record, Issue 1865, 2004, p. 64-71

# [SLEEP-RELATED FATAL VEHICLE ACCIDENTS: CHARACTERISTICS OF DECISIONS MADE BY MULTIDISCIPLINARY INVESTIGATION TEAMS](https://trid.trb.org/View/704668)

Sleep, Volume 27, Issue 2, 2004, p. 224-227

# [FATALITY RISK ASSESSMENT AND MODELING OF DRIVERS' RESPONSIBILITY FOR CAUSING TRAFFIC ACCIDENTS IN DUBAI](https://trid.trb.org/View/729652)

Journal of Safety Research, Volume 33, Issue 4, 2002, p. 483-496

# [ANALYSIS OF SEVERITY OF YOUNG DRIVER CRASHES: SEQUENTIAL BINARY LOGISTIC REGRESSION MODELING](https://trid.trb.org/View/726702)

Transportation Research Record, Issue 1784, 2002, p. 108-114

# [ANALYSIS OF ALCOHOL-RELATED MOTORCYCLE CRASHES IN FLORIDA AND RECOMMENDED COUNTERMEASURES](https://trid.trb.org/View/717391)

Transportation Research Record, Issue 1779, 2001, p. 189-196

# [POTENTIAL BENEFITS OF RESTRICTIONS ON THE TRANSPORT OF TEENAGE PASSENGERS BY 16 AND 17 YEAR OLD DRIVERS](https://trid.trb.org/View/716982)

Injury Prevention, Volume 7, 2001, p. 129-34

# [LONGITUDINAL ANALYSIS OF FATAL RUN-OFF-ROAD CRASHES, 1975 TO 1997](https://trid.trb.org/View/692578)

Transportation Research Record, Issue 1746, 2001, p. 47-58

# [CRASH INJURY SEVERITY OF OLDER DRIVERS IN IOWA](https://trid.trb.org/View/655626)

Mid-Continent Transportation Symposium 2000, 2000, p. 235-240

# [THE UNSAFE DRIVING ACTS OF MOTORISTS IN THE VICINITY OF LARGE TRUCKS](https://trid.trb.org/View/713337)

TRID, 1999, 34 p.

# [OLDER DRIVERS AND RISK TO OTHER ROAD USERS](https://trid.trb.org/View/577120)

Accident Analysis & Prevention, Volume 29, Issue 5, 1997, p. 573-582

# [STATISTICAL ANALYSIS OF ACCIDENT SEVERITY ON RURAL FREEWAYS](https://trid.trb.org/View/463142)

Accident Analysis & Prevention, Volume 28, Issue 3, 1996, p. 391-401

# [AN EXPLORATORY MULTINOMIAL LOGIT ANALYSIS OF SINGLE-VEHICLE MOTORCYCLE ACCIDENT SEVERITY](https://trid.trb.org/View/469129)

Journal of Safety Research, Volume 27, Issue 3, 1996, p. 183-194

# [Single Vehicle Logging-Related Traffic Crashes in Louisiana from 2010-2015](https://www-scopus-com.ezproxy.lib.swin.edu.au/record/display.uri?eid=2-s2.0-85061206471&origin=resultslist&sort=plf-f&src=s&st1=%28predict*+OR+model%29+AND+%28%22serious+crash*%22+OR+%22serious+accident%22+OR+%22serious+collision%22+OR+%22fatal+accident%22+OR+%22fatal+crash%22+OR+%22fatal+collision%22+OR+%22road+deaths%22+OR+%22road+fatal*%22+OR+%22traffic+fatal*%22+OR+%22collision+fatal*%22+OR+%22accident+fatal*%22%29+AND+%28driver%29+&nlo=&nlr=&nls=&sid=34fc91d90b0a1eb0b79c181c6b17236b&sot=b&sdt=b&sl=290&s=TITLE-ABS-KEY%28%28predict*+OR+model%29+AND+%28%22serious+crash*%22+OR+%22serious+accident%22+OR+%22serious+collision%22+OR+%22fatal+accident%22+OR+%22fatal+crash%22+OR+%22fatal+collision%22+OR+%22road+deaths%22+OR+%22road+fatal*%22+OR+%22traffic+fatal*%22+OR+%22collision+fatal*%22+OR+%22accident+fatal*%22%29+AND+%28driver%29+%29+AND+PUBYEAR+%3e+1983&relpos=2&citeCnt=0&searchTerm=)

[Shipp, E.M.](https://www-scopus-com.ezproxy.lib.swin.edu.au/authid/detail.uri?origin=resultslist&authorId=9745794400&zone=), [Vasudeo, S.](https://www-scopus-com.ezproxy.lib.swin.edu.au/authid/detail.uri?origin=resultslist&authorId=57205700818&zone=), [Trueblood, A.B.](https://www-scopus-com.ezproxy.lib.swin.edu.au/authid/detail.uri?origin=resultslist&authorId=56462849700&zone=), [Garcia, T.P.](https://www-scopus-com.ezproxy.lib.swin.edu.au/authid/detail.uri?origin=resultslist&authorId=55939490400&zone=) 2019 [Journal of Agromedicine](https://www-scopus-com.ezproxy.lib.swin.edu.au/sourceid/29249?origin=resultslist), 24(2), pp. 177-185

# [Extended investigation on road fatality in Brunei](https://www-scopus-com.ezproxy.lib.swin.edu.au/record/display.uri?eid=2-s2.0-85048205040&origin=resultslist&sort=plf-f&src=s&st1=%28predict*+OR+model%29+AND+%28%22serious+crash*%22+OR+%22serious+accident%22+OR+%22serious+collision%22+OR+%22fatal+accident%22+OR+%22fatal+crash%22+OR+%22fatal+collision%22+OR+%22road+deaths%22+OR+%22road+fatal*%22+OR+%22traffic+fatal*%22+OR+%22collision+fatal*%22+OR+%22accident+fatal*%22%29+AND+%28driver%29+&nlo=&nlr=&nls=&sid=34fc91d90b0a1eb0b79c181c6b17236b&sot=b&sdt=b&sl=290&s=TITLE-ABS-KEY%28%28predict*+OR+model%29+AND+%28%22serious+crash*%22+OR+%22serious+accident%22+OR+%22serious+collision%22+OR+%22fatal+accident%22+OR+%22fatal+crash%22+OR+%22fatal+collision%22+OR+%22road+deaths%22+OR+%22road+fatal*%22+OR+%22traffic+fatal*%22+OR+%22collision+fatal*%22+OR+%22accident+fatal*%22%29+AND+%28driver%29+%29+AND+PUBYEAR+%3e+1983&relpos=12&citeCnt=0&searchTerm=)

[Yusof, N.B.](https://www-scopus-com.ezproxy.lib.swin.edu.au/authid/detail.uri?origin=resultslist&authorId=57202388753&zone=), [Hoque, M.A.](https://www-scopus-com.ezproxy.lib.swin.edu.au/authid/detail.uri?origin=resultslist&authorId=14424235000&zone=), [Steele, M.C.](https://www-scopus-com.ezproxy.lib.swin.edu.au/authid/detail.uri?origin=resultslist&authorId=39762645400&zone=), [Yong, S.Y.](https://www-scopus-com.ezproxy.lib.swin.edu.au/authid/detail.uri?origin=resultslist&authorId=57202389475&zone=) 2019. [International Journal of Injury Control and Safety Promotion](https://www-scopus-com.ezproxy.lib.swin.edu.au/sourceid/146160?origin=resultslist)

26(1), pp. 21-29

# [Effectiveness of lowering the blood alcohol concentration (BAC) limit for driving from 0.10 to 0.08 grams per deciliter in the United States](https://www-scopus-com.ezproxy.lib.swin.edu.au/record/display.uri?eid=2-s2.0-85063212127&origin=resultslist&sort=plf-f&src=s&st1=%28predict*+OR+model%29+AND+%28%22serious+crash*%22+OR+%22serious+accident%22+OR+%22serious+collision%22+OR+%22fatal+accident%22+OR+%22fatal+crash%22+OR+%22fatal+collision%22+OR+%22road+deaths%22+OR+%22road+fatal*%22+OR+%22traffic+fatal*%22+OR+%22collision+fatal*%22+OR+%22accident+fatal*%22%29+AND+%28driver%29+&nlo=&nlr=&nls=&sid=34fc91d90b0a1eb0b79c181c6b17236b&sot=b&sdt=b&sl=290&s=TITLE-ABS-KEY%28%28predict*+OR+model%29+AND+%28%22serious+crash*%22+OR+%22serious+accident%22+OR+%22serious+collision%22+OR+%22fatal+accident%22+OR+%22fatal+crash%22+OR+%22fatal+collision%22+OR+%22road+deaths%22+OR+%22road+fatal*%22+OR+%22traffic+fatal*%22+OR+%22collision+fatal*%22+OR+%22accident+fatal*%22%29+AND+%28driver%29+%29+AND+PUBYEAR+%3e+1983&relpos=13&citeCnt=0&searchTerm=)

[Scherer, M.](https://www-scopus-com.ezproxy.lib.swin.edu.au/authid/detail.uri?origin=resultslist&authorId=36884653200&zone=), [Fell, J.C.](https://www-scopus-com.ezproxy.lib.swin.edu.au/authid/detail.uri?origin=resultslist&authorId=7103321984&zone=) 2019. [Traffic Injury Prevention](https://www-scopus-com.ezproxy.lib.swin.edu.au/sourceid/22112?origin=resultslist), 20(1), pp. 1-8

# [Identifying Fatality Risk Factors for the Commercial Vehicle Driver Population](https://www-scopus-com.ezproxy.lib.swin.edu.au/record/display.uri?eid=2-s2.0-85065731694&origin=resultslist&sort=plf-f&src=s&st1=%28predict*+OR+model%29+AND+%28%22serious+crash*%22+OR+%22serious+accident%22+OR+%22serious+collision%22+OR+%22fatal+accident%22+OR+%22fatal+crash%22+OR+%22fatal+collision%22+OR+%22road+deaths%22+OR+%22road+fatal*%22+OR+%22traffic+fatal*%22+OR+%22collision+fatal*%22+OR+%22accident+fatal*%22%29+AND+%28driver%29+&nlo=&nlr=&nls=&sid=34fc91d90b0a1eb0b79c181c6b17236b&sot=b&sdt=b&sl=290&s=TITLE-ABS-KEY%28%28predict*+OR+model%29+AND+%28%22serious+crash*%22+OR+%22serious+accident%22+OR+%22serious+collision%22+OR+%22fatal+accident%22+OR+%22fatal+crash%22+OR+%22fatal+collision%22+OR+%22road+deaths%22+OR+%22road+fatal*%22+OR+%22traffic+fatal*%22+OR+%22collision+fatal*%22+OR+%22accident+fatal*%22%29+AND+%28driver%29+%29+AND+PUBYEAR+%3e+1983&relpos=20&citeCnt=0&searchTerm=)

[Islam, M.](https://www-scopus-com.ezproxy.lib.swin.edu.au/authid/detail.uri?origin=resultslist&authorId=55172619400&zone=), [Ozkul, S.](https://www-scopus-com.ezproxy.lib.swin.edu.au/authid/detail.uri?origin=resultslist&authorId=37000128600&zone=) 2019. [Transportation Research Record](https://www-scopus-com.ezproxy.lib.swin.edu.au/sourceid/27418?origin=resultslist)

# Use of multiple data sources to identify specific drugs and other factors associated with drug and alcohol screening of fatally injured motor vehicle drivers

[Bunn, T.](https://www-scopus-com.ezproxy.lib.swin.edu.au/authid/detail.uri?origin=resultslist&authorId=57203627144&zone=), [Singleton, M.](https://www-scopus-com.ezproxy.lib.swin.edu.au/authid/detail.uri?origin=resultslist&authorId=35992408100&zone=), [Chen, I.-C.](https://www-scopus-com.ezproxy.lib.swin.edu.au/authid/detail.uri?origin=resultslist&authorId=57194143047&zone=) 2019. [Accident Analysis and Prevention](https://www-scopus-com.ezproxy.lib.swin.edu.au/sourceid/19532?origin=resultslist). 122, pp. 287-294

# [Exploring driver injury severity patterns and causes in low visibility related single-vehicle crashes using a finite mixture random parameters model](https://www-scopus-com.ezproxy.lib.swin.edu.au/record/display.uri?eid=2-s2.0-85052959078&origin=resultslist&sort=plf-f&src=s&st1=%28predict*+OR+model%29+AND+%28%22serious+crash*%22+OR+%22serious+accident%22+OR+%22serious+collision%22+OR+%22fatal+accident%22+OR+%22fatal+crash%22+OR+%22fatal+collision%22+OR+%22road+deaths%22+OR+%22road+fatal*%22+OR+%22traffic+fatal*%22+OR+%22collision+fatal*%22+OR+%22accident+fatal*%22%29+AND+%28driver%29+&nlo=&nlr=&nls=&sid=34fc91d90b0a1eb0b79c181c6b17236b&sot=b&sdt=b&sl=290&s=TITLE-ABS-KEY%28%28predict*+OR+model%29+AND+%28%22serious+crash*%22+OR+%22serious+accident%22+OR+%22serious+collision%22+OR+%22fatal+accident%22+OR+%22fatal+crash%22+OR+%22fatal+collision%22+OR+%22road+deaths%22+OR+%22road+fatal*%22+OR+%22traffic+fatal*%22+OR+%22collision+fatal*%22+OR+%22accident+fatal*%22%29+AND+%28driver%29+%29+AND+PUBYEAR+%3e+1983&relpos=30&citeCnt=3&searchTerm=)

[Li, Z.](https://www-scopus-com.ezproxy.lib.swin.edu.au/authid/detail.uri?origin=resultslist&authorId=56915035200&zone=), [Chen, C.](https://www-scopus-com.ezproxy.lib.swin.edu.au/authid/detail.uri?origin=resultslist&authorId=56245424600&zone=), [Wu, Q.](https://www-scopus-com.ezproxy.lib.swin.edu.au/authid/detail.uri?origin=resultslist&authorId=56272684000&zone=), (...), [Prevedouros, P.D.](https://www-scopus-com.ezproxy.lib.swin.edu.au/authid/detail.uri?origin=resultslist&authorId=35606426100&zone=), [Ma, D.T.](https://www-scopus-com.ezproxy.lib.swin.edu.au/authid/detail.uri?origin=resultslist&authorId=56857830400&zone=) 2018. [Analytic Methods in Accident Research](https://www-scopus-com.ezproxy.lib.swin.edu.au/sourceid/21100261712?origin=resultslist), 20, pp. 1-14

# [An analysis of the characteristics of road traffic injuries and a prediction of fatalities in China from 1996 to 2015](https://www-scopus-com.ezproxy.lib.swin.edu.au/record/display.uri?eid=2-s2.0-85054530389&origin=resultslist&sort=plf-f&src=s&st1=%28predict*+OR+model%29+AND+%28%22serious+crash*%22+OR+%22serious+accident%22+OR+%22serious+collision%22+OR+%22fatal+accident%22+OR+%22fatal+crash%22+OR+%22fatal+collision%22+OR+%22road+deaths%22+OR+%22road+fatal*%22+OR+%22traffic+fatal*%22+OR+%22collision+fatal*%22+OR+%22accident+fatal*%22%29+AND+%28driver%29+&nlo=&nlr=&nls=&sid=34fc91d90b0a1eb0b79c181c6b17236b&sot=b&sdt=b&sl=290&s=TITLE-ABS-KEY%28%28predict*+OR+model%29+AND+%28%22serious+crash*%22+OR+%22serious+accident%22+OR+%22serious+collision%22+OR+%22fatal+accident%22+OR+%22fatal+crash%22+OR+%22fatal+collision%22+OR+%22road+deaths%22+OR+%22road+fatal*%22+OR+%22traffic+fatal*%22+OR+%22collision+fatal*%22+OR+%22accident+fatal*%22%29+AND+%28driver%29+%29+AND+PUBYEAR+%3e+1983&relpos=37&citeCnt=1&searchTerm=)

[Wang, L.](https://www-scopus-com.ezproxy.lib.swin.edu.au/authid/detail.uri?origin=resultslist&authorId=36660147900&zone=), [Yu, C.](https://www-scopus-com.ezproxy.lib.swin.edu.au/authid/detail.uri?origin=resultslist&authorId=57201889726&zone=), [Zhang, Y.](https://www-scopus-com.ezproxy.lib.swin.edu.au/authid/detail.uri?origin=resultslist&authorId=56568510200&zone=), [Luo, L.](https://www-scopus-com.ezproxy.lib.swin.edu.au/authid/detail.uri?origin=resultslist&authorId=57192420067&zone=), [Zhang, G.](https://www-scopus-com.ezproxy.lib.swin.edu.au/authid/detail.uri?origin=resultslist&authorId=57194898786&zone=) 2018. [Traffic Injury Prevention](https://www-scopus-com.ezproxy.lib.swin.edu.au/sourceid/22112?origin=resultslist). 19(7), pp. 749-754

# [The effects of medical marijuana laws on cannabis-involved driving](https://www-scopus-com.ezproxy.lib.swin.edu.au/record/display.uri?eid=2-s2.0-85048172785&origin=resultslist&sort=plf-f&src=s&st1=%28predict*+OR+model%29+AND+%28%22serious+crash*%22+OR+%22serious+accident%22+OR+%22serious+collision%22+OR+%22fatal+accident%22+OR+%22fatal+crash%22+OR+%22fatal+collision%22+OR+%22road+deaths%22+OR+%22road+fatal*%22+OR+%22traffic+fatal*%22+OR+%22collision+fatal*%22+OR+%22accident+fatal*%22%29+AND+%28driver%29+&nlo=&nlr=&nls=&sid=34fc91d90b0a1eb0b79c181c6b17236b&sot=b&sdt=b&sl=290&s=TITLE-ABS-KEY%28%28predict*+OR+model%29+AND+%28%22serious+crash*%22+OR+%22serious+accident%22+OR+%22serious+collision%22+OR+%22fatal+accident%22+OR+%22fatal+crash%22+OR+%22fatal+collision%22+OR+%22road+deaths%22+OR+%22road+fatal*%22+OR+%22traffic+fatal*%22+OR+%22collision+fatal*%22+OR+%22accident+fatal*%22%29+AND+%28driver%29+%29+AND+PUBYEAR+%3e+1983&relpos=40&citeCnt=3&searchTerm=)

[Sevigny, E.L.](https://www-scopus-com.ezproxy.lib.swin.edu.au/authid/detail.uri?origin=resultslist&authorId=24400169900&zone=) 2018. [Accident Analysis and Prevention](https://www-scopus-com.ezproxy.lib.swin.edu.au/sourceid/19532?origin=resultslist). 118, pp. 57-65

# [Exploring traffic safety culture and drunk driving: An examination of the community and DUI related fatal crashes in the U.S. (1993–2015)](https://www-scopus-com.ezproxy.lib.swin.edu.au/record/display.uri?eid=2-s2.0-85047648845&origin=resultslist&sort=plf-f&src=s&st1=%28predict*+OR+model%29+AND+%28%22serious+crash*%22+OR+%22serious+accident%22+OR+%22serious+collision%22+OR+%22fatal+accident%22+OR+%22fatal+crash%22+OR+%22fatal+collision%22+OR+%22road+deaths%22+OR+%22road+fatal*%22+OR+%22traffic+fatal*%22+OR+%22collision+fatal*%22+OR+%22accident+fatal*%22%29+AND+%28driver%29+&nlo=&nlr=&nls=&sid=34fc91d90b0a1eb0b79c181c6b17236b&sot=b&sdt=b&sl=290&s=TITLE-ABS-KEY%28%28predict*+OR+model%29+AND+%28%22serious+crash*%22+OR+%22serious+accident%22+OR+%22serious+collision%22+OR+%22fatal+accident%22+OR+%22fatal+crash%22+OR+%22fatal+collision%22+OR+%22road+deaths%22+OR+%22road+fatal*%22+OR+%22traffic+fatal*%22+OR+%22collision+fatal*%22+OR+%22accident+fatal*%22%29+AND+%28driver%29+%29+AND+PUBYEAR+%3e+1983&relpos=44&citeCnt=1&searchTerm=)

[Stringer, R.J.](https://www-scopus-com.ezproxy.lib.swin.edu.au/authid/detail.uri?origin=resultslist&authorId=57190745354&zone=) 2018. [Transportation Research Part F: Traffic Psychology and Behaviour](https://www-scopus-com.ezproxy.lib.swin.edu.au/sourceid/20897?origin=resultslist), 56, pp. 371-380

# [An investigation on fatality of drivers in vehicle–fixed object accidents on expressways in China: Using multinomial logistic regression model](https://www-scopus-com.ezproxy.lib.swin.edu.au/record/display.uri?eid=2-s2.0-85049039949&origin=resultslist&sort=plf-f&src=s&st1=%28predict*+OR+model%29+AND+%28%22serious+crash*%22+OR+%22serious+accident%22+OR+%22serious+collision%22+OR+%22fatal+accident%22+OR+%22fatal+crash%22+OR+%22fatal+collision%22+OR+%22road+deaths%22+OR+%22road+fatal*%22+OR+%22traffic+fatal*%22+OR+%22collision+fatal*%22+OR+%22accident+fatal*%22%29+AND+%28driver%29+&nlo=&nlr=&nls=&sid=34fc91d90b0a1eb0b79c181c6b17236b&sot=b&sdt=b&sl=290&s=TITLE-ABS-KEY%28%28predict*+OR+model%29+AND+%28%22serious+crash*%22+OR+%22serious+accident%22+OR+%22serious+collision%22+OR+%22fatal+accident%22+OR+%22fatal+crash%22+OR+%22fatal+collision%22+OR+%22road+deaths%22+OR+%22road+fatal*%22+OR+%22traffic+fatal*%22+OR+%22collision+fatal*%22+OR+%22accident+fatal*%22%29+AND+%28driver%29+%29+AND+PUBYEAR+%3e+1983&relpos=45&citeCnt=0&searchTerm=)

[Peng, Y.](https://www-scopus-com.ezproxy.lib.swin.edu.au/authid/detail.uri?origin=resultslist&authorId=57206635106&zone=), [Peng, S.](https://www-scopus-com.ezproxy.lib.swin.edu.au/authid/detail.uri?origin=resultslist&authorId=57195506426&zone=), [Wang, X.](https://www-scopus-com.ezproxy.lib.swin.edu.au/authid/detail.uri?origin=resultslist&authorId=56159285900&zone=), [Tan, S.](https://www-scopus-com.ezproxy.lib.swin.edu.au/authid/detail.uri?origin=resultslist&authorId=57202684678&zone=) 2018. [Proceedings of the Institution of Mechanical Engineers, Part H: Journal of Engineering in Medicine](https://www-scopus-com.ezproxy.lib.swin.edu.au/sourceid/16334?origin=resultslist). 232(7), pp. 643-654

# [Wrong-way driving crashes: A multiple correspondence approach to identify contributing factors](https://www-scopus-com.ezproxy.lib.swin.edu.au/record/display.uri?eid=2-s2.0-85028529118&origin=resultslist&sort=plf-f&src=s&st1=%28predict*+OR+model%29+AND+%28%22serious+crash*%22+OR+%22serious+accident%22+OR+%22serious+collision%22+OR+%22fatal+accident%22+OR+%22fatal+crash%22+OR+%22fatal+collision%22+OR+%22road+deaths%22+OR+%22road+fatal*%22+OR+%22traffic+fatal*%22+OR+%22collision+fatal*%22+OR+%22accident+fatal*%22%29+AND+%28driver%29+&nlo=&nlr=&nls=&sid=34fc91d90b0a1eb0b79c181c6b17236b&sot=b&sdt=b&sl=290&s=TITLE-ABS-KEY%28%28predict*+OR+model%29+AND+%28%22serious+crash*%22+OR+%22serious+accident%22+OR+%22serious+collision%22+OR+%22fatal+accident%22+OR+%22fatal+crash%22+OR+%22fatal+collision%22+OR+%22road+deaths%22+OR+%22road+fatal*%22+OR+%22traffic+fatal*%22+OR+%22collision+fatal*%22+OR+%22accident+fatal*%22%29+AND+%28driver%29+%29+AND+PUBYEAR+%3e+1983&relpos=54&citeCnt=5&searchTerm=)

[Jalayer, M.](https://www-scopus-com.ezproxy.lib.swin.edu.au/authid/detail.uri?origin=resultslist&authorId=55940406200&zone=), [Pour-Rouholamin, M.](https://www-scopus-com.ezproxy.lib.swin.edu.au/authid/detail.uri?origin=resultslist&authorId=56544537700&zone=), [Zhou, H.](https://www-scopus-com.ezproxy.lib.swin.edu.au/authid/detail.uri?origin=resultslist&authorId=7404742053&zone=) 2018. [Traffic Injury Prevention](https://www-scopus-com.ezproxy.lib.swin.edu.au/sourceid/22112?origin=resultslist), 19(1), pp. 35-41

# [Cognitive change and driving behavior among older drivers](https://www-scopus-com.ezproxy.lib.swin.edu.au/record/display.uri?eid=2-s2.0-85061182388&origin=resultslist&sort=plf-f&src=s&st1=%28predict*+OR+model%29+AND+%28%22serious+crash*%22+OR+%22serious+accident%22+OR+%22serious+collision%22+OR+%22fatal+accident%22+OR+%22fatal+crash%22+OR+%22fatal+collision%22+OR+%22road+deaths%22+OR+%22road+fatal*%22+OR+%22traffic+fatal*%22+OR+%22collision+fatal*%22+OR+%22accident+fatal*%22%29+AND+%28driver%29+&nlo=&nlr=&nls=&sid=34fc91d90b0a1eb0b79c181c6b17236b&sot=b&sdt=b&sl=290&s=TITLE-ABS-KEY%28%28predict*+OR+model%29+AND+%28%22serious+crash*%22+OR+%22serious+accident%22+OR+%22serious+collision%22+OR+%22fatal+accident%22+OR+%22fatal+crash%22+OR+%22fatal+collision%22+OR+%22road+deaths%22+OR+%22road+fatal*%22+OR+%22traffic+fatal*%22+OR+%22collision+fatal*%22+OR+%22accident+fatal*%22%29+AND+%28driver%29+%29+AND+PUBYEAR+%3e+1983&relpos=62&citeCnt=0&searchTerm=)

[Fraade-Blanar, L.](https://www-scopus-com.ezproxy.lib.swin.edu.au/authid/detail.uri?origin=resultslist&authorId=23102613500&zone=), [Smith, J.P.](https://www-scopus-com.ezproxy.lib.swin.edu.au/authid/detail.uri?origin=resultslist&authorId=35431277400&zone=) 2018. [Transportation Research Record](https://www-scopus-com.ezproxy.lib.swin.edu.au/sourceid/27418?origin=resultslist), 2672(33), pp. 89-100

# [Complex systems modeling for evaluating potential impact of traffic safety policies: a case on drug-involved fatal crashes](https://www-scopus-com.ezproxy.lib.swin.edu.au/record/display.uri?eid=2-s2.0-85050310506&origin=resultslist&sort=plf-f&src=s&st1=%28predict*+OR+model%29+AND+%28%22serious+crash*%22+OR+%22serious+accident%22+OR+%22serious+collision%22+OR+%22fatal+accident%22+OR+%22fatal+crash%22+OR+%22fatal+collision%22+OR+%22road+deaths%22+OR+%22road+fatal*%22+OR+%22traffic+fatal*%22+OR+%22collision+fatal*%22+OR+%22accident+fatal*%22%29+AND+%28driver%29+&nlo=&nlr=&nls=&sid=34fc91d90b0a1eb0b79c181c6b17236b&sot=b&sdt=b&sl=290&s=TITLE-ABS-KEY%28%28predict*+OR+model%29+AND+%28%22serious+crash*%22+OR+%22serious+accident%22+OR+%22serious+collision%22+OR+%22fatal+accident%22+OR+%22fatal+crash%22+OR+%22fatal+collision%22+OR+%22road+deaths%22+OR+%22road+fatal*%22+OR+%22traffic+fatal*%22+OR+%22collision+fatal*%22+OR+%22accident+fatal*%22%29+AND+%28driver%29+%29+AND+PUBYEAR+%3e+1983&relpos=70&citeCnt=0&searchTerm=)

[Araz, O.M.](https://www-scopus-com.ezproxy.lib.swin.edu.au/authid/detail.uri?origin=resultslist&authorId=26031215900&zone=), [Wilson, F.A.](https://www-scopus-com.ezproxy.lib.swin.edu.au/authid/detail.uri?origin=resultslist&authorId=55648733100&zone=), [Stimpson, J.P.](https://www-scopus-com.ezproxy.lib.swin.edu.au/authid/detail.uri?origin=resultslist&authorId=12771406500&zone=) 2018, [Annals of Operations Research](https://www-scopus-com.ezproxy.lib.swin.edu.au/sourceid/23090?origin=resultslist)

# [Impact of a new law to reduce the legal blood alcohol concentration limit– a poisson regression analysis and descriptive approach](https://www-scopus-com.ezproxy.lib.swin.edu.au/record/display.uri?eid=2-s2.0-85018756611&origin=resultslist&sort=plf-f&src=s&st1=%28predict*+OR+model%29+AND+%28%22serious+crash*%22+OR+%22serious+accident%22+OR+%22serious+collision%22+OR+%22fatal+accident%22+OR+%22fatal+crash%22+OR+%22fatal+collision%22+OR+%22road+deaths%22+OR+%22road+fatal*%22+OR+%22traffic+fatal*%22+OR+%22collision+fatal*%22+OR+%22accident+fatal*%22%29+AND+%28driver%29+&nlo=&nlr=&nls=&sid=34fc91d90b0a1eb0b79c181c6b17236b&sot=b&sdt=b&sl=290&s=TITLE-ABS-KEY%28%28predict*+OR+model%29+AND+%28%22serious+crash*%22+OR+%22serious+accident%22+OR+%22serious+collision%22+OR+%22fatal+accident%22+OR+%22fatal+crash%22+OR+%22fatal+collision%22+OR+%22road+deaths%22+OR+%22road+fatal*%22+OR+%22traffic+fatal*%22+OR+%22collision+fatal*%22+OR+%22accident+fatal*%22%29+AND+%28driver%29+%29+AND+PUBYEAR+%3e+1983&relpos=76&citeCnt=1&searchTerm=)

[Nistal-Nuño, B.](https://www-scopus-com.ezproxy.lib.swin.edu.au/authid/detail.uri?origin=resultslist&authorId=56439995300&zone=) 2017, [Journal of Research in Health Sciences](https://www-scopus-com.ezproxy.lib.swin.edu.au/sourceid/19700175007?origin=resultslist), 17(1),e00374

# [Road traffic collisions in Malawi: Trends and patterns of mortality on scene](https://www-scopus-com.ezproxy.lib.swin.edu.au/record/display.uri?eid=2-s2.0-85041556457&origin=resultslist&sort=plf-f&src=s&st1=%28predict*+OR+model%29+AND+%28%22serious+crash*%22+OR+%22serious+accident%22+OR+%22serious+collision%22+OR+%22fatal+accident%22+OR+%22fatal+crash%22+OR+%22fatal+collision%22+OR+%22road+deaths%22+OR+%22road+fatal*%22+OR+%22traffic+fatal*%22+OR+%22collision+fatal*%22+OR+%22accident+fatal*%22%29+AND+%28driver%29+&nlo=&nlr=&nls=&sid=34fc91d90b0a1eb0b79c181c6b17236b&sot=b&sdt=b&sl=290&s=TITLE-ABS-KEY%28%28predict*+OR+model%29+AND+%28%22serious+crash*%22+OR+%22serious+accident%22+OR+%22serious+collision%22+OR+%22fatal+accident%22+OR+%22fatal+crash%22+OR+%22fatal+collision%22+OR+%22road+deaths%22+OR+%22road+fatal*%22+OR+%22traffic+fatal*%22+OR+%22collision+fatal*%22+OR+%22accident+fatal*%22%29+AND+%28driver%29+%29+AND+PUBYEAR+%3e+1983&relpos=77&citeCnt=1&searchTerm=)

[Schlottmann, F.](https://www-scopus-com.ezproxy.lib.swin.edu.au/authid/detail.uri?origin=resultslist&authorId=56609168000&zone=), [Tyson, A.F.](https://www-scopus-com.ezproxy.lib.swin.edu.au/authid/detail.uri?origin=resultslist&authorId=55953707400&zone=), [Cairns, B.A.](https://www-scopus-com.ezproxy.lib.swin.edu.au/authid/detail.uri?origin=resultslist&authorId=7004508743&zone=), [Varela, C.](https://www-scopus-com.ezproxy.lib.swin.edu.au/authid/detail.uri?origin=resultslist&authorId=55935993900&zone=), [Charles, A.G.](https://www-scopus-com.ezproxy.lib.swin.edu.au/authid/detail.uri?origin=resultslist&authorId=23471869800&zone=) 2017, [Malawi Medical Journal](https://www-scopus-com.ezproxy.lib.swin.edu.au/sourceid/59557?origin=resultslist), 29(4), pp. 301-305

# [The role of restraint omission in alcohol-related traffic fatalities](https://www-scopus-com.ezproxy.lib.swin.edu.au/record/display.uri?eid=2-s2.0-85032468423&origin=resultslist&sort=plf-f&src=s&st1=%28predict*+OR+model%29+AND+%28%22serious+crash*%22+OR+%22serious+accident%22+OR+%22serious+collision%22+OR+%22fatal+accident%22+OR+%22fatal+crash%22+OR+%22fatal+collision%22+OR+%22road+deaths%22+OR+%22road+fatal*%22+OR+%22traffic+fatal*%22+OR+%22collision+fatal*%22+OR+%22accident+fatal*%22%29+AND+%28driver%29+&nlo=&nlr=&nls=&sid=34fc91d90b0a1eb0b79c181c6b17236b&sot=b&sdt=b&sl=290&s=TITLE-ABS-KEY%28%28predict*+OR+model%29+AND+%28%22serious+crash*%22+OR+%22serious+accident%22+OR+%22serious+collision%22+OR+%22fatal+accident%22+OR+%22fatal+crash%22+OR+%22fatal+collision%22+OR+%22road+deaths%22+OR+%22road+fatal*%22+OR+%22traffic+fatal*%22+OR+%22collision+fatal*%22+OR+%22accident+fatal*%22%29+AND+%28driver%29+%29+AND+PUBYEAR+%3e+1983&relpos=79&citeCnt=0&searchTerm=)

[Evangelidis, I.](https://www-scopus-com.ezproxy.lib.swin.edu.au/authid/detail.uri?origin=resultslist&authorId=55924756300&zone=) 2017, [Drug and Alcohol Dependence](https://www-scopus-com.ezproxy.lib.swin.edu.au/sourceid/26380?origin=resultslist), 180, pp. 423-426

# [Cannabis and crash responsibility while driving below the alcohol per se legal limit](https://www-scopus-com.ezproxy.lib.swin.edu.au/record/display.uri?eid=2-s2.0-85027857776&origin=resultslist&sort=plf-f&src=s&st1=%28predict*+OR+model%29+AND+%28%22serious+crash*%22+OR+%22serious+accident%22+OR+%22serious+collision%22+OR+%22fatal+accident%22+OR+%22fatal+crash%22+OR+%22fatal+collision%22+OR+%22road+deaths%22+OR+%22road+fatal*%22+OR+%22traffic+fatal*%22+OR+%22collision+fatal*%22+OR+%22accident+fatal*%22%29+AND+%28driver%29+&nlo=&nlr=&nls=&sid=34fc91d90b0a1eb0b79c181c6b17236b&sot=b&sdt=b&sl=290&s=TITLE-ABS-KEY%28%28predict*+OR+model%29+AND+%28%22serious+crash*%22+OR+%22serious+accident%22+OR+%22serious+collision%22+OR+%22fatal+accident%22+OR+%22fatal+crash%22+OR+%22fatal+collision%22+OR+%22road+deaths%22+OR+%22road+fatal*%22+OR+%22traffic+fatal*%22+OR+%22collision+fatal*%22+OR+%22accident+fatal*%22%29+AND+%28driver%29+%29+AND+PUBYEAR+%3e+1983&relpos=80&citeCnt=3&searchTerm=)

[Riaz, S.](https://www-scopus-com.ezproxy.lib.swin.edu.au/authid/detail.uri?origin=resultslist&authorId=36683210100&zone=), [Khan, L.](https://www-scopus-com.ezproxy.lib.swin.edu.au/authid/detail.uri?origin=resultslist&authorId=15019384600&zone=) 2017, [Journal of the Brazilian Society of Mechanical Sciences and Engineering](https://www-scopus-com.ezproxy.lib.swin.edu.au/sourceid/71359?origin=resultslist), 39(11), pp. 4305-4333

# [Automobile-dependency as a barrier to vision zero, evidence from the states in the USA](https://www-scopus-com.ezproxy.lib.swin.edu.au/record/display.uri?eid=2-s2.0-85029176330&origin=resultslist&sort=plf-f&src=s&st1=%28predict*+OR+model%29+AND+%28%22serious+crash*%22+OR+%22serious+accident%22+OR+%22serious+collision%22+OR+%22fatal+accident%22+OR+%22fatal+crash%22+OR+%22fatal+collision%22+OR+%22road+deaths%22+OR+%22road+fatal*%22+OR+%22traffic+fatal*%22+OR+%22collision+fatal*%22+OR+%22accident+fatal*%22%29+AND+%28driver%29+&nlo=&nlr=&nls=&sid=34fc91d90b0a1eb0b79c181c6b17236b&sot=b&sdt=b&sl=290&s=TITLE-ABS-KEY%28%28predict*+OR+model%29+AND+%28%22serious+crash*%22+OR+%22serious+accident%22+OR+%22serious+collision%22+OR+%22fatal+accident%22+OR+%22fatal+crash%22+OR+%22fatal+collision%22+OR+%22road+deaths%22+OR+%22road+fatal*%22+OR+%22traffic+fatal*%22+OR+%22collision+fatal*%22+OR+%22accident+fatal*%22%29+AND+%28driver%29+%29+AND+PUBYEAR+%3e+1983&relpos=83&citeCnt=5&searchTerm=)

[Ahangari, H.](https://www-scopus-com.ezproxy.lib.swin.edu.au/authid/detail.uri?origin=resultslist&authorId=55619124800&zone=), [Atkinson-Palombo, C.](https://www-scopus-com.ezproxy.lib.swin.edu.au/authid/detail.uri?origin=resultslist&authorId=15043762300&zone=), [Garrick, N.W.](https://www-scopus-com.ezproxy.lib.swin.edu.au/authid/detail.uri?origin=resultslist&authorId=7004149968&zone=) 2017. [Accident Analysis and Prevention](https://www-scopus-com.ezproxy.lib.swin.edu.au/sourceid/19532?origin=resultslist), 107, pp. 77-85

# [Economic and statistical perspectives on traffic safety in Louisiana, 2005–2015](https://www-scopus-com.ezproxy.lib.swin.edu.au/record/display.uri?eid=2-s2.0-85019970102&origin=resultslist&sort=plf-f&src=s&st1=%28predict*+OR+model%29+AND+%28%22serious+crash*%22+OR+%22serious+accident%22+OR+%22serious+collision%22+OR+%22fatal+accident%22+OR+%22fatal+crash%22+OR+%22fatal+collision%22+OR+%22road+deaths%22+OR+%22road+fatal*%22+OR+%22traffic+fatal*%22+OR+%22collision+fatal*%22+OR+%22accident+fatal*%22%29+AND+%28driver%29+&nlo=&nlr=&nls=&sid=34fc91d90b0a1eb0b79c181c6b17236b&sot=b&sdt=b&sl=290&s=TITLE-ABS-KEY%28%28predict*+OR+model%29+AND+%28%22serious+crash*%22+OR+%22serious+accident%22+OR+%22serious+collision%22+OR+%22fatal+accident%22+OR+%22fatal+crash%22+OR+%22fatal+collision%22+OR+%22road+deaths%22+OR+%22road+fatal*%22+OR+%22traffic+fatal*%22+OR+%22collision+fatal*%22+OR+%22accident+fatal*%22%29+AND+%28driver%29+%29+AND+PUBYEAR+%3e+1983&relpos=86&citeCnt=0&searchTerm=)

[Lukongo, O.E.B.](https://www-scopus-com.ezproxy.lib.swin.edu.au/authid/detail.uri?origin=resultslist&authorId=57194410152&zone=) 2017, [Journal of Safety Research](https://www-scopus-com.ezproxy.lib.swin.edu.au/sourceid/29284?origin=resultslist), 62, pp. 43-51

# [What factors impact injury severity of vehicle to electric bike crashes in China?](https://www-scopus-com.ezproxy.lib.swin.edu.au/record/display.uri?eid=2-s2.0-85028355692&origin=resultslist&sort=plf-f&src=s&st1=%28predict*+OR+model%29+AND+%28%22serious+crash*%22+OR+%22serious+accident%22+OR+%22serious+collision%22+OR+%22fatal+accident%22+OR+%22fatal+crash%22+OR+%22fatal+collision%22+OR+%22road+deaths%22+OR+%22road+fatal*%22+OR+%22traffic+fatal*%22+OR+%22collision+fatal*%22+OR+%22accident+fatal*%22%29+AND+%28driver%29+&nlo=&nlr=&nls=&sid=34fc91d90b0a1eb0b79c181c6b17236b&sot=b&sdt=b&sl=290&s=TITLE-ABS-KEY%28%28predict*+OR+model%29+AND+%28%22serious+crash*%22+OR+%22serious+accident%22+OR+%22serious+collision%22+OR+%22fatal+accident%22+OR+%22fatal+crash%22+OR+%22fatal+collision%22+OR+%22road+deaths%22+OR+%22road+fatal*%22+OR+%22traffic+fatal*%22+OR+%22collision+fatal*%22+OR+%22accident+fatal*%22%29+AND+%28driver%29+%29+AND+PUBYEAR+%3e+1983&relpos=88&citeCnt=1&searchTerm=)

[Yuan, Q.](https://www-scopus-com.ezproxy.lib.swin.edu.au/authid/detail.uri?origin=resultslist&authorId=57201300648&zone=), [Yang, H.](https://www-scopus-com.ezproxy.lib.swin.edu.au/authid/detail.uri?origin=resultslist&authorId=57195470044&zone=), [Huang, J.](https://www-scopus-com.ezproxy.lib.swin.edu.au/authid/detail.uri?origin=resultslist&authorId=57195468681&zone=), (...), [Li, Y.](https://www-scopus-com.ezproxy.lib.swin.edu.au/authid/detail.uri?origin=resultslist&authorId=56372976400&zone=), [Theofilatos, A.](https://www-scopus-com.ezproxy.lib.swin.edu.au/authid/detail.uri?origin=resultslist&authorId=55352104000&zone=) 2017, [Advances in Mechanical Engineering](https://www-scopus-com.ezproxy.lib.swin.edu.au/sourceid/19700182334?origin=resultslist), 9(8), pp. 1-10

# [Analysis of road traffic fatal accidents using data mining techniques](https://www-scopus-com.ezproxy.lib.swin.edu.au/record/display.uri?eid=2-s2.0-85026643673&origin=resultslist&sort=plf-f&src=s&st1=%28predict*+OR+model%29+AND+%28%22serious+crash*%22+OR+%22serious+accident%22+OR+%22serious+collision%22+OR+%22fatal+accident%22+OR+%22fatal+crash%22+OR+%22fatal+collision%22+OR+%22road+deaths%22+OR+%22road+fatal*%22+OR+%22traffic+fatal*%22+OR+%22collision+fatal*%22+OR+%22accident+fatal*%22%29+AND+%28driver%29+&nlo=&nlr=&nls=&sid=34fc91d90b0a1eb0b79c181c6b17236b&sot=b&sdt=b&sl=290&s=TITLE-ABS-KEY%28%28predict*+OR+model%29+AND+%28%22serious+crash*%22+OR+%22serious+accident%22+OR+%22serious+collision%22+OR+%22fatal+accident%22+OR+%22fatal+crash%22+OR+%22fatal+collision%22+OR+%22road+deaths%22+OR+%22road+fatal*%22+OR+%22traffic+fatal*%22+OR+%22collision+fatal*%22+OR+%22accident+fatal*%22%29+AND+%28driver%29+%29+AND+PUBYEAR+%3e+1983&relpos=91&citeCnt=12&searchTerm=)

[Li, L.](https://www-scopus-com.ezproxy.lib.swin.edu.au/authid/detail.uri?origin=resultslist&authorId=57195261030&zone=), [Shrestha, S.](https://www-scopus-com.ezproxy.lib.swin.edu.au/authid/detail.uri?origin=resultslist&authorId=57194002271&zone=), [Hu, G.](https://www-scopus-com.ezproxy.lib.swin.edu.au/authid/detail.uri?origin=resultslist&authorId=7401490166&zone=) 2017, Proceedings - 2017 15th IEEE/ACIS International Conference on Software Engineering Research, Management and Applications, SERA 2017, 7965753, pp. 363-370

# [Alcohol-control public service announcements (PSAs) and drunk-driving fatal accidents in the United States, 1996–2010](https://www-scopus-com.ezproxy.lib.swin.edu.au/record/display.uri?eid=2-s2.0-85016612304&origin=resultslist&sort=plf-f&src=s&st1=%28predict*+OR+model%29+AND+%28%22serious+crash*%22+OR+%22serious+accident%22+OR+%22serious+collision%22+OR+%22fatal+accident%22+OR+%22fatal+crash%22+OR+%22fatal+collision%22+OR+%22road+deaths%22+OR+%22road+fatal*%22+OR+%22traffic+fatal*%22+OR+%22collision+fatal*%22+OR+%22accident+fatal*%22%29+AND+%28driver%29+&nlo=&nlr=&nls=&sid=34fc91d90b0a1eb0b79c181c6b17236b&sot=b&sdt=b&sl=290&s=TITLE-ABS-KEY%28%28predict*+OR+model%29+AND+%28%22serious+crash*%22+OR+%22serious+accident%22+OR+%22serious+collision%22+OR+%22fatal+accident%22+OR+%22fatal+crash%22+OR+%22fatal+collision%22+OR+%22road+deaths%22+OR+%22road+fatal*%22+OR+%22traffic+fatal*%22+OR+%22collision+fatal*%22+OR+%22accident+fatal*%22%29+AND+%28driver%29+%29+AND+PUBYEAR+%3e+1983&relpos=93&citeCnt=4&searchTerm=)

[Niederdeppe, J.](https://www-scopus-com.ezproxy.lib.swin.edu.au/authid/detail.uri?origin=resultslist&authorId=16029149900&zone=), [Avery, R.](https://www-scopus-com.ezproxy.lib.swin.edu.au/authid/detail.uri?origin=resultslist&authorId=7102735341&zone=), [Miller, E.N.](https://www-scopus-com.ezproxy.lib.swin.edu.au/authid/detail.uri?origin=resultslist&authorId=57193796883&zone=) 2017, [Preventive Medicine](https://www-scopus-com.ezproxy.lib.swin.edu.au/sourceid/17665?origin=resultslist), 99, pp. 320-325

# [Relationship of traffic fatality rates to maximum state speed limits](https://www-scopus-com.ezproxy.lib.swin.edu.au/record/display.uri?eid=2-s2.0-85006129704&origin=resultslist&sort=plf-f&src=s&st1=%28predict*+OR+model%29+AND+%28%22serious+crash*%22+OR+%22serious+accident%22+OR+%22serious+collision%22+OR+%22fatal+accident%22+OR+%22fatal+crash%22+OR+%22fatal+collision%22+OR+%22road+deaths%22+OR+%22road+fatal*%22+OR+%22traffic+fatal*%22+OR+%22collision+fatal*%22+OR+%22accident+fatal*%22%29+AND+%28driver%29+&nlo=&nlr=&nls=&sid=34fc91d90b0a1eb0b79c181c6b17236b&sot=b&sdt=b&sl=290&s=TITLE-ABS-KEY%28%28predict*+OR+model%29+AND+%28%22serious+crash*%22+OR+%22serious+accident%22+OR+%22serious+collision%22+OR+%22fatal+accident%22+OR+%22fatal+crash%22+OR+%22fatal+collision%22+OR+%22road+deaths%22+OR+%22road+fatal*%22+OR+%22traffic+fatal*%22+OR+%22collision+fatal*%22+OR+%22accident+fatal*%22%29+AND+%28driver%29+%29+AND+PUBYEAR+%3e+1983&relpos=96&citeCnt=4&searchTerm=)

[Farmer, C.M.](https://www-scopus-com.ezproxy.lib.swin.edu.au/authid/detail.uri?origin=resultslist&authorId=7101618669&zone=) 2017, [Traffic Injury Prevention](https://www-scopus-com.ezproxy.lib.swin.edu.au/sourceid/22112?origin=resultslist), 18(4), pp. 375-380

# [How did the economic recession (2008–2010) influence traffic fatalities in OECD-countries?](https://www-scopus-com.ezproxy.lib.swin.edu.au/record/display.uri?eid=2-s2.0-85014280963&origin=resultslist&sort=plf-f&src=s&st1=%28predict*+OR+model%29+AND+%28%22serious+crash*%22+OR+%22serious+accident%22+OR+%22serious+collision%22+OR+%22fatal+accident%22+OR+%22fatal+crash%22+OR+%22fatal+collision%22+OR+%22road+deaths%22+OR+%22road+fatal*%22+OR+%22traffic+fatal*%22+OR+%22collision+fatal*%22+OR+%22accident+fatal*%22%29+AND+%28driver%29+&nlo=&nlr=&nls=&sid=34fc91d90b0a1eb0b79c181c6b17236b&sot=b&sdt=b&sl=290&s=TITLE-ABS-KEY%28%28predict*+OR+model%29+AND+%28%22serious+crash*%22+OR+%22serious+accident%22+OR+%22serious+collision%22+OR+%22fatal+accident%22+OR+%22fatal+crash%22+OR+%22fatal+collision%22+OR+%22road+deaths%22+OR+%22road+fatal*%22+OR+%22traffic+fatal*%22+OR+%22collision+fatal*%22+OR+%22accident+fatal*%22%29+AND+%28driver%29+%29+AND+PUBYEAR+%3e+1983&relpos=99&citeCnt=5&searchTerm=)

[Wegman, F.](https://www-scopus-com.ezproxy.lib.swin.edu.au/authid/detail.uri?origin=resultslist&authorId=6507786058&zone=), [Allsop, R.](https://www-scopus-com.ezproxy.lib.swin.edu.au/authid/detail.uri?origin=resultslist&authorId=6701702105&zone=), [Antoniou, C.](https://www-scopus-com.ezproxy.lib.swin.edu.au/authid/detail.uri?origin=resultslist&authorId=7004031914&zone=), (...), [Lloyd, D.](https://www-scopus-com.ezproxy.lib.swin.edu.au/authid/detail.uri?origin=resultslist&authorId=57193492697&zone=), [Wijnen, W.](https://www-scopus-com.ezproxy.lib.swin.edu.au/authid/detail.uri?origin=resultslist&authorId=30268052600&zone=) 2017, [Accident Analysis and Prevention](https://www-scopus-com.ezproxy.lib.swin.edu.au/sourceid/19532?origin=resultslist), 102, pp. 51-59

# [Role of alcohol and marijuana use in the initiation of fatal two-vehicle crashes](https://www-scopus-com.ezproxy.lib.swin.edu.au/record/display.uri?eid=2-s2.0-85020188420&origin=resultslist&sort=plf-f&src=s&st1=%28predict*+OR+model%29+AND+%28%22serious+crash*%22+OR+%22serious+accident%22+OR+%22serious+collision%22+OR+%22fatal+accident%22+OR+%22fatal+crash%22+OR+%22fatal+collision%22+OR+%22road+deaths%22+OR+%22road+fatal*%22+OR+%22traffic+fatal*%22+OR+%22collision+fatal*%22+OR+%22accident+fatal*%22%29+AND+%28driver%29+&nlo=&nlr=&nls=&sid=34fc91d90b0a1eb0b79c181c6b17236b&sot=b&sdt=b&sl=290&s=TITLE-ABS-KEY%28%28predict*+OR+model%29+AND+%28%22serious+crash*%22+OR+%22serious+accident%22+OR+%22serious+collision%22+OR+%22fatal+accident%22+OR+%22fatal+crash%22+OR+%22fatal+collision%22+OR+%22road+deaths%22+OR+%22road+fatal*%22+OR+%22traffic+fatal*%22+OR+%22collision+fatal*%22+OR+%22accident+fatal*%22%29+AND+%28driver%29+%29+AND+PUBYEAR+%3e+1983&relpos=101&citeCnt=4&searchTerm=)

[Li, G.](https://www-scopus-com.ezproxy.lib.swin.edu.au/authid/detail.uri?origin=resultslist&authorId=55547060800&zone=), [Chihuri, S.](https://www-scopus-com.ezproxy.lib.swin.edu.au/authid/detail.uri?origin=resultslist&authorId=57063490100&zone=), [Brady, J.E.](https://www-scopus-com.ezproxy.lib.swin.edu.au/authid/detail.uri?origin=resultslist&authorId=15828779600&zone=) 2017, [Annals of Epidemiology](https://www-scopus-com.ezproxy.lib.swin.edu.au/sourceid/19569?origin=resultslist), 27(5), pp. 342-347.e1

# [Exploring the effects of state highway safety laws and sociocultural characteristics on fatal crashes](https://www-scopus-com.ezproxy.lib.swin.edu.au/record/display.uri?eid=2-s2.0-84988959011&origin=resultslist&sort=plf-f&src=s&st1=%28predict*+OR+model%29+AND+%28%22serious+crash*%22+OR+%22serious+accident%22+OR+%22serious+collision%22+OR+%22fatal+accident%22+OR+%22fatal+crash%22+OR+%22fatal+collision%22+OR+%22road+deaths%22+OR+%22road+fatal*%22+OR+%22traffic+fatal*%22+OR+%22collision+fatal*%22+OR+%22accident+fatal*%22%29+AND+%28driver%29+&nlo=&nlr=&nls=&sid=34fc91d90b0a1eb0b79c181c6b17236b&sot=b&sdt=b&sl=290&s=TITLE-ABS-KEY%28%28predict*+OR+model%29+AND+%28%22serious+crash*%22+OR+%22serious+accident%22+OR+%22serious+collision%22+OR+%22fatal+accident%22+OR+%22fatal+crash%22+OR+%22fatal+collision%22+OR+%22road+deaths%22+OR+%22road+fatal*%22+OR+%22traffic+fatal*%22+OR+%22collision+fatal*%22+OR+%22accident+fatal*%22%29+AND+%28driver%29+%29+AND+PUBYEAR+%3e+1983&relpos=103&citeCnt=2&searchTerm=)

[Dong, C.](https://www-scopus-com.ezproxy.lib.swin.edu.au/authid/detail.uri?origin=resultslist&authorId=25627314300&zone=), [Nambisan, S.S.](https://www-scopus-com.ezproxy.lib.swin.edu.au/authid/detail.uri?origin=resultslist&authorId=6603883263&zone=), [Clarke, D.B.](https://www-scopus-com.ezproxy.lib.swin.edu.au/authid/detail.uri?origin=resultslist&authorId=56421198000&zone=), [Sun, J.](https://www-scopus-com.ezproxy.lib.swin.edu.au/authid/detail.uri?origin=resultslist&authorId=56298538800&zone=) 2017, [Traffic Injury Prevention](https://www-scopus-com.ezproxy.lib.swin.edu.au/sourceid/22112?origin=resultslist), 18(3), pp. 299-305

# [Modeling faults among e-bike-related fatal crashes in China](https://www-scopus-com.ezproxy.lib.swin.edu.au/record/display.uri?eid=2-s2.0-85009998337&origin=resultslist&sort=plf-f&src=s&st1=%28predict*+OR+model%29+AND+%28%22serious+crash*%22+OR+%22serious+accident%22+OR+%22serious+collision%22+OR+%22fatal+accident%22+OR+%22fatal+crash%22+OR+%22fatal+collision%22+OR+%22road+deaths%22+OR+%22road+fatal*%22+OR+%22traffic+fatal*%22+OR+%22collision+fatal*%22+OR+%22accident+fatal*%22%29+AND+%28driver%29+&nlo=&nlr=&nls=&sid=34fc91d90b0a1eb0b79c181c6b17236b&sot=b&sdt=b&sl=290&s=TITLE-ABS-KEY%28%28predict*+OR+model%29+AND+%28%22serious+crash*%22+OR+%22serious+accident%22+OR+%22serious+collision%22+OR+%22fatal+accident%22+OR+%22fatal+crash%22+OR+%22fatal+collision%22+OR+%22road+deaths%22+OR+%22road+fatal*%22+OR+%22traffic+fatal*%22+OR+%22collision+fatal*%22+OR+%22accident+fatal*%22%29+AND+%28driver%29+%29+AND+PUBYEAR+%3e+1983&relpos=107&citeCnt=7&searchTerm=)

[Wang, C.](https://www-scopus-com.ezproxy.lib.swin.edu.au/authid/detail.uri?origin=resultslist&authorId=56014811800&zone=), [Xu, C.](https://www-scopus-com.ezproxy.lib.swin.edu.au/authid/detail.uri?origin=resultslist&authorId=35305963300&zone=), [Xia, J.](https://www-scopus-com.ezproxy.lib.swin.edu.au/authid/detail.uri?origin=resultslist&authorId=57193006448&zone=), [Qian, Z.](https://www-scopus-com.ezproxy.lib.swin.edu.au/authid/detail.uri?origin=resultslist&authorId=7201384729&zone=), 2017, [Traffic Injury Prevention](https://www-scopus-com.ezproxy.lib.swin.edu.au/sourceid/22112?origin=resultslist), 18(2), pp. 175-181

# [Age-related differences in fatal intersection crashes in the United States](https://www-scopus-com.ezproxy.lib.swin.edu.au/record/display.uri?eid=2-s2.0-84995784767&origin=resultslist&sort=plf-f&src=s&st1=%28predict*+OR+model%29+AND+%28%22serious+crash*%22+OR+%22serious+accident%22+OR+%22serious+collision%22+OR+%22fatal+accident%22+OR+%22fatal+crash%22+OR+%22fatal+collision%22+OR+%22road+deaths%22+OR+%22road+fatal*%22+OR+%22traffic+fatal*%22+OR+%22collision+fatal*%22+OR+%22accident+fatal*%22%29+AND+%28driver%29+&nlo=&nlr=&nls=&sid=34fc91d90b0a1eb0b79c181c6b17236b&sot=b&sdt=b&sl=290&s=TITLE-ABS-KEY%28%28predict*+OR+model%29+AND+%28%22serious+crash*%22+OR+%22serious+accident%22+OR+%22serious+collision%22+OR+%22fatal+accident%22+OR+%22fatal+crash%22+OR+%22fatal+collision%22+OR+%22road+deaths%22+OR+%22road+fatal*%22+OR+%22traffic+fatal*%22+OR+%22collision+fatal*%22+OR+%22accident+fatal*%22%29+AND+%28driver%29+%29+AND+PUBYEAR+%3e+1983&relpos=108&citeCnt=9&searchTerm=)

[Lombardi, D.A.](https://www-scopus-com.ezproxy.lib.swin.edu.au/authid/detail.uri?origin=resultslist&authorId=7102003532&zone=), [Horrey, W.J.](https://www-scopus-com.ezproxy.lib.swin.edu.au/authid/detail.uri?origin=resultslist&authorId=6507448827&zone=), [Courtney, T.K.](https://www-scopus-com.ezproxy.lib.swin.edu.au/authid/detail.uri?origin=resultslist&authorId=7005904214&zone=) 2017, [Accident Analysis and Prevention](https://www-scopus-com.ezproxy.lib.swin.edu.au/sourceid/19532?origin=resultslist), 99, pp. 20-29

# [State-level comparison of traffic fatality data in consideration of marijuana laws](https://www-scopus-com.ezproxy.lib.swin.edu.au/record/display.uri?eid=2-s2.0-85033782204&origin=resultslist&sort=plf-f&src=s&st1=%28predict*+OR+model%29+AND+%28%22serious+crash*%22+OR+%22serious+accident%22+OR+%22serious+collision%22+OR+%22fatal+accident%22+OR+%22fatal+crash%22+OR+%22fatal+collision%22+OR+%22road+deaths%22+OR+%22road+fatal*%22+OR+%22traffic+fatal*%22+OR+%22collision+fatal*%22+OR+%22accident+fatal*%22%29+AND+%28driver%29+&nlo=&nlr=&nls=&sid=34fc91d90b0a1eb0b79c181c6b17236b&sot=b&sdt=b&sl=290&s=TITLE-ABS-KEY%28%28predict*+OR+model%29+AND+%28%22serious+crash*%22+OR+%22serious+accident%22+OR+%22serious+collision%22+OR+%22fatal+accident%22+OR+%22fatal+crash%22+OR+%22fatal+collision%22+OR+%22road+deaths%22+OR+%22road+fatal*%22+OR+%22traffic+fatal*%22+OR+%22collision+fatal*%22+OR+%22accident+fatal*%22%29+AND+%28driver%29+%29+AND+PUBYEAR+%3e+1983&relpos=110&citeCnt=1&searchTerm=)

[Hamzeie, R.](https://www-scopus-com.ezproxy.lib.swin.edu.au/authid/detail.uri?origin=resultslist&authorId=57193604872&zone=), [Thompson, I.](https://www-scopus-com.ezproxy.lib.swin.edu.au/authid/detail.uri?origin=resultslist&authorId=57196954203&zone=), [Roy, S.](https://www-scopus-com.ezproxy.lib.swin.edu.au/authid/detail.uri?origin=resultslist&authorId=57196941191&zone=), [Savolainen, P.T.](https://www-scopus-com.ezproxy.lib.swin.edu.au/authid/detail.uri?origin=resultslist&authorId=35410522800&zone=) 2017, [Transportation Research Record](https://www-scopus-com.ezproxy.lib.swin.edu.au/sourceid/27418?origin=resultslist), 2660, pp. 78-85

# [Application of multiple logistic regression, Bayesian logistic and classification tree to identify the significant factors influencing crash severity](https://www-scopus-com.ezproxy.lib.swin.edu.au/record/display.uri?eid=2-s2.0-85034607720&origin=resultslist&sort=plf-f&src=s&st1=%28predict*+OR+model%29+AND+%28%22serious+crash*%22+OR+%22serious+accident%22+OR+%22serious+collision%22+OR+%22fatal+accident%22+OR+%22fatal+crash%22+OR+%22fatal+collision%22+OR+%22road+deaths%22+OR+%22road+fatal*%22+OR+%22traffic+fatal*%22+OR+%22collision+fatal*%22+OR+%22accident+fatal*%22%29+AND+%28driver%29+&nlo=&nlr=&nls=&sid=34fc91d90b0a1eb0b79c181c6b17236b&sot=b&sdt=b&sl=290&s=TITLE-ABS-KEY%28%28predict*+OR+model%29+AND+%28%22serious+crash*%22+OR+%22serious+accident%22+OR+%22serious+collision%22+OR+%22fatal+accident%22+OR+%22fatal+crash%22+OR+%22fatal+collision%22+OR+%22road+deaths%22+OR+%22road+fatal*%22+OR+%22traffic+fatal*%22+OR+%22collision+fatal*%22+OR+%22accident+fatal*%22%29+AND+%28driver%29+%29+AND+PUBYEAR+%3e+1983&relpos=113&citeCnt=0&searchTerm=)

[Tazik, M.](https://www-scopus-com.ezproxy.lib.swin.edu.au/authid/detail.uri?origin=resultslist&authorId=57197754852&zone=), [Aghayan, I.](https://www-scopus-com.ezproxy.lib.swin.edu.au/authid/detail.uri?origin=resultslist&authorId=55209893400&zone=), [Sadeghi, M.](https://www-scopus-com.ezproxy.lib.swin.edu.au/authid/detail.uri?origin=resultslist&authorId=57197754586&zone=) 2017, [Journal of Engineering Science and Technology](https://www-scopus-com.ezproxy.lib.swin.edu.au/sourceid/18200156709?origin=resultslist), 12(11), pp. 3044-3056

# [An investigation of the speeding-related crash designation through crash narrative reviews sampled via logistic regression](https://www-scopus-com.ezproxy.lib.swin.edu.au/record/display.uri?eid=2-s2.0-84991274957&origin=resultslist&sort=plf-f&src=s&st1=%28predict*+OR+model%29+AND+%28%22serious+crash*%22+OR+%22serious+accident%22+OR+%22serious+collision%22+OR+%22fatal+accident%22+OR+%22fatal+crash%22+OR+%22fatal+collision%22+OR+%22road+deaths%22+OR+%22road+fatal*%22+OR+%22traffic+fatal*%22+OR+%22collision+fatal*%22+OR+%22accident+fatal*%22%29+AND+%28driver%29+&nlo=&nlr=&nls=&sid=34fc91d90b0a1eb0b79c181c6b17236b&sot=b&sdt=b&sl=290&s=TITLE-ABS-KEY%28%28predict*+OR+model%29+AND+%28%22serious+crash*%22+OR+%22serious+accident%22+OR+%22serious+collision%22+OR+%22fatal+accident%22+OR+%22fatal+crash%22+OR+%22fatal+collision%22+OR+%22road+deaths%22+OR+%22road+fatal*%22+OR+%22traffic+fatal*%22+OR+%22collision+fatal*%22+OR+%22accident+fatal*%22%29+AND+%28driver%29+%29+AND+PUBYEAR+%3e+1983&relpos=114&citeCnt=6&searchTerm=)

[Fitzpatrick, C.D.](https://www-scopus-com.ezproxy.lib.swin.edu.au/authid/detail.uri?origin=resultslist&authorId=55957185900&zone=), [Rakasi, S.](https://www-scopus-com.ezproxy.lib.swin.edu.au/authid/detail.uri?origin=resultslist&authorId=57191512325&zone=), [Knodler, M.A.](https://www-scopus-com.ezproxy.lib.swin.edu.au/authid/detail.uri?origin=resultslist&authorId=57207533831&zone=) 2017, [Accident Analysis and Prevention](https://www-scopus-com.ezproxy.lib.swin.edu.au/sourceid/19532?origin=resultslist), 98, pp. 57-63.

# [Trends in traffic fatalities in Mexico: examining progress on the decade of action for road safety 2011–2020](https://www-scopus-com.ezproxy.lib.swin.edu.au/record/display.uri?eid=2-s2.0-84979966310&origin=resultslist&sort=plf-f&src=s&st1=%28predict*+OR+model%29+AND+%28%22serious+crash*%22+OR+%22serious+accident%22+OR+%22serious+collision%22+OR+%22fatal+accident%22+OR+%22fatal+crash%22+OR+%22fatal+collision%22+OR+%22road+deaths%22+OR+%22road+fatal*%22+OR+%22traffic+fatal*%22+OR+%22collision+fatal*%22+OR+%22accident+fatal*%22%29+AND+%28driver%29+&nlo=&nlr=&nls=&sid=34fc91d90b0a1eb0b79c181c6b17236b&sot=b&sdt=b&sl=290&s=TITLE-ABS-KEY%28%28predict*+OR+model%29+AND+%28%22serious+crash*%22+OR+%22serious+accident%22+OR+%22serious+collision%22+OR+%22fatal+accident%22+OR+%22fatal+crash%22+OR+%22fatal+collision%22+OR+%22road+deaths%22+OR+%22road+fatal*%22+OR+%22traffic+fatal*%22+OR+%22collision+fatal*%22+OR+%22accident+fatal*%22%29+AND+%28driver%29+%29+AND+PUBYEAR+%3e+1983&relpos=120&citeCnt=5&searchTerm=)

[Cervantes-Trejo, A.](https://www-scopus-com.ezproxy.lib.swin.edu.au/authid/detail.uri?origin=resultslist&authorId=56414864300&zone=), [Leenen, I.](https://www-scopus-com.ezproxy.lib.swin.edu.au/authid/detail.uri?origin=resultslist&authorId=6602486162&zone=), [Fabila-Carrasco, J.S.](https://www-scopus-com.ezproxy.lib.swin.edu.au/authid/detail.uri?origin=resultslist&authorId=57190435425&zone=), [Rojas-Vargas, R.](https://www-scopus-com.ezproxy.lib.swin.edu.au/authid/detail.uri?origin=resultslist&authorId=57190435246&zone=) 2016, [International Journal of Public Health](https://www-scopus-com.ezproxy.lib.swin.edu.au/sourceid/5300152615?origin=resultslist), 61(8), pp. 903-913

# [Determinants of road traffic safety: New evidence from Australia using state-space analysis](https://www-scopus-com.ezproxy.lib.swin.edu.au/record/display.uri?eid=2-s2.0-84971671312&origin=resultslist&sort=plf-f&src=s&st1=%28predict*+OR+model%29+AND+%28%22serious+crash*%22+OR+%22serious+accident%22+OR+%22serious+collision%22+OR+%22fatal+accident%22+OR+%22fatal+crash%22+OR+%22fatal+collision%22+OR+%22road+deaths%22+OR+%22road+fatal*%22+OR+%22traffic+fatal*%22+OR+%22collision+fatal*%22+OR+%22accident+fatal*%22%29+AND+%28driver%29+&nlo=&nlr=&nls=&sid=34fc91d90b0a1eb0b79c181c6b17236b&sot=b&sdt=b&sl=290&s=TITLE-ABS-KEY%28%28predict*+OR+model%29+AND+%28%22serious+crash*%22+OR+%22serious+accident%22+OR+%22serious+collision%22+OR+%22fatal+accident%22+OR+%22fatal+crash%22+OR+%22fatal+collision%22+OR+%22road+deaths%22+OR+%22road+fatal*%22+OR+%22traffic+fatal*%22+OR+%22collision+fatal*%22+OR+%22accident+fatal*%22%29+AND+%28driver%29+%29+AND+PUBYEAR+%3e+1983&relpos=122&citeCnt=4&searchTerm=)

[Nghiem, S.](https://www-scopus-com.ezproxy.lib.swin.edu.au/authid/detail.uri?origin=resultslist&authorId=40762082800&zone=), [Commandeur, J.J.F.](https://www-scopus-com.ezproxy.lib.swin.edu.au/authid/detail.uri?origin=resultslist&authorId=55137598600&zone=), [Connelly, L.B.](https://www-scopus-com.ezproxy.lib.swin.edu.au/authid/detail.uri?origin=resultslist&authorId=7004447218&zone=), 2016, [Accident Analysis and Prevention](https://www-scopus-com.ezproxy.lib.swin.edu.au/sourceid/19532?origin=resultslist), 94, pp. 65-72

# [Child restraint use and driver screening in fatal crashes involving drugs and alcohol](https://www-scopus-com.ezproxy.lib.swin.edu.au/record/display.uri?eid=2-s2.0-84985953080&origin=resultslist&sort=plf-f&src=s&st1=%28predict*+OR+model%29+AND+%28%22serious+crash*%22+OR+%22serious+accident%22+OR+%22serious+collision%22+OR+%22fatal+accident%22+OR+%22fatal+crash%22+OR+%22fatal+collision%22+OR+%22road+deaths%22+OR+%22road+fatal*%22+OR+%22traffic+fatal*%22+OR+%22collision+fatal*%22+OR+%22accident+fatal*%22%29+AND+%28driver%29+&nlo=&nlr=&nls=&sid=34fc91d90b0a1eb0b79c181c6b17236b&sot=b&sdt=b&sl=290&s=TITLE-ABS-KEY%28%28predict*+OR+model%29+AND+%28%22serious+crash*%22+OR+%22serious+accident%22+OR+%22serious+collision%22+OR+%22fatal+accident%22+OR+%22fatal+crash%22+OR+%22fatal+collision%22+OR+%22road+deaths%22+OR+%22road+fatal*%22+OR+%22traffic+fatal*%22+OR+%22collision+fatal*%22+OR+%22accident+fatal*%22%29+AND+%28driver%29+%29+AND+PUBYEAR+%3e+1983&relpos=124&citeCnt=4&searchTerm=)

[Huang, Y.](https://www-scopus-com.ezproxy.lib.swin.edu.au/authid/detail.uri?origin=resultslist&authorId=57191040939&zone=), [Liu, C.](https://www-scopus-com.ezproxy.lib.swin.edu.au/authid/detail.uri?origin=resultslist&authorId=57191037641&zone=), [Pressley, J.C.](https://www-scopus-com.ezproxy.lib.swin.edu.au/authid/detail.uri?origin=resultslist&authorId=6701413252&zone=) 2016. [Pediatrics](https://www-scopus-com.ezproxy.lib.swin.edu.au/sourceid/15756?origin=resultslist), 138(3),e20160319

# [Driver’s obesity and road crash risks in the United States](https://www-scopus-com.ezproxy.lib.swin.edu.au/record/display.uri?eid=2-s2.0-84975522683&origin=resultslist&sort=plf-f&src=s&st1=%28predict*+OR+model%29+AND+%28%22serious+crash*%22+OR+%22serious+accident%22+OR+%22serious+collision%22+OR+%22fatal+accident%22+OR+%22fatal+crash%22+OR+%22fatal+collision%22+OR+%22road+deaths%22+OR+%22road+fatal*%22+OR+%22traffic+fatal*%22+OR+%22collision+fatal*%22+OR+%22accident+fatal*%22%29+AND+%28driver%29+&nlo=&nlr=&nls=&sid=34fc91d90b0a1eb0b79c181c6b17236b&sot=b&sdt=b&sl=290&s=TITLE-ABS-KEY%28%28predict*+OR+model%29+AND+%28%22serious+crash*%22+OR+%22serious+accident%22+OR+%22serious+collision%22+OR+%22fatal+accident%22+OR+%22fatal+crash%22+OR+%22fatal+collision%22+OR+%22road+deaths%22+OR+%22road+fatal*%22+OR+%22traffic+fatal*%22+OR+%22collision+fatal*%22+OR+%22accident+fatal*%22%29+AND+%28driver%29+%29+AND+PUBYEAR+%3e+1983&relpos=126&citeCnt=2&searchTerm=)

[Bhatti, J.A.](https://www-scopus-com.ezproxy.lib.swin.edu.au/authid/detail.uri?origin=resultslist&authorId=25629722000&zone=), [Nathens, A.B.](https://www-scopus-com.ezproxy.lib.swin.edu.au/authid/detail.uri?origin=resultslist&authorId=7005936408&zone=), [Redelmeier, D.A.](https://www-scopus-com.ezproxy.lib.swin.edu.au/authid/detail.uri?origin=resultslist&authorId=7006152371&zone=), 2016, [Traffic Injury Prevention](https://www-scopus-com.ezproxy.lib.swin.edu.au/sourceid/22112?origin=resultslist), 17(6), pp. 604-609

# [Risk factors for the severity of injury incurred in crashes involving on-duty police cars](https://www-scopus-com.ezproxy.lib.swin.edu.au/record/display.uri?eid=2-s2.0-84974830936&origin=resultslist&sort=plf-f&src=s&st1=%28predict*+OR+model%29+AND+%28%22serious+crash*%22+OR+%22serious+accident%22+OR+%22serious+collision%22+OR+%22fatal+accident%22+OR+%22fatal+crash%22+OR+%22fatal+collision%22+OR+%22road+deaths%22+OR+%22road+fatal*%22+OR+%22traffic+fatal*%22+OR+%22collision+fatal*%22+OR+%22accident+fatal*%22%29+AND+%28driver%29+&nlo=&nlr=&nls=&sid=34fc91d90b0a1eb0b79c181c6b17236b&sot=b&sdt=b&sl=290&s=TITLE-ABS-KEY%28%28predict*+OR+model%29+AND+%28%22serious+crash*%22+OR+%22serious+accident%22+OR+%22serious+collision%22+OR+%22fatal+accident%22+OR+%22fatal+crash%22+OR+%22fatal+collision%22+OR+%22road+deaths%22+OR+%22road+fatal*%22+OR+%22traffic+fatal*%22+OR+%22collision+fatal*%22+OR+%22accident+fatal*%22%29+AND+%28driver%29+%29+AND+PUBYEAR+%3e+1983&relpos=129&citeCnt=2&searchTerm=)

[Chu, H.-C.](https://www-scopus-com.ezproxy.lib.swin.edu.au/authid/detail.uri?origin=resultslist&authorId=26640189900&zone=), 2016, [Traffic Injury Prevention](https://www-scopus-com.ezproxy.lib.swin.edu.au/sourceid/22112?origin=resultslist), 17(5), pp. 495-501

# [Exploratory multinomial logit model–based driver injury severity analyses for teenage and adult drivers in intersection-related crashes](https://www-scopus-com.ezproxy.lib.swin.edu.au/record/display.uri?eid=2-s2.0-84962852749&origin=resultslist&sort=plf-f&src=s&st1=%28predict*+OR+model%29+AND+%28%22serious+crash*%22+OR+%22serious+accident%22+OR+%22serious+collision%22+OR+%22fatal+accident%22+OR+%22fatal+crash%22+OR+%22fatal+collision%22+OR+%22road+deaths%22+OR+%22road+fatal*%22+OR+%22traffic+fatal*%22+OR+%22collision+fatal*%22+OR+%22accident+fatal*%22%29+AND+%28driver%29+&nlo=&nlr=&nls=&sid=34fc91d90b0a1eb0b79c181c6b17236b&sot=b&sdt=b&sl=290&s=TITLE-ABS-KEY%28%28predict*+OR+model%29+AND+%28%22serious+crash*%22+OR+%22serious+accident%22+OR+%22serious+collision%22+OR+%22fatal+accident%22+OR+%22fatal+crash%22+OR+%22fatal+collision%22+OR+%22road+deaths%22+OR+%22road+fatal*%22+OR+%22traffic+fatal*%22+OR+%22collision+fatal*%22+OR+%22accident+fatal*%22%29+AND+%28driver%29+%29+AND+PUBYEAR+%3e+1983&relpos=134&citeCnt=14&searchTerm=)

[Wu, Q.](https://www-scopus-com.ezproxy.lib.swin.edu.au/authid/detail.uri?origin=resultslist&authorId=56272684000&zone=), [Zhang, G.](https://www-scopus-com.ezproxy.lib.swin.edu.au/authid/detail.uri?origin=resultslist&authorId=55738935300&zone=), [Ci, Y.](https://www-scopus-com.ezproxy.lib.swin.edu.au/authid/detail.uri?origin=resultslist&authorId=24177983600&zone=), (...), [Tarefder, R.A.](https://www-scopus-com.ezproxy.lib.swin.edu.au/authid/detail.uri?origin=resultslist&authorId=57203051597&zone=), [Alcántara, A.](https://www-scopus-com.ezproxy.lib.swin.edu.au/authid/detail.uri?origin=resultslist&authorId=57188754985&zone=) 2016, [Traffic Injury Prevention](https://www-scopus-com.ezproxy.lib.swin.edu.au/sourceid/22112?origin=resultslist), 17(4), pp. 413-422

# [The association between regional environmental factors and road trauma rates: A geospatial analysis of 10 years of road traffic crashes in British Columbia, Canada](https://www-scopus-com.ezproxy.lib.swin.edu.au/record/display.uri?eid=2-s2.0-84964691748&origin=resultslist&sort=plf-f&src=s&st1=%28predict*+OR+model%29+AND+%28%22serious+crash*%22+OR+%22serious+accident%22+OR+%22serious+collision%22+OR+%22fatal+accident%22+OR+%22fatal+crash%22+OR+%22fatal+collision%22+OR+%22road+deaths%22+OR+%22road+fatal*%22+OR+%22traffic+fatal*%22+OR+%22collision+fatal*%22+OR+%22accident+fatal*%22%29+AND+%28driver%29+&nlo=&nlr=&nls=&sid=34fc91d90b0a1eb0b79c181c6b17236b&sot=b&sdt=b&sl=290&s=TITLE-ABS-KEY%28%28predict*+OR+model%29+AND+%28%22serious+crash*%22+OR+%22serious+accident%22+OR+%22serious+collision%22+OR+%22fatal+accident%22+OR+%22fatal+crash%22+OR+%22fatal+collision%22+OR+%22road+deaths%22+OR+%22road+fatal*%22+OR+%22traffic+fatal*%22+OR+%22collision+fatal*%22+OR+%22accident+fatal*%22%29+AND+%28driver%29+%29+AND+PUBYEAR+%3e+1983&relpos=137&citeCnt=4&searchTerm=)

[Brubacher, J.R.](https://www-scopus-com.ezproxy.lib.swin.edu.au/authid/detail.uri?origin=resultslist&authorId=6701493828&zone=), [Chan, H.](https://www-scopus-com.ezproxy.lib.swin.edu.au/authid/detail.uri?origin=resultslist&authorId=55635884900&zone=), [Erdelyi, S.](https://www-scopus-com.ezproxy.lib.swin.edu.au/authid/detail.uri?origin=resultslist&authorId=56364695500&zone=), [Schuurman, N.](https://www-scopus-com.ezproxy.lib.swin.edu.au/authid/detail.uri?origin=resultslist&authorId=6602954500&zone=), [Amram, O.](https://www-scopus-com.ezproxy.lib.swin.edu.au/authid/detail.uri?origin=resultslist&authorId=36443332300&zone=), 2016, [PLoS ONE](https://www-scopus-com.ezproxy.lib.swin.edu.au/sourceid/10600153309?origin=resultslist), 11(4),e0153742

# [Enforcement uniquely predicts reductions in alcohol-impaired crash fatalities](https://www-scopus-com.ezproxy.lib.swin.edu.au/record/display.uri?eid=2-s2.0-84957683601&origin=resultslist&sort=plf-f&src=s&st1=%28predict*+OR+model%29+AND+%28%22serious+crash*%22+OR+%22serious+accident%22+OR+%22serious+collision%22+OR+%22fatal+accident%22+OR+%22fatal+crash%22+OR+%22fatal+collision%22+OR+%22road+deaths%22+OR+%22road+fatal*%22+OR+%22traffic+fatal*%22+OR+%22collision+fatal*%22+OR+%22accident+fatal*%22%29+AND+%28driver%29+&nlo=&nlr=&nls=&sid=34fc91d90b0a1eb0b79c181c6b17236b&sot=b&sdt=b&sl=290&s=TITLE-ABS-KEY%28%28predict*+OR+model%29+AND+%28%22serious+crash*%22+OR+%22serious+accident%22+OR+%22serious+collision%22+OR+%22fatal+accident%22+OR+%22fatal+crash%22+OR+%22fatal+collision%22+OR+%22road+deaths%22+OR+%22road+fatal*%22+OR+%22traffic+fatal*%22+OR+%22collision+fatal*%22+OR+%22accident+fatal*%22%29+AND+%28driver%29+%29+AND+PUBYEAR+%3e+1983&relpos=140&citeCnt=10&searchTerm=)

[Yao, J.](https://www-scopus-com.ezproxy.lib.swin.edu.au/authid/detail.uri?origin=resultslist&authorId=57104122600&zone=), [Johnson, M.B.](https://www-scopus-com.ezproxy.lib.swin.edu.au/authid/detail.uri?origin=resultslist&authorId=55723923800&zone=), [Tippetts, S.](https://www-scopus-com.ezproxy.lib.swin.edu.au/authid/detail.uri?origin=resultslist&authorId=8642063600&zone=) 2016, [Addiction](https://www-scopus-com.ezproxy.lib.swin.edu.au/sourceid/24745?origin=resultslist), 111(3), pp. 448-453

# [Assessing the impact of twenty underage drinking laws](https://www-scopus-com.ezproxy.lib.swin.edu.au/record/display.uri?eid=2-s2.0-84961637030&origin=resultslist&sort=plf-f&src=s&st1=%28predict*+OR+model%29+AND+%28%22serious+crash*%22+OR+%22serious+accident%22+OR+%22serious+collision%22+OR+%22fatal+accident%22+OR+%22fatal+crash%22+OR+%22fatal+collision%22+OR+%22road+deaths%22+OR+%22road+fatal*%22+OR+%22traffic+fatal*%22+OR+%22collision+fatal*%22+OR+%22accident+fatal*%22%29+AND+%28driver%29+&nlo=&nlr=&nls=&sid=34fc91d90b0a1eb0b79c181c6b17236b&sot=b&sdt=b&sl=290&s=TITLE-ABS-KEY%28%28predict*+OR+model%29+AND+%28%22serious+crash*%22+OR+%22serious+accident%22+OR+%22serious+collision%22+OR+%22fatal+accident%22+OR+%22fatal+crash%22+OR+%22fatal+collision%22+OR+%22road+deaths%22+OR+%22road+fatal*%22+OR+%22traffic+fatal*%22+OR+%22collision+fatal*%22+OR+%22accident+fatal*%22%29+AND+%28driver%29+%29+AND+PUBYEAR+%3e+1983&relpos=141&citeCnt=17&searchTerm=)

[Fell, J.C.](https://www-scopus-com.ezproxy.lib.swin.edu.au/authid/detail.uri?origin=resultslist&authorId=7103321984&zone=), [Scherer, M.](https://www-scopus-com.ezproxy.lib.swin.edu.au/authid/detail.uri?origin=resultslist&authorId=36884653200&zone=), [Thomas, S.](https://www-scopus-com.ezproxy.lib.swin.edu.au/authid/detail.uri?origin=resultslist&authorId=56687719500&zone=), [Voas, R.B.](https://www-scopus-com.ezproxy.lib.swin.edu.au/authid/detail.uri?origin=resultslist&authorId=7006351337&zone=) 2016, [Journal of Studies on Alcohol and Drugs](https://www-scopus-com.ezproxy.lib.swin.edu.au/sourceid/7000153203?origin=resultslist), 77(2), pp. 249-260

# [The odds of wrong-way crashes and resulting fatalities: A comprehensive analysis](https://www-scopus-com.ezproxy.lib.swin.edu.au/record/display.uri?eid=2-s2.0-84952360672&origin=resultslist&sort=plf-f&src=s&st1=%28predict*+OR+model%29+AND+%28%22serious+crash*%22+OR+%22serious+accident%22+OR+%22serious+collision%22+OR+%22fatal+accident%22+OR+%22fatal+crash%22+OR+%22fatal+collision%22+OR+%22road+deaths%22+OR+%22road+fatal*%22+OR+%22traffic+fatal*%22+OR+%22collision+fatal*%22+OR+%22accident+fatal*%22%29+AND+%28driver%29+&nlo=&nlr=&nls=&sid=34fc91d90b0a1eb0b79c181c6b17236b&sot=b&sdt=b&sl=290&s=TITLE-ABS-KEY%28%28predict*+OR+model%29+AND+%28%22serious+crash*%22+OR+%22serious+accident%22+OR+%22serious+collision%22+OR+%22fatal+accident%22+OR+%22fatal+crash%22+OR+%22fatal+collision%22+OR+%22road+deaths%22+OR+%22road+fatal*%22+OR+%22traffic+fatal*%22+OR+%22collision+fatal*%22+OR+%22accident+fatal*%22%29+AND+%28driver%29+%29+AND+PUBYEAR+%3e+1983&relpos=142&citeCnt=9&searchTerm=)

[Ponnaluri, R.V.](https://www-scopus-com.ezproxy.lib.swin.edu.au/authid/detail.uri?origin=resultslist&authorId=8666410900&zone=) 2016, [Accident Analysis and Prevention](https://www-scopus-com.ezproxy.lib.swin.edu.au/sourceid/19532?origin=resultslist), 88, pp. 105-116

# [Examining the factors affecting the severity of run-off-road crashes in Abu Dhabi](https://www-scopus-com.ezproxy.lib.swin.edu.au/record/display.uri?eid=2-s2.0-84957059524&origin=resultslist&sort=plf-f&src=s&st1=%28predict*+OR+model%29+AND+%28%22serious+crash*%22+OR+%22serious+accident%22+OR+%22serious+collision%22+OR+%22fatal+accident%22+OR+%22fatal+crash%22+OR+%22fatal+collision%22+OR+%22road+deaths%22+OR+%22road+fatal*%22+OR+%22traffic+fatal*%22+OR+%22collision+fatal*%22+OR+%22accident+fatal*%22%29+AND+%28driver%29+&nlo=&nlr=&nls=&sid=34fc91d90b0a1eb0b79c181c6b17236b&sot=b&sdt=b&sl=290&s=TITLE-ABS-KEY%28%28predict*+OR+model%29+AND+%28%22serious+crash*%22+OR+%22serious+accident%22+OR+%22serious+collision%22+OR+%22fatal+accident%22+OR+%22fatal+crash%22+OR+%22fatal+collision%22+OR+%22road+deaths%22+OR+%22road+fatal*%22+OR+%22traffic+fatal*%22+OR+%22collision+fatal*%22+OR+%22accident+fatal*%22%29+AND+%28driver%29+%29+AND+PUBYEAR+%3e+1983&relpos=160&citeCnt=3&searchTerm=)

[Shawky, M.](https://www-scopus-com.ezproxy.lib.swin.edu.au/authid/detail.uri?origin=resultslist&authorId=57095714700&zone=), [Hassan, H.M.](https://www-scopus-com.ezproxy.lib.swin.edu.au/authid/detail.uri?origin=resultslist&authorId=24491775800&zone=), [Garib, A.M.](https://www-scopus-com.ezproxy.lib.swin.edu.au/authid/detail.uri?origin=resultslist&authorId=6507026565&zone=), [Al-Harthei, H.A.](https://www-scopus-com.ezproxy.lib.swin.edu.au/authid/detail.uri?origin=resultslist&authorId=12805175800&zone=) 2015, [Canadian Journal of Civil Engineering](https://www-scopus-com.ezproxy.lib.swin.edu.au/sourceid/14976?origin=resultslist), 43(2), pp. 132-138

# [Using multi-regression to analyze and predict road traffic safety level in China](https://www-scopus-com.ezproxy.lib.swin.edu.au/record/display.uri?eid=2-s2.0-84960172192&origin=resultslist&sort=plf-f&src=s&st1=%28predict*+OR+model%29+AND+%28%22serious+crash*%22+OR+%22serious+accident%22+OR+%22serious+collision%22+OR+%22fatal+accident%22+OR+%22fatal+crash%22+OR+%22fatal+collision%22+OR+%22road+deaths%22+OR+%22road+fatal*%22+OR+%22traffic+fatal*%22+OR+%22collision+fatal*%22+OR+%22accident+fatal*%22%29+AND+%28driver%29+&nlo=&nlr=&nls=&sid=34fc91d90b0a1eb0b79c181c6b17236b&sot=b&sdt=b&sl=290&s=TITLE-ABS-KEY%28%28predict*+OR+model%29+AND+%28%22serious+crash*%22+OR+%22serious+accident%22+OR+%22serious+collision%22+OR+%22fatal+accident%22+OR+%22fatal+crash%22+OR+%22fatal+collision%22+OR+%22road+deaths%22+OR+%22road+fatal*%22+OR+%22traffic+fatal*%22+OR+%22collision+fatal*%22+OR+%22accident+fatal*%22%29+AND+%28driver%29+%29+AND+PUBYEAR+%3e+1983&relpos=165&citeCnt=1&searchTerm=)

[Cai, H.](https://www-scopus-com.ezproxy.lib.swin.edu.au/authid/detail.uri?origin=resultslist&authorId=57199361133&zone=), [Zhu, D.](https://www-scopus-com.ezproxy.lib.swin.edu.au/authid/detail.uri?origin=resultslist&authorId=56323361300&zone=), [Yan, L.](https://www-scopus-com.ezproxy.lib.swin.edu.au/authid/detail.uri?origin=resultslist&authorId=56323872100&zone=) 2015, ICTIS 2015 - 3rd International Conference on Transportation Information and Safety, Proceedings, 7232140, pp. 363-369

# [Acceptance of drinking and driving and alcohol-involved driving crashes in California](https://www-scopus-com.ezproxy.lib.swin.edu.au/record/display.uri?eid=2-s2.0-84929167770&origin=resultslist&sort=plf-f&src=s&st1=%28predict*+OR+model%29+AND+%28%22serious+crash*%22+OR+%22serious+accident%22+OR+%22serious+collision%22+OR+%22fatal+accident%22+OR+%22fatal+crash%22+OR+%22fatal+collision%22+OR+%22road+deaths%22+OR+%22road+fatal*%22+OR+%22traffic+fatal*%22+OR+%22collision+fatal*%22+OR+%22accident+fatal*%22%29+AND+%28driver%29+&nlo=&nlr=&nls=&sid=34fc91d90b0a1eb0b79c181c6b17236b&sot=b&sdt=b&sl=290&s=TITLE-ABS-KEY%28%28predict*+OR+model%29+AND+%28%22serious+crash*%22+OR+%22serious+accident%22+OR+%22serious+collision%22+OR+%22fatal+accident%22+OR+%22fatal+crash%22+OR+%22fatal+collision%22+OR+%22road+deaths%22+OR+%22road+fatal*%22+OR+%22traffic+fatal*%22+OR+%22collision+fatal*%22+OR+%22accident+fatal*%22%29+AND+%28driver%29+%29+AND+PUBYEAR+%3e+1983&relpos=166&citeCnt=7&searchTerm=)

[Macleod, K.E.](https://www-scopus-com.ezproxy.lib.swin.edu.au/authid/detail.uri?origin=resultslist&authorId=7101625384&zone=), [Karriker-Jaffe, K.J.](https://www-scopus-com.ezproxy.lib.swin.edu.au/authid/detail.uri?origin=resultslist&authorId=15759428400&zone=), [Ragland, D.R.](https://www-scopus-com.ezproxy.lib.swin.edu.au/authid/detail.uri?origin=resultslist&authorId=7006408813&zone=), (...), [Kelley-Baker, T.](https://www-scopus-com.ezproxy.lib.swin.edu.au/authid/detail.uri?origin=resultslist&authorId=12779383600&zone=), [Lacey, J.H.](https://www-scopus-com.ezproxy.lib.swin.edu.au/authid/detail.uri?origin=resultslist&authorId=36839324000&zone=) 2015, [Accident Analysis and Prevention](https://www-scopus-com.ezproxy.lib.swin.edu.au/sourceid/19532?origin=resultslist), 81,3807, pp. 134-142

# [The combined effects of alcohol and cannabis on driving: Impact on crash risk](https://www-scopus-com.ezproxy.lib.swin.edu.au/record/display.uri?eid=2-s2.0-84937047394&origin=resultslist&sort=plf-f&src=s&st1=%28predict*+OR+model%29+AND+%28%22serious+crash*%22+OR+%22serious+accident%22+OR+%22serious+collision%22+OR+%22fatal+accident%22+OR+%22fatal+crash%22+OR+%22fatal+collision%22+OR+%22road+deaths%22+OR+%22road+fatal*%22+OR+%22traffic+fatal*%22+OR+%22collision+fatal*%22+OR+%22accident+fatal*%22%29+AND+%28driver%29+&nlo=&nlr=&nls=&sid=34fc91d90b0a1eb0b79c181c6b17236b&sot=b&sdt=b&sl=290&s=TITLE-ABS-KEY%28%28predict*+OR+model%29+AND+%28%22serious+crash*%22+OR+%22serious+accident%22+OR+%22serious+collision%22+OR+%22fatal+accident%22+OR+%22fatal+crash%22+OR+%22fatal+collision%22+OR+%22road+deaths%22+OR+%22road+fatal*%22+OR+%22traffic+fatal*%22+OR+%22collision+fatal*%22+OR+%22accident+fatal*%22%29+AND+%28driver%29+%29+AND+PUBYEAR+%3e+1983&relpos=171&citeCnt=40&searchTerm=)

[Dubois, S.](https://www-scopus-com.ezproxy.lib.swin.edu.au/authid/detail.uri?origin=resultslist&authorId=7005784023&zone=), [Mullen, N.](https://www-scopus-com.ezproxy.lib.swin.edu.au/authid/detail.uri?origin=resultslist&authorId=24402570600&zone=), [Weaver, B.](https://www-scopus-com.ezproxy.lib.swin.edu.au/authid/detail.uri?origin=resultslist&authorId=7102533570&zone=), [Bédard, M.](https://www-scopus-com.ezproxy.lib.swin.edu.au/authid/detail.uri?origin=resultslist&authorId=35594547000&zone=) 2015, [Forensic Science International](https://www-scopus-com.ezproxy.lib.swin.edu.au/sourceid/27743?origin=resultslist), 248, pp. 94-100

# [The Underestimated Drink Driving Situation and the Effects of Zero Tolerance Laws in China](https://www-scopus-com.ezproxy.lib.swin.edu.au/record/display.uri?eid=2-s2.0-84923623910&origin=resultslist&sort=plf-f&src=s&st1=%28predict*+OR+model%29+AND+%28%22serious+crash*%22+OR+%22serious+accident%22+OR+%22serious+collision%22+OR+%22fatal+accident%22+OR+%22fatal+crash%22+OR+%22fatal+collision%22+OR+%22road+deaths%22+OR+%22road+fatal*%22+OR+%22traffic+fatal*%22+OR+%22collision+fatal*%22+OR+%22accident+fatal*%22%29+AND+%28driver%29+&nlo=&nlr=&nls=&sid=34fc91d90b0a1eb0b79c181c6b17236b&sot=b&sdt=b&sl=290&s=TITLE-ABS-KEY%28%28predict*+OR+model%29+AND+%28%22serious+crash*%22+OR+%22serious+accident%22+OR+%22serious+collision%22+OR+%22fatal+accident%22+OR+%22fatal+crash%22+OR+%22fatal+collision%22+OR+%22road+deaths%22+OR+%22road+fatal*%22+OR+%22traffic+fatal*%22+OR+%22collision+fatal*%22+OR+%22accident+fatal*%22%29+AND+%28driver%29+%29+AND+PUBYEAR+%3e+1983&relpos=175&citeCnt=9&searchTerm=)

[Wang, Z.](https://www-scopus-com.ezproxy.lib.swin.edu.au/authid/detail.uri?origin=resultslist&authorId=55969106800&zone=), [Zhang, Y.](https://www-scopus-com.ezproxy.lib.swin.edu.au/authid/detail.uri?origin=resultslist&authorId=56006562200&zone=), [Zhou, P.](https://www-scopus-com.ezproxy.lib.swin.edu.au/authid/detail.uri?origin=resultslist&authorId=57208890757&zone=), (...), [Liu, R.](https://www-scopus-com.ezproxy.lib.swin.edu.au/authid/detail.uri?origin=resultslist&authorId=56083637800&zone=), [Jiang, C.](https://www-scopus-com.ezproxy.lib.swin.edu.au/authid/detail.uri?origin=resultslist&authorId=55774576200&zone=) 2015, [Traffic Injury Prevention](https://www-scopus-com.ezproxy.lib.swin.edu.au/sourceid/22112?origin=resultslist), 16(5), pp. 429-434

# [Exploring driver injury severity at intersection: An ordered probit analysis](https://www-scopus-com.ezproxy.lib.swin.edu.au/record/display.uri?eid=2-s2.0-84964505236&origin=resultslist&sort=plf-f&src=s&st1=%28predict*+OR+model%29+AND+%28%22serious+crash*%22+OR+%22serious+accident%22+OR+%22serious+collision%22+OR+%22fatal+accident%22+OR+%22fatal+crash%22+OR+%22fatal+collision%22+OR+%22road+deaths%22+OR+%22road+fatal*%22+OR+%22traffic+fatal*%22+OR+%22collision+fatal*%22+OR+%22accident+fatal*%22%29+AND+%28driver%29+&nlo=&nlr=&nls=&sid=34fc91d90b0a1eb0b79c181c6b17236b&sot=b&sdt=b&sl=290&s=TITLE-ABS-KEY%28%28predict*+OR+model%29+AND+%28%22serious+crash*%22+OR+%22serious+accident%22+OR+%22serious+collision%22+OR+%22fatal+accident%22+OR+%22fatal+crash%22+OR+%22fatal+collision%22+OR+%22road+deaths%22+OR+%22road+fatal*%22+OR+%22traffic+fatal*%22+OR+%22collision+fatal*%22+OR+%22accident+fatal*%22%29+AND+%28driver%29+%29+AND+PUBYEAR+%3e+1983&relpos=178&citeCnt=3&searchTerm=)

[Zhang, Y.](https://www-scopus-com.ezproxy.lib.swin.edu.au/authid/detail.uri?origin=resultslist&authorId=35232014700&zone=), [Fu, C.](https://www-scopus-com.ezproxy.lib.swin.edu.au/authid/detail.uri?origin=resultslist&authorId=49663195400&zone=), [Cheng, S.](https://www-scopus-com.ezproxy.lib.swin.edu.au/authid/detail.uri?origin=resultslist&authorId=11240749300&zone=) 2015. [Advances in Mechanical Engineering](https://www-scopus-com.ezproxy.lib.swin.edu.au/sourceid/19700182334?origin=resultslist), 7(2),567124

# [Effects of demographic and driver factors on single-vehicle and multivehicle fatal crashes investigation with multinomial logistic regression](https://www-scopus-com.ezproxy.lib.swin.edu.au/record/display.uri?eid=2-s2.0-84980383413&origin=resultslist&sort=plf-f&src=s&st1=%28predict*+OR+model%29+AND+%28%22serious+crash*%22+OR+%22serious+accident%22+OR+%22serious+collision%22+OR+%22fatal+accident%22+OR+%22fatal+crash%22+OR+%22fatal+collision%22+OR+%22road+deaths%22+OR+%22road+fatal*%22+OR+%22traffic+fatal*%22+OR+%22collision+fatal*%22+OR+%22accident+fatal*%22%29+AND+%28driver%29+&nlo=&nlr=&nls=&sid=34fc91d90b0a1eb0b79c181c6b17236b&sot=b&sdt=b&sl=290&s=TITLE-ABS-KEY%28%28predict*+OR+model%29+AND+%28%22serious+crash*%22+OR+%22serious+accident%22+OR+%22serious+collision%22+OR+%22fatal+accident%22+OR+%22fatal+crash%22+OR+%22fatal+collision%22+OR+%22road+deaths%22+OR+%22road+fatal*%22+OR+%22traffic+fatal*%22+OR+%22collision+fatal*%22+OR+%22accident+fatal*%22%29+AND+%28driver%29+%29+AND+PUBYEAR+%3e+1983&relpos=179&citeCnt=2&searchTerm=)

[Kumfer, W.](https://www-scopus-com.ezproxy.lib.swin.edu.au/authid/detail.uri?origin=resultslist&authorId=57189298915&zone=), [Wei, D.](https://www-scopus-com.ezproxy.lib.swin.edu.au/authid/detail.uri?origin=resultslist&authorId=55957897400&zone=), [Liu, H.](https://www-scopus-com.ezproxy.lib.swin.edu.au/authid/detail.uri?origin=resultslist&authorId=7409748749&zone=) 2015, [Transportation Research Record](https://www-scopus-com.ezproxy.lib.swin.edu.au/sourceid/27418?origin=resultslist), 2518, pp. 37-45

# [Explanatory and prediction power of two macro models. An application to van-involved accidents in Spain](https://www-scopus-com.ezproxy.lib.swin.edu.au/record/display.uri?eid=2-s2.0-84894309502&origin=resultslist&sort=plf-f&src=s&st1=%28predict*+OR+model%29+AND+%28%22serious+crash*%22+OR+%22serious+accident%22+OR+%22serious+collision%22+OR+%22fatal+accident%22+OR+%22fatal+crash%22+OR+%22fatal+collision%22+OR+%22road+deaths%22+OR+%22road+fatal*%22+OR+%22traffic+fatal*%22+OR+%22collision+fatal*%22+OR+%22accident+fatal*%22%29+AND+%28driver%29+&nlo=&nlr=&nls=&sid=34fc91d90b0a1eb0b79c181c6b17236b&sot=b&sdt=b&sl=290&s=TITLE-ABS-KEY%28%28predict*+OR+model%29+AND+%28%22serious+crash*%22+OR+%22serious+accident%22+OR+%22serious+collision%22+OR+%22fatal+accident%22+OR+%22fatal+crash%22+OR+%22fatal+collision%22+OR+%22road+deaths%22+OR+%22road+fatal*%22+OR+%22traffic+fatal*%22+OR+%22collision+fatal*%22+OR+%22accident+fatal*%22%29+AND+%28driver%29+%29+AND+PUBYEAR+%3e+1983&relpos=184&citeCnt=3&searchTerm=)

[Dadashova, B.](https://www-scopus-com.ezproxy.lib.swin.edu.au/authid/detail.uri?origin=resultslist&authorId=56042760000&zone=), [Ramírez Arenas, B.](https://www-scopus-com.ezproxy.lib.swin.edu.au/authid/detail.uri?origin=resultslist&authorId=56042556200&zone=), [McWilliams Mira, J.](https://www-scopus-com.ezproxy.lib.swin.edu.au/authid/detail.uri?origin=resultslist&authorId=56042914200&zone=), [Izquierdo Aparicio, F.](https://www-scopus-com.ezproxy.lib.swin.edu.au/authid/detail.uri?origin=resultslist&authorId=56043416100&zone=) 2014, [Transport Policy](https://www-scopus-com.ezproxy.lib.swin.edu.au/sourceid/20838?origin=resultslist), 32, pp. 203-217

# [The relative risk of involvement in fatal crashes as a function of race/ethnicity and blood alcohol concentration](https://www-scopus-com.ezproxy.lib.swin.edu.au/record/display.uri?eid=2-s2.0-84893033090&origin=resultslist&sort=plf-f&src=s&st1=%28predict*+OR+model%29+AND+%28%22serious+crash*%22+OR+%22serious+accident%22+OR+%22serious+collision%22+OR+%22fatal+accident%22+OR+%22fatal+crash%22+OR+%22fatal+collision%22+OR+%22road+deaths%22+OR+%22road+fatal*%22+OR+%22traffic+fatal*%22+OR+%22collision+fatal*%22+OR+%22accident+fatal*%22%29+AND+%28driver%29+&nlo=&nlr=&nls=&sid=34fc91d90b0a1eb0b79c181c6b17236b&sot=b&sdt=b&sl=290&s=TITLE-ABS-KEY%28%28predict*+OR+model%29+AND+%28%22serious+crash*%22+OR+%22serious+accident%22+OR+%22serious+collision%22+OR+%22fatal+accident%22+OR+%22fatal+crash%22+OR+%22fatal+collision%22+OR+%22road+deaths%22+OR+%22road+fatal*%22+OR+%22traffic+fatal*%22+OR+%22collision+fatal*%22+OR+%22accident+fatal*%22%29+AND+%28driver%29+%29+AND+PUBYEAR+%3e+1983&relpos=185&citeCnt=6&searchTerm=)

[Torres, P.](https://www-scopus-com.ezproxy.lib.swin.edu.au/authid/detail.uri?origin=resultslist&authorId=54394587400&zone=), [Romano, E.](https://www-scopus-com.ezproxy.lib.swin.edu.au/authid/detail.uri?origin=resultslist&authorId=7102438608&zone=), [Voas, R.B.](https://www-scopus-com.ezproxy.lib.swin.edu.au/authid/detail.uri?origin=resultslist&authorId=7006351337&zone=), [De La Rosa, M.](https://www-scopus-com.ezproxy.lib.swin.edu.au/authid/detail.uri?origin=resultslist&authorId=7101829120&zone=), [Lacey, J.H.](https://www-scopus-com.ezproxy.lib.swin.edu.au/authid/detail.uri?origin=resultslist&authorId=36839324000&zone=) 2014. [Journal of Safety Research](https://www-scopus-com.ezproxy.lib.swin.edu.au/sourceid/29284?origin=resultslist), 48, pp. 95-101

# [Effect of Passenger Presence on Older Drivers' Risk of Fatal Crash Involvement](https://www-scopus-com.ezproxy.lib.swin.edu.au/record/display.uri?eid=2-s2.0-84897042664&origin=resultslist&sort=plf-f&src=s&st1=%28predict*+OR+model%29+AND+%28%22serious+crash*%22+OR+%22serious+accident%22+OR+%22serious+collision%22+OR+%22fatal+accident%22+OR+%22fatal+crash%22+OR+%22fatal+collision%22+OR+%22road+deaths%22+OR+%22road+fatal*%22+OR+%22traffic+fatal*%22+OR+%22collision+fatal*%22+OR+%22accident+fatal*%22%29+AND+%28driver%29+&nlo=&nlr=&nls=&sid=34fc91d90b0a1eb0b79c181c6b17236b&sot=b&sdt=b&sl=290&s=TITLE-ABS-KEY%28%28predict*+OR+model%29+AND+%28%22serious+crash*%22+OR+%22serious+accident%22+OR+%22serious+collision%22+OR+%22fatal+accident%22+OR+%22fatal+crash%22+OR+%22fatal+collision%22+OR+%22road+deaths%22+OR+%22road+fatal*%22+OR+%22traffic+fatal*%22+OR+%22collision+fatal*%22+OR+%22accident+fatal*%22%29+AND+%28driver%29+%29+AND+PUBYEAR+%3e+1983&relpos=195&citeCnt=7&searchTerm=)

[Braitman, K.A.](https://www-scopus-com.ezproxy.lib.swin.edu.au/authid/detail.uri?origin=resultslist&authorId=16506205000&zone=), [Chaudhary, N.K.](https://www-scopus-com.ezproxy.lib.swin.edu.au/authid/detail.uri?origin=resultslist&authorId=7006949844&zone=), [McCartt, A.T.](https://www-scopus-com.ezproxy.lib.swin.edu.au/authid/detail.uri?origin=resultslist&authorId=7003641841&zone=) 2014, [Traffic Injury Prevention](https://www-scopus-com.ezproxy.lib.swin.edu.au/sourceid/22112?origin=resultslist), 15(5), pp. 451-456

# [Reduction in fatalities, ambulance calls, and hospital admissions for road trauma after implementation of new traffic laws](https://www-scopus-com.ezproxy.lib.swin.edu.au/record/display.uri?eid=2-s2.0-84907219828&origin=resultslist&sort=plf-f&src=s&st1=%28predict*+OR+model%29+AND+%28%22serious+crash*%22+OR+%22serious+accident%22+OR+%22serious+collision%22+OR+%22fatal+accident%22+OR+%22fatal+crash%22+OR+%22fatal+collision%22+OR+%22road+deaths%22+OR+%22road+fatal*%22+OR+%22traffic+fatal*%22+OR+%22collision+fatal*%22+OR+%22accident+fatal*%22%29+AND+%28driver%29+&nlo=&nlr=&nls=&sid=34fc91d90b0a1eb0b79c181c6b17236b&sot=b&sdt=b&sl=290&s=TITLE-ABS-KEY%28%28predict*+OR+model%29+AND+%28%22serious+crash*%22+OR+%22serious+accident%22+OR+%22serious+collision%22+OR+%22fatal+accident%22+OR+%22fatal+crash%22+OR+%22fatal+collision%22+OR+%22road+deaths%22+OR+%22road+fatal*%22+OR+%22traffic+fatal*%22+OR+%22collision+fatal*%22+OR+%22accident+fatal*%22%29+AND+%28driver%29+%29+AND+PUBYEAR+%3e+1983&relpos=199&citeCnt=19&searchTerm=)

[Brubacher, J.R.](https://www-scopus-com.ezproxy.lib.swin.edu.au/authid/detail.uri?origin=resultslist&authorId=6701493828&zone=), [Chan, H.](https://www-scopus-com.ezproxy.lib.swin.edu.au/authid/detail.uri?origin=resultslist&authorId=55635884900&zone=), [Brasher, P.](https://www-scopus-com.ezproxy.lib.swin.edu.au/authid/detail.uri?origin=resultslist&authorId=7003696820&zone=), (...), [Schuurman, N.](https://www-scopus-com.ezproxy.lib.swin.edu.au/authid/detail.uri?origin=resultslist&authorId=6602954500&zone=), [Pike, I.](https://www-scopus-com.ezproxy.lib.swin.edu.au/authid/detail.uri?origin=resultslist&authorId=8945628200&zone=) 2014, [American Journal of Public Health](https://www-scopus-com.ezproxy.lib.swin.edu.au/sourceid/19561?origin=resultslist), 104(10), pp. e89-e97

# [Examining the impact of opioid analgesics on crash responsibility in truck drivers involved in fatal crashes](https://www-scopus-com.ezproxy.lib.swin.edu.au/record/display.uri?eid=2-s2.0-84889679845&origin=resultslist&sort=plf-f&src=s&st1=%28predict*+OR+model%29+AND+%28%22serious+crash*%22+OR+%22serious+accident%22+OR+%22serious+collision%22+OR+%22fatal+accident%22+OR+%22fatal+crash%22+OR+%22fatal+collision%22+OR+%22road+deaths%22+OR+%22road+fatal*%22+OR+%22traffic+fatal*%22+OR+%22collision+fatal*%22+OR+%22accident+fatal*%22%29+AND+%28driver%29+&nlo=&nlr=&nls=&sid=34fc91d90b0a1eb0b79c181c6b17236b&sot=b&sdt=b&sl=290&s=TITLE-ABS-KEY%28%28predict*+OR+model%29+AND+%28%22serious+crash*%22+OR+%22serious+accident%22+OR+%22serious+collision%22+OR+%22fatal+accident%22+OR+%22fatal+crash%22+OR+%22fatal+collision%22+OR+%22road+deaths%22+OR+%22road+fatal*%22+OR+%22traffic+fatal*%22+OR+%22collision+fatal*%22+OR+%22accident+fatal*%22%29+AND+%28driver%29+%29+AND+PUBYEAR+%3e+1983&relpos=200&citeCnt=14&searchTerm=)

[Reguly, P.](https://www-scopus-com.ezproxy.lib.swin.edu.au/authid/detail.uri?origin=resultslist&authorId=55955399400&zone=), [Dubois, S.](https://www-scopus-com.ezproxy.lib.swin.edu.au/authid/detail.uri?origin=resultslist&authorId=7005784023&zone=), [Bédard, M.](https://www-scopus-com.ezproxy.lib.swin.edu.au/authid/detail.uri?origin=resultslist&authorId=35594547000&zone=) 2014, [Forensic Science International](https://www-scopus-com.ezproxy.lib.swin.edu.au/sourceid/27743?origin=resultslist), 234, pp. 154-161

# [Assessing factors causing severe injuries in crashes of high-deck buses in long-distance driving on freeways](https://www-scopus-com.ezproxy.lib.swin.edu.au/record/display.uri?eid=2-s2.0-84886704413&origin=resultslist&sort=plf-f&src=s&st1=%28predict*+OR+model%29+AND+%28%22serious+crash*%22+OR+%22serious+accident%22+OR+%22serious+collision%22+OR+%22fatal+accident%22+OR+%22fatal+crash%22+OR+%22fatal+collision%22+OR+%22road+deaths%22+OR+%22road+fatal*%22+OR+%22traffic+fatal*%22+OR+%22collision+fatal*%22+OR+%22accident+fatal*%22%29+AND+%28driver%29+&nlo=&nlr=&nls=&sid=34fc91d90b0a1eb0b79c181c6b17236b&sot=b&sdt=b&sl=290&s=TITLE-ABS-KEY%28%28predict*+OR+model%29+AND+%28%22serious+crash*%22+OR+%22serious+accident%22+OR+%22serious+collision%22+OR+%22fatal+accident%22+OR+%22fatal+crash%22+OR+%22fatal+collision%22+OR+%22road+deaths%22+OR+%22road+fatal*%22+OR+%22traffic+fatal*%22+OR+%22collision+fatal*%22+OR+%22accident+fatal*%22%29+AND+%28driver%29+%29+AND+PUBYEAR+%3e+1983&relpos=201&citeCnt=18&searchTerm=)

[Chu, H.-C.](https://www-scopus-com.ezproxy.lib.swin.edu.au/authid/detail.uri?origin=resultslist&authorId=26640189900&zone=) 2014, [Accident Analysis and Prevention](https://www-scopus-com.ezproxy.lib.swin.edu.au/sourceid/19532?origin=resultslist), 62, pp. 130-136

# [Prediction of road accident severity using the ordered probit model](https://www-scopus-com.ezproxy.lib.swin.edu.au/record/display.uri?eid=2-s2.0-84959332280&origin=resultslist&sort=plf-f&src=s&st1=%28predict*+OR+model%29+AND+%28%22serious+crash*%22+OR+%22serious+accident%22+OR+%22serious+collision%22+OR+%22fatal+accident%22+OR+%22fatal+crash%22+OR+%22fatal+collision%22+OR+%22road+deaths%22+OR+%22road+fatal*%22+OR+%22traffic+fatal*%22+OR+%22collision+fatal*%22+OR+%22accident+fatal*%22%29+AND+%28driver%29+&nlo=&nlr=&nls=&sid=34fc91d90b0a1eb0b79c181c6b17236b&sot=b&sdt=b&sl=290&s=TITLE-ABS-KEY%28%28predict*+OR+model%29+AND+%28%22serious+crash*%22+OR+%22serious+accident%22+OR+%22serious+collision%22+OR+%22fatal+accident%22+OR+%22fatal+crash%22+OR+%22fatal+collision%22+OR+%22road+deaths%22+OR+%22road+fatal*%22+OR+%22traffic+fatal*%22+OR+%22collision+fatal*%22+OR+%22accident+fatal*%22%29+AND+%28driver%29+%29+AND+PUBYEAR+%3e+1983&relpos=202&citeCnt=14&searchTerm=)

[Garrido, R.](https://www-scopus-com.ezproxy.lib.swin.edu.au/authid/detail.uri?origin=resultslist&authorId=57147079400&zone=), [Bastos, A.](https://www-scopus-com.ezproxy.lib.swin.edu.au/authid/detail.uri?origin=resultslist&authorId=24766285900&zone=), [De Almeida, A.](https://www-scopus-com.ezproxy.lib.swin.edu.au/authid/detail.uri?origin=resultslist&authorId=7102580984&zone=), [Elvas, J.P.](https://www-scopus-com.ezproxy.lib.swin.edu.au/authid/detail.uri?origin=resultslist&authorId=57148401800&zone=) 2014, [Transportation Research Procedia](https://www-scopus-com.ezproxy.lib.swin.edu.au/sourceid/21100448300?origin=resultslist), 3, pp. 214-223

# [Understanding geographical variations in road traffic fatalities in South Africa](https://www-scopus-com.ezproxy.lib.swin.edu.au/record/display.uri?eid=2-s2.0-84888301486&origin=resultslist&sort=plf-f&src=s&st1=%28predict*+OR+model%29+AND+%28%22serious+crash*%22+OR+%22serious+accident%22+OR+%22serious+collision%22+OR+%22fatal+accident%22+OR+%22fatal+crash%22+OR+%22fatal+collision%22+OR+%22road+deaths%22+OR+%22road+fatal*%22+OR+%22traffic+fatal*%22+OR+%22collision+fatal*%22+OR+%22accident+fatal*%22%29+AND+%28driver%29+&nlo=&nlr=&nls=&sid=34fc91d90b0a1eb0b79c181c6b17236b&sot=b&sdt=b&sl=290&s=TITLE-ABS-KEY%28%28predict*+OR+model%29+AND+%28%22serious+crash*%22+OR+%22serious+accident%22+OR+%22serious+collision%22+OR+%22fatal+accident%22+OR+%22fatal+crash%22+OR+%22fatal+collision%22+OR+%22road+deaths%22+OR+%22road+fatal*%22+OR+%22traffic+fatal*%22+OR+%22collision+fatal*%22+OR+%22accident+fatal*%22%29+AND+%28driver%29+%29+AND+PUBYEAR+%3e+1983&relpos=210&citeCnt=1&searchTerm=)

[Sukhai, A.](https://www-scopus-com.ezproxy.lib.swin.edu.au/authid/detail.uri?origin=resultslist&authorId=55882753500&zone=), [Jones, A.P.](https://www-scopus-com.ezproxy.lib.swin.edu.au/authid/detail.uri?origin=resultslist&authorId=7407101741&zone=) 2013, [South African Geographical Journal](https://www-scopus-com.ezproxy.lib.swin.edu.au/sourceid/13097?origin=resultslist), 95(2), pp. 187-204

# [Variation in U.S. traffic safety policy environments and motor vehicle fatalities 1980-2010](https://www-scopus-com.ezproxy.lib.swin.edu.au/record/display.uri?eid=2-s2.0-84889587022&origin=resultslist&sort=plf-f&src=s&st1=%28predict*+OR+model%29+AND+%28%22serious+crash*%22+OR+%22serious+accident%22+OR+%22serious+collision%22+OR+%22fatal+accident%22+OR+%22fatal+crash%22+OR+%22fatal+collision%22+OR+%22road+deaths%22+OR+%22road+fatal*%22+OR+%22traffic+fatal*%22+OR+%22collision+fatal*%22+OR+%22accident+fatal*%22%29+AND+%28driver%29+&nlo=&nlr=&nls=&sid=34fc91d90b0a1eb0b79c181c6b17236b&sot=b&sdt=b&sl=290&s=TITLE-ABS-KEY%28%28predict*+OR+model%29+AND+%28%22serious+crash*%22+OR+%22serious+accident%22+OR+%22serious+collision%22+OR+%22fatal+accident%22+OR+%22fatal+crash%22+OR+%22fatal+collision%22+OR+%22road+deaths%22+OR+%22road+fatal*%22+OR+%22traffic+fatal*%22+OR+%22collision+fatal*%22+OR+%22accident+fatal*%22%29+AND+%28driver%29+%29+AND+PUBYEAR+%3e+1983&relpos=212&citeCnt=9&searchTerm=)

[Silver, D.](https://www-scopus-com.ezproxy.lib.swin.edu.au/authid/detail.uri?origin=resultslist&authorId=7202150836&zone=), [Macinko, J.](https://www-scopus-com.ezproxy.lib.swin.edu.au/authid/detail.uri?origin=resultslist&authorId=55790171400&zone=), [Bae, J.Y.](https://www-scopus-com.ezproxy.lib.swin.edu.au/authid/detail.uri?origin=resultslist&authorId=41761012700&zone=), [Jimenez, G.](https://www-scopus-com.ezproxy.lib.swin.edu.au/authid/detail.uri?origin=resultslist&authorId=56625643900&zone=), [Paul, M.](https://www-scopus-com.ezproxy.lib.swin.edu.au/authid/detail.uri?origin=resultslist&authorId=55752647100&zone=) 2013, [Public Health](https://www-scopus-com.ezproxy.lib.swin.edu.au/sourceid/17697?origin=resultslist), 127(12), pp. 1117-1125

# [Effects of public rest areas on fatigue-related crashes](https://www-scopus-com.ezproxy.lib.swin.edu.au/record/display.uri?eid=2-s2.0-84887981928&origin=resultslist&sort=plf-f&src=s&st1=%28predict*+OR+model%29+AND+%28%22serious+crash*%22+OR+%22serious+accident%22+OR+%22serious+collision%22+OR+%22fatal+accident%22+OR+%22fatal+crash%22+OR+%22fatal+collision%22+OR+%22road+deaths%22+OR+%22road+fatal*%22+OR+%22traffic+fatal*%22+OR+%22collision+fatal*%22+OR+%22accident+fatal*%22%29+AND+%28driver%29+&nlo=&nlr=&nls=&sid=34fc91d90b0a1eb0b79c181c6b17236b&sot=b&sdt=b&sl=290&s=TITLE-ABS-KEY%28%28predict*+OR+model%29+AND+%28%22serious+crash*%22+OR+%22serious+accident%22+OR+%22serious+collision%22+OR+%22fatal+accident%22+OR+%22fatal+crash%22+OR+%22fatal+collision%22+OR+%22road+deaths%22+OR+%22road+fatal*%22+OR+%22traffic+fatal*%22+OR+%22collision+fatal*%22+OR+%22accident+fatal*%22%29+AND+%28driver%29+%29+AND+PUBYEAR+%3e+1983&relpos=213&citeCnt=4&searchTerm=)

[McArthur, A.](https://www-scopus-com.ezproxy.lib.swin.edu.au/authid/detail.uri?origin=resultslist&authorId=56089854400&zone=), [Kay, J.](https://www-scopus-com.ezproxy.lib.swin.edu.au/authid/detail.uri?origin=resultslist&authorId=56089587300&zone=), [Savolainen, P.](https://www-scopus-com.ezproxy.lib.swin.edu.au/authid/detail.uri?origin=resultslist&authorId=35410522800&zone=), [Gates, T.](https://www-scopus-com.ezproxy.lib.swin.edu.au/authid/detail.uri?origin=resultslist&authorId=55952728800&zone=) 2013, [Transportation Research Record](https://www-scopus-com.ezproxy.lib.swin.edu.au/sourceid/27418?origin=resultslist), (2386), pp. 16-25

# [The impact of state level graduated driver licensing programs on rates of passenger restraint use and unlicensed driving in fatal crashes](https://www-scopus-com.ezproxy.lib.swin.edu.au/record/display.uri?eid=2-s2.0-84888095493&origin=resultslist&sort=plf-f&src=s&st1=%28predict*+OR+model%29+AND+%28%22serious+crash*%22+OR+%22serious+accident%22+OR+%22serious+collision%22+OR+%22fatal+accident%22+OR+%22fatal+crash%22+OR+%22fatal+collision%22+OR+%22road+deaths%22+OR+%22road+fatal*%22+OR+%22traffic+fatal*%22+OR+%22collision+fatal*%22+OR+%22accident+fatal*%22%29+AND+%28driver%29+&nlo=&nlr=&nls=&sid=34fc91d90b0a1eb0b79c181c6b17236b&sot=b&sdt=b&sl=290&s=TITLE-ABS-KEY%28%28predict*+OR+model%29+AND+%28%22serious+crash*%22+OR+%22serious+accident%22+OR+%22serious+collision%22+OR+%22fatal+accident%22+OR+%22fatal+crash%22+OR+%22fatal+collision%22+OR+%22road+deaths%22+OR+%22road+fatal*%22+OR+%22traffic+fatal*%22+OR+%22collision+fatal*%22+OR+%22accident+fatal*%22%29+AND+%28driver%29+%29+AND+PUBYEAR+%3e+1983&relpos=215&citeCnt=1&searchTerm=)

[Fu, J.](https://www-scopus-com.ezproxy.lib.swin.edu.au/authid/detail.uri?origin=resultslist&authorId=54419860900&zone=), [Anderson, C.L.](https://www-scopus-com.ezproxy.lib.swin.edu.au/authid/detail.uri?origin=resultslist&authorId=35556095500&zone=), [Dziura, J.D.](https://www-scopus-com.ezproxy.lib.swin.edu.au/authid/detail.uri?origin=resultslist&authorId=6602746173&zone=), [Crowley, M.J.](https://www-scopus-com.ezproxy.lib.swin.edu.au/authid/detail.uri?origin=resultslist&authorId=7103054032&zone=), [Vaca, F.E.](https://www-scopus-com.ezproxy.lib.swin.edu.au/authid/detail.uri?origin=resultslist&authorId=7003561651&zone=) 2013, [Annals of Advances in Automotive Medicine](https://www-scopus-com.ezproxy.lib.swin.edu.au/sourceid/19700188374?origin=resultslist), 57, pp. 89-98

# [Drug use and fatal motor vehicle crashes: A case-control study](https://www-scopus-com.ezproxy.lib.swin.edu.au/record/display.uri?eid=2-s2.0-84884614373&origin=resultslist&sort=plf-f&src=s&st1=%28predict*+OR+model%29+AND+%28%22serious+crash*%22+OR+%22serious+accident%22+OR+%22serious+collision%22+OR+%22fatal+accident%22+OR+%22fatal+crash%22+OR+%22fatal+collision%22+OR+%22road+deaths%22+OR+%22road+fatal*%22+OR+%22traffic+fatal*%22+OR+%22collision+fatal*%22+OR+%22accident+fatal*%22%29+AND+%28driver%29+&nlo=&nlr=&nls=&sid=34fc91d90b0a1eb0b79c181c6b17236b&sot=b&sdt=b&sl=290&s=TITLE-ABS-KEY%28%28predict*+OR+model%29+AND+%28%22serious+crash*%22+OR+%22serious+accident%22+OR+%22serious+collision%22+OR+%22fatal+accident%22+OR+%22fatal+crash%22+OR+%22fatal+collision%22+OR+%22road+deaths%22+OR+%22road+fatal*%22+OR+%22traffic+fatal*%22+OR+%22collision+fatal*%22+OR+%22accident+fatal*%22%29+AND+%28driver%29+%29+AND+PUBYEAR+%3e+1983&relpos=218&citeCnt=64&searchTerm=)

[Li, G.](https://www-scopus-com.ezproxy.lib.swin.edu.au/authid/detail.uri?origin=resultslist&authorId=55547060800&zone=), [Brady, J.E.](https://www-scopus-com.ezproxy.lib.swin.edu.au/authid/detail.uri?origin=resultslist&authorId=15828779600&zone=), [Chen, Q.](https://www-scopus-com.ezproxy.lib.swin.edu.au/authid/detail.uri?origin=resultslist&authorId=36139322800&zone=) 2013, [Accident Analysis and Prevention](https://www-scopus-com.ezproxy.lib.swin.edu.au/sourceid/19532?origin=resultslist), 60, pp. 205-210

# Patterns of drug use in fatal crashes

[Romano, E.](https://www-scopus-com.ezproxy.lib.swin.edu.au/authid/detail.uri?origin=resultslist&authorId=7102438608&zone=), [Pollini, R.A.](https://www-scopus-com.ezproxy.lib.swin.edu.au/authid/detail.uri?origin=resultslist&authorId=8528310000&zone=), 2013, [Addiction](https://www-scopus-com.ezproxy.lib.swin.edu.au/sourceid/24745?origin=resultslist), 108(8), pp. 1428-1438

# [The effect of the learner license Graduated Driver Licensing components on teen drivers' crashes](https://www-scopus-com.ezproxy.lib.swin.edu.au/record/display.uri?eid=2-s2.0-84880276443&origin=resultslist&sort=plf-f&src=s&st1=%28predict*+OR+model%29+AND+%28%22serious+crash*%22+OR+%22serious+accident%22+OR+%22serious+collision%22+OR+%22fatal+accident%22+OR+%22fatal+crash%22+OR+%22fatal+collision%22+OR+%22road+deaths%22+OR+%22road+fatal*%22+OR+%22traffic+fatal*%22+OR+%22collision+fatal*%22+OR+%22accident+fatal*%22%29+AND+%28driver%29+&nlo=&nlr=&nls=&sid=34fc91d90b0a1eb0b79c181c6b17236b&sot=b&sdt=b&sl=290&s=TITLE-ABS-KEY%28%28predict*+OR+model%29+AND+%28%22serious+crash*%22+OR+%22serious+accident%22+OR+%22serious+collision%22+OR+%22fatal+accident%22+OR+%22fatal+crash%22+OR+%22fatal+collision%22+OR+%22road+deaths%22+OR+%22road+fatal*%22+OR+%22traffic+fatal*%22+OR+%22collision+fatal*%22+OR+%22accident+fatal*%22%29+AND+%28driver%29+%29+AND+PUBYEAR+%3e+1983&relpos=221&citeCnt=18&searchTerm=)

[Ehsani, J.P.](https://www-scopus-com.ezproxy.lib.swin.edu.au/authid/detail.uri?origin=resultslist&authorId=55397644700&zone=), [Raymond Bingham, C.](https://www-scopus-com.ezproxy.lib.swin.edu.au/authid/detail.uri?origin=resultslist&authorId=34969044900&zone=), [Shope, J.T.](https://www-scopus-com.ezproxy.lib.swin.edu.au/authid/detail.uri?origin=resultslist&authorId=7005362795&zone=) 2013, [Accident Analysis and Prevention](https://www-scopus-com.ezproxy.lib.swin.edu.au/sourceid/19532?origin=resultslist), 59, pp. 327-336

# [Onset of a declining trend in fatal motor vehicle crashes involving drunk-driving in japan](https://www-scopus-com.ezproxy.lib.swin.edu.au/record/display.uri?eid=2-s2.0-84879981284&origin=resultslist&sort=plf-f&src=s&st1=%28predict*+OR+model%29+AND+%28%22serious+crash*%22+OR+%22serious+accident%22+OR+%22serious+collision%22+OR+%22fatal+accident%22+OR+%22fatal+crash%22+OR+%22fatal+collision%22+OR+%22road+deaths%22+OR+%22road+fatal*%22+OR+%22traffic+fatal*%22+OR+%22collision+fatal*%22+OR+%22accident+fatal*%22%29+AND+%28driver%29+&nlo=&nlr=&nls=&sid=34fc91d90b0a1eb0b79c181c6b17236b&sot=b&sdt=b&sl=290&s=TITLE-ABS-KEY%28%28predict*+OR+model%29+AND+%28%22serious+crash*%22+OR+%22serious+accident%22+OR+%22serious+collision%22+OR+%22fatal+accident%22+OR+%22fatal+crash%22+OR+%22fatal+collision%22+OR+%22road+deaths%22+OR+%22road+fatal*%22+OR+%22traffic+fatal*%22+OR+%22collision+fatal*%22+OR+%22accident+fatal*%22%29+AND+%28driver%29+%29+AND+PUBYEAR+%3e+1983&relpos=222&citeCnt=12&searchTerm=)

[Nakahara, S.](https://www-scopus-com.ezproxy.lib.swin.edu.au/authid/detail.uri?origin=resultslist&authorId=7102955427&zone=), [Katanoda, K.](https://www-scopus-com.ezproxy.lib.swin.edu.au/authid/detail.uri?origin=resultslist&authorId=15055917700&zone=), [Ichikawa, M.](https://www-scopus-com.ezproxy.lib.swin.edu.au/authid/detail.uri?origin=resultslist&authorId=7402185227&zone=) 2013, [Journal of Epidemiology](https://www-scopus-com.ezproxy.lib.swin.edu.au/sourceid/15894?origin=resultslist), 23(3), pp. 195-204

# [Fuel economy and safety: The influences of vehicle class and driver behavior](https://www-scopus-com.ezproxy.lib.swin.edu.au/record/display.uri?eid=2-s2.0-84880597746&origin=resultslist&sort=plf-f&src=s&st1=%28predict*+OR+model%29+AND+%28%22serious+crash*%22+OR+%22serious+accident%22+OR+%22serious+collision%22+OR+%22fatal+accident%22+OR+%22fatal+crash%22+OR+%22fatal+collision%22+OR+%22road+deaths%22+OR+%22road+fatal*%22+OR+%22traffic+fatal*%22+OR+%22collision+fatal*%22+OR+%22accident+fatal*%22%29+AND+%28driver%29+&nlo=&nlr=&nls=&sid=34fc91d90b0a1eb0b79c181c6b17236b&sot=b&sdt=b&sl=290&s=TITLE-ABS-KEY%28%28predict*+OR+model%29+AND+%28%22serious+crash*%22+OR+%22serious+accident%22+OR+%22serious+collision%22+OR+%22fatal+accident%22+OR+%22fatal+crash%22+OR+%22fatal+collision%22+OR+%22road+deaths%22+OR+%22road+fatal*%22+OR+%22traffic+fatal*%22+OR+%22collision+fatal*%22+OR+%22accident+fatal*%22%29+AND+%28driver%29+%29+AND+PUBYEAR+%3e+1983&relpos=223&citeCnt=21&searchTerm=)

[Jacobsen, M.R.](https://www-scopus-com.ezproxy.lib.swin.edu.au/authid/detail.uri?origin=resultslist&authorId=14070351900&zone=) 2013, [American Economic Journal: Applied Economics](https://www-scopus-com.ezproxy.lib.swin.edu.au/sourceid/19900191767?origin=resultslist), 5(3), pp. 1-26

# [Fatal connections-socioeconomic determinants of road accident risk and drunk driving in Sweden](https://www-scopus-com.ezproxy.lib.swin.edu.au/record/display.uri?eid=2-s2.0-84879083537&origin=resultslist&sort=plf-f&src=s&st1=%28predict*+OR+model%29+AND+%28%22serious+crash*%22+OR+%22serious+accident%22+OR+%22serious+collision%22+OR+%22fatal+accident%22+OR+%22fatal+crash%22+OR+%22fatal+collision%22+OR+%22road+deaths%22+OR+%22road+fatal*%22+OR+%22traffic+fatal*%22+OR+%22collision+fatal*%22+OR+%22accident+fatal*%22%29+AND+%28driver%29+&nlo=&nlr=&nls=&sid=34fc91d90b0a1eb0b79c181c6b17236b&sot=b&sdt=b&sl=290&s=TITLE-ABS-KEY%28%28predict*+OR+model%29+AND+%28%22serious+crash*%22+OR+%22serious+accident%22+OR+%22serious+collision%22+OR+%22fatal+accident%22+OR+%22fatal+crash%22+OR+%22fatal+collision%22+OR+%22road+deaths%22+OR+%22road+fatal*%22+OR+%22traffic+fatal*%22+OR+%22collision+fatal*%22+OR+%22accident+fatal*%22%29+AND+%28driver%29+%29+AND+PUBYEAR+%3e+1983&relpos=226&citeCnt=5&searchTerm=)

[Krüger, N.A.](https://www-scopus-com.ezproxy.lib.swin.edu.au/authid/detail.uri?origin=resultslist&authorId=26032629600&zone=) 2013, [Journal of Safety Research](https://www-scopus-com.ezproxy.lib.swin.edu.au/sourceid/29284?origin=resultslist), 46, pp. 59-65

# [Risk factors associated with traffic violations and accident severity in China](https://www-scopus-com.ezproxy.lib.swin.edu.au/record/display.uri?eid=2-s2.0-84878695008&origin=resultslist&sort=plf-f&src=s&st1=%28predict*+OR+model%29+AND+%28%22serious+crash*%22+OR+%22serious+accident%22+OR+%22serious+collision%22+OR+%22fatal+accident%22+OR+%22fatal+crash%22+OR+%22fatal+collision%22+OR+%22road+deaths%22+OR+%22road+fatal*%22+OR+%22traffic+fatal*%22+OR+%22collision+fatal*%22+OR+%22accident+fatal*%22%29+AND+%28driver%29+&nlo=&nlr=&nls=&sid=34fc91d90b0a1eb0b79c181c6b17236b&sot=b&sdt=b&sl=290&s=TITLE-ABS-KEY%28%28predict*+OR+model%29+AND+%28%22serious+crash*%22+OR+%22serious+accident%22+OR+%22serious+collision%22+OR+%22fatal+accident%22+OR+%22fatal+crash%22+OR+%22fatal+collision%22+OR+%22road+deaths%22+OR+%22road+fatal*%22+OR+%22traffic+fatal*%22+OR+%22collision+fatal*%22+OR+%22accident+fatal*%22%29+AND+%28driver%29+%29+AND+PUBYEAR+%3e+1983&relpos=227&citeCnt=88&searchTerm=)

[Zhang, G.](https://www-scopus-com.ezproxy.lib.swin.edu.au/authid/detail.uri?origin=resultslist&authorId=57198462538&zone=), [Yau, K.K.W.](https://www-scopus-com.ezproxy.lib.swin.edu.au/authid/detail.uri?origin=resultslist&authorId=7101941425&zone=), [Chen, G.](https://www-scopus-com.ezproxy.lib.swin.edu.au/authid/detail.uri?origin=resultslist&authorId=55758475900&zone=) 2013, [Accident Analysis and Prevention](https://www-scopus-com.ezproxy.lib.swin.edu.au/sourceid/19532?origin=resultslist), 59, pp. 18-25

# [Appalachian versus non-Appalachian U.S. traffic fatalities, 2008-2010](https://www-scopus-com.ezproxy.lib.swin.edu.au/record/display.uri?eid=2-s2.0-84878010929&origin=resultslist&sort=plf-f&src=s&st1=%28predict*+OR+model%29+AND+%28%22serious+crash*%22+OR+%22serious+accident%22+OR+%22serious+collision%22+OR+%22fatal+accident%22+OR+%22fatal+crash%22+OR+%22fatal+collision%22+OR+%22road+deaths%22+OR+%22road+fatal*%22+OR+%22traffic+fatal*%22+OR+%22collision+fatal*%22+OR+%22accident+fatal*%22%29+AND+%28driver%29+&nlo=&nlr=&nls=&sid=34fc91d90b0a1eb0b79c181c6b17236b&sot=b&sdt=b&sl=290&s=TITLE-ABS-KEY%28%28predict*+OR+model%29+AND+%28%22serious+crash*%22+OR+%22serious+accident%22+OR+%22serious+collision%22+OR+%22fatal+accident%22+OR+%22fatal+crash%22+OR+%22fatal+collision%22+OR+%22road+deaths%22+OR+%22road+fatal*%22+OR+%22traffic+fatal*%22+OR+%22collision+fatal*%22+OR+%22accident+fatal*%22%29+AND+%28driver%29+%29+AND+PUBYEAR+%3e+1983&relpos=229&citeCnt=3&searchTerm=)

[Zhu, M.](https://www-scopus-com.ezproxy.lib.swin.edu.au/authid/detail.uri?origin=resultslist&authorId=55459580800&zone=), [Zhao, S.](https://www-scopus-com.ezproxy.lib.swin.edu.au/authid/detail.uri?origin=resultslist&authorId=55503276800&zone=), [Gurka, K.K.](https://www-scopus-com.ezproxy.lib.swin.edu.au/authid/detail.uri?origin=resultslist&authorId=8146755000&zone=), [Kandati, S.](https://www-scopus-com.ezproxy.lib.swin.edu.au/authid/detail.uri?origin=resultslist&authorId=55654489900&zone=), [Coben, J.H.](https://www-scopus-com.ezproxy.lib.swin.edu.au/authid/detail.uri?origin=resultslist&authorId=7003630304&zone=) 2013, [Annals of Epidemiology](https://www-scopus-com.ezproxy.lib.swin.edu.au/sourceid/19569?origin=resultslist), 23(6), pp. 377-380

# [Graduated driver licensing program component calibrations and their association with fatal crash involvement](https://www-scopus-com.ezproxy.lib.swin.edu.au/record/display.uri?eid=2-s2.0-84877789274&origin=resultslist&sort=plf-f&src=s&st1=%28predict*+OR+model%29+AND+%28%22serious+crash*%22+OR+%22serious+accident%22+OR+%22serious+collision%22+OR+%22fatal+accident%22+OR+%22fatal+crash%22+OR+%22fatal+collision%22+OR+%22road+deaths%22+OR+%22road+fatal*%22+OR+%22traffic+fatal*%22+OR+%22collision+fatal*%22+OR+%22accident+fatal*%22%29+AND+%28driver%29+&nlo=&nlr=&nls=&sid=34fc91d90b0a1eb0b79c181c6b17236b&sot=b&sdt=b&sl=290&s=TITLE-ABS-KEY%28%28predict*+OR+model%29+AND+%28%22serious+crash*%22+OR+%22serious+accident%22+OR+%22serious+collision%22+OR+%22fatal+accident%22+OR+%22fatal+crash%22+OR+%22fatal+collision%22+OR+%22road+deaths%22+OR+%22road+fatal*%22+OR+%22traffic+fatal*%22+OR+%22collision+fatal*%22+OR+%22accident+fatal*%22%29+AND+%28driver%29+%29+AND+PUBYEAR+%3e+1983&relpos=230&citeCnt=33&searchTerm=)

[Masten, S.V.](https://www-scopus-com.ezproxy.lib.swin.edu.au/authid/detail.uri?origin=resultslist&authorId=15849045600&zone=), [Foss, R.D.](https://www-scopus-com.ezproxy.lib.swin.edu.au/authid/detail.uri?origin=resultslist&authorId=7005104787&zone=), [Marshall, S.W.](https://www-scopus-com.ezproxy.lib.swin.edu.au/authid/detail.uri?origin=resultslist&authorId=7401823263&zone=) 2013, [Accident Analysis and Prevention](https://www-scopus-com.ezproxy.lib.swin.edu.au/sourceid/19532?origin=resultslist), 57, pp. 105-113

# [The influence of stimulants on truck driver crash responsibility in fatal crashes](https://www-scopus-com.ezproxy.lib.swin.edu.au/record/display.uri?eid=2-s2.0-84876478829&origin=resultslist&sort=plf-f&src=s&st1=%28predict*+OR+model%29+AND+%28%22serious+crash*%22+OR+%22serious+accident%22+OR+%22serious+collision%22+OR+%22fatal+accident%22+OR+%22fatal+crash%22+OR+%22fatal+collision%22+OR+%22road+deaths%22+OR+%22road+fatal*%22+OR+%22traffic+fatal*%22+OR+%22collision+fatal*%22+OR+%22accident+fatal*%22%29+AND+%28driver%29+&nlo=&nlr=&nls=&sid=34fc91d90b0a1eb0b79c181c6b17236b&sot=b&sdt=b&sl=290&s=TITLE-ABS-KEY%28%28predict*+OR+model%29+AND+%28%22serious+crash*%22+OR+%22serious+accident%22+OR+%22serious+collision%22+OR+%22fatal+accident%22+OR+%22fatal+crash%22+OR+%22fatal+collision%22+OR+%22road+deaths%22+OR+%22road+fatal*%22+OR+%22traffic+fatal*%22+OR+%22collision+fatal*%22+OR+%22accident+fatal*%22%29+AND+%28driver%29+%29+AND+PUBYEAR+%3e+1983&relpos=231&citeCnt=21&searchTerm=)

[Gates, J.](https://www-scopus-com.ezproxy.lib.swin.edu.au/authid/detail.uri?origin=resultslist&authorId=55657844600&zone=), [Dubois, S.](https://www-scopus-com.ezproxy.lib.swin.edu.au/authid/detail.uri?origin=resultslist&authorId=7005784023&zone=), [Mullen, N.](https://www-scopus-com.ezproxy.lib.swin.edu.au/authid/detail.uri?origin=resultslist&authorId=24402570600&zone=), [Weaver, B.](https://www-scopus-com.ezproxy.lib.swin.edu.au/authid/detail.uri?origin=resultslist&authorId=7102533570&zone=), [Bédard, M.](https://www-scopus-com.ezproxy.lib.swin.edu.au/authid/detail.uri?origin=resultslist&authorId=35594547000&zone=) 2013, [Forensic Science International](https://www-scopus-com.ezproxy.lib.swin.edu.au/sourceid/27743?origin=resultslist), 228(1-3), pp. 15-20

# [The interurban DRAG-Spain model: The main factors of influence on road accidents in Spain](https://www-scopus-com.ezproxy.lib.swin.edu.au/record/display.uri?eid=2-s2.0-84867747508&origin=resultslist&sort=plf-f&src=s&st1=%28predict*+OR+model%29+AND+%28%22serious+crash*%22+OR+%22serious+accident%22+OR+%22serious+collision%22+OR+%22fatal+accident%22+OR+%22fatal+crash%22+OR+%22fatal+collision%22+OR+%22road+deaths%22+OR+%22road+fatal*%22+OR+%22traffic+fatal*%22+OR+%22collision+fatal*%22+OR+%22accident+fatal*%22%29+AND+%28driver%29+&nlo=&nlr=&nls=&sid=34fc91d90b0a1eb0b79c181c6b17236b&sot=b&sdt=b&sl=290&s=TITLE-ABS-KEY%28%28predict*+OR+model%29+AND+%28%22serious+crash*%22+OR+%22serious+accident%22+OR+%22serious+collision%22+OR+%22fatal+accident%22+OR+%22fatal+crash%22+OR+%22fatal+collision%22+OR+%22road+deaths%22+OR+%22road+fatal*%22+OR+%22traffic+fatal*%22+OR+%22collision+fatal*%22+OR+%22accident+fatal*%22%29+AND+%28driver%29+%29+AND+PUBYEAR+%3e+1983&relpos=244&citeCnt=7&searchTerm=)

[Aparicio Izquierdo, F.](https://www-scopus-com.ezproxy.lib.swin.edu.au/authid/detail.uri?origin=resultslist&authorId=36008027700&zone=), [Arenas Ramírez, B.](https://www-scopus-com.ezproxy.lib.swin.edu.au/authid/detail.uri?origin=resultslist&authorId=37000643800&zone=), [Bernardos Rodríguez, E.](https://www-scopus-com.ezproxy.lib.swin.edu.au/authid/detail.uri?origin=resultslist&authorId=36550210400&zone=) 2013, [Research in Transportation Economics](https://www-scopus-com.ezproxy.lib.swin.edu.au/sourceid/4100151536?origin=resultslist), 37(1), pp. 57-65

# [A macro-analysis of impacts of safety interventions on road safety developments in a country](https://www-scopus-com.ezproxy.lib.swin.edu.au/record/display.uri?eid=2-s2.0-84892089904&origin=resultslist&sort=plf-f&src=s&st1=%28predict*+OR+model%29+AND+%28%22serious+crash*%22+OR+%22serious+accident%22+OR+%22serious+collision%22+OR+%22fatal+accident%22+OR+%22fatal+crash%22+OR+%22fatal+collision%22+OR+%22road+deaths%22+OR+%22road+fatal*%22+OR+%22traffic+fatal*%22+OR+%22collision+fatal*%22+OR+%22accident+fatal*%22%29+AND+%28driver%29+&nlo=&nlr=&nls=&sid=34fc91d90b0a1eb0b79c181c6b17236b&sot=b&sdt=b&sl=290&s=TITLE-ABS-KEY%28%28predict*+OR+model%29+AND+%28%22serious+crash*%22+OR+%22serious+accident%22+OR+%22serious+collision%22+OR+%22fatal+accident%22+OR+%22fatal+crash%22+OR+%22fatal+collision%22+OR+%22road+deaths%22+OR+%22road+fatal*%22+OR+%22traffic+fatal*%22+OR+%22collision+fatal*%22+OR+%22accident+fatal*%22%29+AND+%28driver%29+%29+AND+PUBYEAR+%3e+1983&relpos=248&citeCnt=0&searchTerm=) (Book Chapter)

[Gitelman, V.](https://www-scopus-com.ezproxy.lib.swin.edu.au/authid/detail.uri?origin=resultslist&authorId=6602577738&zone=), [Doveh, E.](https://www-scopus-com.ezproxy.lib.swin.edu.au/authid/detail.uri?origin=resultslist&authorId=6507612001&zone=), [Hendel, L.](https://www-scopus-com.ezproxy.lib.swin.edu.au/authid/detail.uri?origin=resultslist&authorId=36677553700&zone=), [Bekhor, S.](https://www-scopus-com.ezproxy.lib.swin.edu.au/authid/detail.uri?origin=resultslist&authorId=6602561719&zone=) 2012, [*Accidents: Risk Factors, Health Outcomes and Safety Measures*](https://www-scopus-com.ezproxy.lib.swin.edu.au/display/book.uri?sourceId=21100280152&requestedPage=book)

pp. 139-170

# [Young unlicensed drivers and passenger safety restraint use in U.S. fatal crashes: Concern for risk spillover effect?](https://www-scopus-com.ezproxy.lib.swin.edu.au/record/display.uri?eid=2-s2.0-84876722405&origin=resultslist&sort=plf-f&src=s&st1=%28predict*+OR+model%29+AND+%28%22serious+crash*%22+OR+%22serious+accident%22+OR+%22serious+collision%22+OR+%22fatal+accident%22+OR+%22fatal+crash%22+OR+%22fatal+collision%22+OR+%22road+deaths%22+OR+%22road+fatal*%22+OR+%22traffic+fatal*%22+OR+%22collision+fatal*%22+OR+%22accident+fatal*%22%29+AND+%28driver%29+&nlo=&nlr=&nls=&sid=34fc91d90b0a1eb0b79c181c6b17236b&sot=b&sdt=b&sl=290&s=TITLE-ABS-KEY%28%28predict*+OR+model%29+AND+%28%22serious+crash*%22+OR+%22serious+accident%22+OR+%22serious+collision%22+OR+%22fatal+accident%22+OR+%22fatal+crash%22+OR+%22fatal+collision%22+OR+%22road+deaths%22+OR+%22road+fatal*%22+OR+%22traffic+fatal*%22+OR+%22collision+fatal*%22+OR+%22accident+fatal*%22%29+AND+%28driver%29+%29+AND+PUBYEAR+%3e+1983&relpos=249&citeCnt=3&searchTerm=)

[Fu, J.](https://www-scopus-com.ezproxy.lib.swin.edu.au/authid/detail.uri?origin=resultslist&authorId=54419860900&zone=), [Anderson, C.L.](https://www-scopus-com.ezproxy.lib.swin.edu.au/authid/detail.uri?origin=resultslist&authorId=35556095500&zone=), [Dziura, J.D.](https://www-scopus-com.ezproxy.lib.swin.edu.au/authid/detail.uri?origin=resultslist&authorId=6602746173&zone=), [Crowley, M.J.](https://www-scopus-com.ezproxy.lib.swin.edu.au/authid/detail.uri?origin=resultslist&authorId=7103054032&zone=), [Vaca, F.E.](https://www-scopus-com.ezproxy.lib.swin.edu.au/authid/detail.uri?origin=resultslist&authorId=7003561651&zone=) 2012, [Annals of Advances in Automotive Medicine](https://www-scopus-com.ezproxy.lib.swin.edu.au/sourceid/19700188374?origin=resultslist), 56, pp. 37-43

# [The role of sensation seeking, perceived peer pressure, and harmful alcohol use in riding with an alcohol-impaired driver](https://www-scopus-com.ezproxy.lib.swin.edu.au/record/display.uri?eid=2-s2.0-84862806236&origin=resultslist&sort=plf-f&src=s&st1=%28predict*+OR+model%29+AND+%28%22serious+crash*%22+OR+%22serious+accident%22+OR+%22serious+collision%22+OR+%22fatal+accident%22+OR+%22fatal+crash%22+OR+%22fatal+collision%22+OR+%22road+deaths%22+OR+%22road+fatal*%22+OR+%22traffic+fatal*%22+OR+%22collision+fatal*%22+OR+%22accident+fatal*%22%29+AND+%28driver%29+&nlo=&nlr=&nls=&sid=34fc91d90b0a1eb0b79c181c6b17236b&sot=b&sdt=b&sl=290&s=TITLE-ABS-KEY%28%28predict*+OR+model%29+AND+%28%22serious+crash*%22+OR+%22serious+accident%22+OR+%22serious+collision%22+OR+%22fatal+accident%22+OR+%22fatal+crash%22+OR+%22fatal+collision%22+OR+%22road+deaths%22+OR+%22road+fatal*%22+OR+%22traffic+fatal*%22+OR+%22collision+fatal*%22+OR+%22accident+fatal*%22%29+AND+%28driver%29+%29+AND+PUBYEAR+%3e+1983&relpos=256&citeCnt=9&searchTerm=)

[Kim, J.-H.](https://www-scopus-com.ezproxy.lib.swin.edu.au/authid/detail.uri?origin=resultslist&authorId=39361601000&zone=), [Kim, K.S.](https://www-scopus-com.ezproxy.lib.swin.edu.au/authid/detail.uri?origin=resultslist&authorId=57188998598&zone=) 2012, [Accident Analysis and Prevention](https://www-scopus-com.ezproxy.lib.swin.edu.au/sourceid/19532?origin=resultslist), 48, pp. 326-334

# [Analysis of driver injury severity in rural single-vehicle crashes](https://www-scopus-com.ezproxy.lib.swin.edu.au/record/display.uri?eid=2-s2.0-84856434659&origin=resultslist&sort=plf-f&src=s&st1=%28predict*+OR+model%29+AND+%28%22serious+crash*%22+OR+%22serious+accident%22+OR+%22serious+collision%22+OR+%22fatal+accident%22+OR+%22fatal+crash%22+OR+%22fatal+collision%22+OR+%22road+deaths%22+OR+%22road+fatal*%22+OR+%22traffic+fatal*%22+OR+%22collision+fatal*%22+OR+%22accident+fatal*%22%29+AND+%28driver%29+&nlo=&nlr=&nls=&sid=34fc91d90b0a1eb0b79c181c6b17236b&sot=b&sdt=b&sl=290&s=TITLE-ABS-KEY%28%28predict*+OR+model%29+AND+%28%22serious+crash*%22+OR+%22serious+accident%22+OR+%22serious+collision%22+OR+%22fatal+accident%22+OR+%22fatal+crash%22+OR+%22fatal+collision%22+OR+%22road+deaths%22+OR+%22road+fatal*%22+OR+%22traffic+fatal*%22+OR+%22collision+fatal*%22+OR+%22accident+fatal*%22%29+AND+%28driver%29+%29+AND+PUBYEAR+%3e+1983&relpos=259&citeCnt=64&searchTerm=)

[Xie, Y.](https://www-scopus-com.ezproxy.lib.swin.edu.au/authid/detail.uri?origin=resultslist&authorId=21234379500&zone=), [Zhao, K.](https://www-scopus-com.ezproxy.lib.swin.edu.au/authid/detail.uri?origin=resultslist&authorId=57198258750&zone=), [Huynh, N.](https://www-scopus-com.ezproxy.lib.swin.edu.au/authid/detail.uri?origin=resultslist&authorId=7004216909&zone=) 2012, [Accident Analysis and Prevention](https://www-scopus-com.ezproxy.lib.swin.edu.au/sourceid/19532?origin=resultslist), 47, pp. 36-44

# [The effectiveness of alcohol control policies on alcohol-related traffic fatalities in the United States](https://www-scopus-com.ezproxy.lib.swin.edu.au/record/display.uri?eid=2-s2.0-84856110169&origin=resultslist&sort=plf-f&src=s&st1=%28predict*+OR+model%29+AND+%28%22serious+crash*%22+OR+%22serious+accident%22+OR+%22serious+collision%22+OR+%22fatal+accident%22+OR+%22fatal+crash%22+OR+%22fatal+collision%22+OR+%22road+deaths%22+OR+%22road+fatal*%22+OR+%22traffic+fatal*%22+OR+%22collision+fatal*%22+OR+%22accident+fatal*%22%29+AND+%28driver%29+&nlo=&nlr=&nls=&sid=34fc91d90b0a1eb0b79c181c6b17236b&sot=b&sdt=b&sl=290&s=TITLE-ABS-KEY%28%28predict*+OR+model%29+AND+%28%22serious+crash*%22+OR+%22serious+accident%22+OR+%22serious+collision%22+OR+%22fatal+accident%22+OR+%22fatal+crash%22+OR+%22fatal+collision%22+OR+%22road+deaths%22+OR+%22road+fatal*%22+OR+%22traffic+fatal*%22+OR+%22collision+fatal*%22+OR+%22accident+fatal*%22%29+AND+%28driver%29+%29+AND+PUBYEAR+%3e+1983&relpos=263&citeCnt=31&searchTerm=)

[Chang, K.](https://www-scopus-com.ezproxy.lib.swin.edu.au/authid/detail.uri?origin=resultslist&authorId=8527358800&zone=), [Wu, C.-C.](https://www-scopus-com.ezproxy.lib.swin.edu.au/authid/detail.uri?origin=resultslist&authorId=50263693500&zone=), [Ying, Y.-H.](https://www-scopus-com.ezproxy.lib.swin.edu.au/authid/detail.uri?origin=resultslist&authorId=7102459055&zone=) 2012, [Accident Analysis and Prevention](https://www-scopus-com.ezproxy.lib.swin.edu.au/sourceid/19532?origin=resultslist), 45, pp. 406-415

# [Reducing the legal blood alcohol concentration limit for driving in developing countries: A time for change? Results and implications derived from a time-series analysis (2001-10) conducted in Brazil](https://www-scopus-com.ezproxy.lib.swin.edu.au/record/display.uri?eid=2-s2.0-80455164600&origin=resultslist&sort=plf-f&src=s&st1=%28predict*+OR+model%29+AND+%28%22serious+crash*%22+OR+%22serious+accident%22+OR+%22serious+collision%22+OR+%22fatal+accident%22+OR+%22fatal+crash%22+OR+%22fatal+collision%22+OR+%22road+deaths%22+OR+%22road+fatal*%22+OR+%22traffic+fatal*%22+OR+%22collision+fatal*%22+OR+%22accident+fatal*%22%29+AND+%28driver%29+&nlo=&nlr=&nls=&sid=34fc91d90b0a1eb0b79c181c6b17236b&sot=b&sdt=b&sl=290&s=TITLE-ABS-KEY%28%28predict*+OR+model%29+AND+%28%22serious+crash*%22+OR+%22serious+accident%22+OR+%22serious+collision%22+OR+%22fatal+accident%22+OR+%22fatal+crash%22+OR+%22fatal+collision%22+OR+%22road+deaths%22+OR+%22road+fatal*%22+OR+%22traffic+fatal*%22+OR+%22collision+fatal*%22+OR+%22accident+fatal*%22%29+AND+%28driver%29+%29+AND+PUBYEAR+%3e+1983&relpos=277&citeCnt=53&searchTerm=)

[Andreuccetti, G.](https://www-scopus-com.ezproxy.lib.swin.edu.au/authid/detail.uri?origin=resultslist&authorId=24586967200&zone=), [Carvalho, H.B.](https://www-scopus-com.ezproxy.lib.swin.edu.au/authid/detail.uri?origin=resultslist&authorId=55662840700&zone=), [Cherpitel, C.J.](https://www-scopus-com.ezproxy.lib.swin.edu.au/authid/detail.uri?origin=resultslist&authorId=7005582827&zone=), (...), [Kahn, T.](https://www-scopus-com.ezproxy.lib.swin.edu.au/authid/detail.uri?origin=resultslist&authorId=8928878800&zone=), [Leyton, V.](https://www-scopus-com.ezproxy.lib.swin.edu.au/authid/detail.uri?origin=resultslist&authorId=24587055900&zone=) 2011, [Addiction](https://www-scopus-com.ezproxy.lib.swin.edu.au/sourceid/24745?origin=resultslist), 106(12), pp. 2124-2131

# [Vulnerability of female drivers involved in motor vehicle crashes: An analysis of US population at risk](https://www-scopus-com.ezproxy.lib.swin.edu.au/record/display.uri?eid=2-s2.0-81555202926&origin=resultslist&sort=plf-f&src=s&st1=%28predict*+OR+model%29+AND+%28%22serious+crash*%22+OR+%22serious+accident%22+OR+%22serious+collision%22+OR+%22fatal+accident%22+OR+%22fatal+crash%22+OR+%22fatal+collision%22+OR+%22road+deaths%22+OR+%22road+fatal*%22+OR+%22traffic+fatal*%22+OR+%22collision+fatal*%22+OR+%22accident+fatal*%22%29+AND+%28driver%29+&nlo=&nlr=&nls=&sid=34fc91d90b0a1eb0b79c181c6b17236b&sot=b&sdt=b&sl=290&s=TITLE-ABS-KEY%28%28predict*+OR+model%29+AND+%28%22serious+crash*%22+OR+%22serious+accident%22+OR+%22serious+collision%22+OR+%22fatal+accident%22+OR+%22fatal+crash%22+OR+%22fatal+collision%22+OR+%22road+deaths%22+OR+%22road+fatal*%22+OR+%22traffic+fatal*%22+OR+%22collision+fatal*%22+OR+%22accident+fatal*%22%29+AND+%28driver%29+%29+AND+PUBYEAR+%3e+1983&relpos=280&citeCnt=48&searchTerm=)

[Bose, D.](https://www-scopus-com.ezproxy.lib.swin.edu.au/authid/detail.uri?origin=resultslist&authorId=57205255835&zone=), [Segui-Gomez, M.](https://www-scopus-com.ezproxy.lib.swin.edu.au/authid/detail.uri?origin=resultslist&authorId=7003779293&zone=), [Crandall, J.R.](https://www-scopus-com.ezproxy.lib.swin.edu.au/authid/detail.uri?origin=resultslist&authorId=7004904321&zone=) 2011, [American Journal of Public Health](https://www-scopus-com.ezproxy.lib.swin.edu.au/sourceid/19561?origin=resultslist), 101(12), pp. 2368-2373

# [Type of Motor Carrier and driver history in fatal bus crashes](https://www-scopus-com.ezproxy.lib.swin.edu.au/record/display.uri?eid=2-s2.0-79951524224&origin=resultslist&sort=plf-f&src=s&st1=%28predict*+OR+model%29+AND+%28%22serious+crash*%22+OR+%22serious+accident%22+OR+%22serious+collision%22+OR+%22fatal+accident%22+OR+%22fatal+crash%22+OR+%22fatal+collision%22+OR+%22road+deaths%22+OR+%22road+fatal*%22+OR+%22traffic+fatal*%22+OR+%22collision+fatal*%22+OR+%22accident+fatal*%22%29+AND+%28driver%29+&nlo=&nlr=&nls=&sid=34fc91d90b0a1eb0b79c181c6b17236b&sot=b&sdt=b&sl=290&s=TITLE-ABS-KEY%28%28predict*+OR+model%29+AND+%28%22serious+crash*%22+OR+%22serious+accident%22+OR+%22serious+collision%22+OR+%22fatal+accident%22+OR+%22fatal+crash%22+OR+%22fatal+collision%22+OR+%22road+deaths%22+OR+%22road+fatal*%22+OR+%22traffic+fatal*%22+OR+%22collision+fatal*%22+OR+%22accident+fatal*%22%29+AND+%28driver%29+%29+AND+PUBYEAR+%3e+1983&relpos=297&citeCnt=16&searchTerm=)

[Blower, D.](https://www-scopus-com.ezproxy.lib.swin.edu.au/authid/detail.uri?origin=resultslist&authorId=8844546000&zone=), [Green, P.E.](https://www-scopus-com.ezproxy.lib.swin.edu.au/authid/detail.uri?origin=resultslist&authorId=36999771600&zone=) 2010, [Transportation Research Record](https://www-scopus-com.ezproxy.lib.swin.edu.au/sourceid/27418?origin=resultslist), (2194), pp. 37-43

# [Trends in fatalities from distracted driving in the United States, 1999 to 2008](https://www-scopus-com.ezproxy.lib.swin.edu.au/record/display.uri?eid=2-s2.0-77958186631&origin=resultslist&sort=plf-f&src=s&st1=%28predict*+OR+model%29+AND+%28%22serious+crash*%22+OR+%22serious+accident%22+OR+%22serious+collision%22+OR+%22fatal+accident%22+OR+%22fatal+crash%22+OR+%22fatal+collision%22+OR+%22road+deaths%22+OR+%22road+fatal*%22+OR+%22traffic+fatal*%22+OR+%22collision+fatal*%22+OR+%22accident+fatal*%22%29+AND+%28driver%29+&nlo=&nlr=&nls=&sid=34fc91d90b0a1eb0b79c181c6b17236b&sot=b&sdt=b&sl=290&s=TITLE-ABS-KEY%28%28predict*+OR+model%29+AND+%28%22serious+crash*%22+OR+%22serious+accident%22+OR+%22serious+collision%22+OR+%22fatal+accident%22+OR+%22fatal+crash%22+OR+%22fatal+collision%22+OR+%22road+deaths%22+OR+%22road+fatal*%22+OR+%22traffic+fatal*%22+OR+%22collision+fatal*%22+OR+%22accident+fatal*%22%29+AND+%28driver%29+%29+AND+PUBYEAR+%3e+1983&relpos=300&citeCnt=163&searchTerm=)

[Wilson, F.A.](https://www-scopus-com.ezproxy.lib.swin.edu.au/authid/detail.uri?origin=resultslist&authorId=55648733100&zone=), [Stimpson, J.P.](https://www-scopus-com.ezproxy.lib.swin.edu.au/authid/detail.uri?origin=resultslist&authorId=12771406500&zone=) 2010. [American Journal of Public Health](https://www-scopus-com.ezproxy.lib.swin.edu.au/sourceid/19561?origin=resultslist), 100(11), pp. 2213-2219

# [The impact of traffic violations on the estimated cost of traffic accidents with victims](https://www-scopus-com.ezproxy.lib.swin.edu.au/record/display.uri?eid=2-s2.0-76049121396&origin=resultslist&sort=plf-f&src=s&st1=%28predict*+OR+model%29+AND+%28%22serious+crash*%22+OR+%22serious+accident%22+OR+%22serious+collision%22+OR+%22fatal+accident%22+OR+%22fatal+crash%22+OR+%22fatal+collision%22+OR+%22road+deaths%22+OR+%22road+fatal*%22+OR+%22traffic+fatal*%22+OR+%22collision+fatal*%22+OR+%22accident+fatal*%22%29+AND+%28driver%29+&nlo=&nlr=&nls=&sid=34fc91d90b0a1eb0b79c181c6b17236b&sot=b&sdt=b&sl=290&s=TITLE-ABS-KEY%28%28predict*+OR+model%29+AND+%28%22serious+crash*%22+OR+%22serious+accident%22+OR+%22serious+collision%22+OR+%22fatal+accident%22+OR+%22fatal+crash%22+OR+%22fatal+collision%22+OR+%22road+deaths%22+OR+%22road+fatal*%22+OR+%22traffic+fatal*%22+OR+%22collision+fatal*%22+OR+%22accident+fatal*%22%29+AND+%28driver%29+%29+AND+PUBYEAR+%3e+1983&relpos=305&citeCnt=30&searchTerm=)

[Ayuso, M.](https://www-scopus-com.ezproxy.lib.swin.edu.au/authid/detail.uri?origin=resultslist&authorId=36779920700&zone=), [Guillén, M.](https://www-scopus-com.ezproxy.lib.swin.edu.au/authid/detail.uri?origin=resultslist&authorId=7103020782&zone=), [Alcañiz, M.](https://www-scopus-com.ezproxy.lib.swin.edu.au/authid/detail.uri?origin=resultslist&authorId=35557971500&zone=) 2010, [Accident Analysis and Prevention](https://www-scopus-com.ezproxy.lib.swin.edu.au/sourceid/19532?origin=resultslist), 42(2), pp. 709-717

# [The association between opioid analgesics and unsafe driving actions preceding fatal crashes](https://www-scopus-com.ezproxy.lib.swin.edu.au/record/display.uri?eid=2-s2.0-71549144414&origin=resultslist&sort=plf-f&src=s&st1=%28predict*+OR+model%29+AND+%28%22serious+crash*%22+OR+%22serious+accident%22+OR+%22serious+collision%22+OR+%22fatal+accident%22+OR+%22fatal+crash%22+OR+%22fatal+collision%22+OR+%22road+deaths%22+OR+%22road+fatal*%22+OR+%22traffic+fatal*%22+OR+%22collision+fatal*%22+OR+%22accident+fatal*%22%29+AND+%28driver%29+&nlo=&nlr=&nls=&sid=34fc91d90b0a1eb0b79c181c6b17236b&sot=b&sdt=b&sl=290&s=TITLE-ABS-KEY%28%28predict*+OR+model%29+AND+%28%22serious+crash*%22+OR+%22serious+accident%22+OR+%22serious+collision%22+OR+%22fatal+accident%22+OR+%22fatal+crash%22+OR+%22fatal+collision%22+OR+%22road+deaths%22+OR+%22road+fatal*%22+OR+%22traffic+fatal*%22+OR+%22collision+fatal*%22+OR+%22accident+fatal*%22%29+AND+%28driver%29+%29+AND+PUBYEAR+%3e+1983&relpos=308&citeCnt=38&searchTerm=)

[Dubois, S.](https://www-scopus-com.ezproxy.lib.swin.edu.au/authid/detail.uri?origin=resultslist&authorId=7005784023&zone=), [Bédard, M.](https://www-scopus-com.ezproxy.lib.swin.edu.au/authid/detail.uri?origin=resultslist&authorId=35594547000&zone=), [Weaver, B.](https://www-scopus-com.ezproxy.lib.swin.edu.au/authid/detail.uri?origin=resultslist&authorId=7102533570&zone=) 2010, [Accident Analysis and Prevention](https://www-scopus-com.ezproxy.lib.swin.edu.au/sourceid/19532?origin=resultslist), 42(1), pp. 30-37

# [Female involvement in U.S. nonfatal crashes under a three-level hierarchical crash model](https://www-scopus-com.ezproxy.lib.swin.edu.au/record/display.uri?eid=2-s2.0-78049409417&origin=resultslist&sort=plf-f&src=s&st1=%28predict*+OR+model%29+AND+%28%22serious+crash*%22+OR+%22serious+accident%22+OR+%22serious+collision%22+OR+%22fatal+accident%22+OR+%22fatal+crash%22+OR+%22fatal+collision%22+OR+%22road+deaths%22+OR+%22road+fatal*%22+OR+%22traffic+fatal*%22+OR+%22collision+fatal*%22+OR+%22accident+fatal*%22%29+AND+%28driver%29+&nlo=&nlr=&nls=&sid=34fc91d90b0a1eb0b79c181c6b17236b&sot=b&sdt=b&sl=290&s=TITLE-ABS-KEY%28%28predict*+OR+model%29+AND+%28%22serious+crash*%22+OR+%22serious+accident%22+OR+%22serious+collision%22+OR+%22fatal+accident%22+OR+%22fatal+crash%22+OR+%22fatal+collision%22+OR+%22road+deaths%22+OR+%22road+fatal*%22+OR+%22traffic+fatal*%22+OR+%22collision+fatal*%22+OR+%22accident+fatal*%22%29+AND+%28driver%29+%29+AND+PUBYEAR+%3e+1983&relpos=311&citeCnt=14&searchTerm=)

[Kelley-Baker, T.](https://www-scopus-com.ezproxy.lib.swin.edu.au/authid/detail.uri?origin=resultslist&authorId=12779383600&zone=), [Romano, E.](https://www-scopus-com.ezproxy.lib.swin.edu.au/authid/detail.uri?origin=resultslist&authorId=7102438608&zone=) 2010, [Accident Analysis and Prevention](https://www-scopus-com.ezproxy.lib.swin.edu.au/sourceid/19532?origin=resultslist), 42(6), pp. 2007-2012

# [Fatal crash trends for Australian young drivers 1997-2007: Geographic and socioeconomic differentials](https://www-scopus-com.ezproxy.lib.swin.edu.au/record/display.uri?eid=2-s2.0-83555173694&origin=resultslist&sort=plf-f&src=s&st1=%28predict*+OR+model%29+AND+%28%22serious+crash*%22+OR+%22serious+accident%22+OR+%22serious+collision%22+OR+%22fatal+accident%22+OR+%22fatal+crash%22+OR+%22fatal+collision%22+OR+%22road+deaths%22+OR+%22road+fatal*%22+OR+%22traffic+fatal*%22+OR+%22collision+fatal*%22+OR+%22accident+fatal*%22%29+AND+%28driver%29+&nlo=&nlr=&nls=&sid=34fc91d90b0a1eb0b79c181c6b17236b&sot=b&sdt=b&sl=290&s=TITLE-ABS-KEY%28%28predict*+OR+model%29+AND+%28%22serious+crash*%22+OR+%22serious+accident%22+OR+%22serious+collision%22+OR+%22fatal+accident%22+OR+%22fatal+crash%22+OR+%22fatal+collision%22+OR+%22road+deaths%22+OR+%22road+fatal*%22+OR+%22traffic+fatal*%22+OR+%22collision+fatal*%22+OR+%22accident+fatal*%22%29+AND+%28driver%29+%29+AND+PUBYEAR+%3e+1983&relpos=312&citeCnt=22&searchTerm=)

[Chen, H.Y.](https://www-scopus-com.ezproxy.lib.swin.edu.au/authid/detail.uri?origin=resultslist&authorId=35204833800&zone=), [Senserrick, T.](https://www-scopus-com.ezproxy.lib.swin.edu.au/authid/detail.uri?origin=resultslist&authorId=14036415400&zone=), [Martiniuk, A.L.C.](https://www-scopus-com.ezproxy.lib.swin.edu.au/authid/detail.uri?origin=resultslist&authorId=15835035500&zone=), (...), [Chang, H.Y.](https://www-scopus-com.ezproxy.lib.swin.edu.au/authid/detail.uri?origin=resultslist&authorId=7407523087&zone=), [Norton, R.](https://www-scopus-com.ezproxy.lib.swin.edu.au/authid/detail.uri?origin=resultslist&authorId=56253453500&zone=) 2010, [Journal of Safety Research](https://www-scopus-com.ezproxy.lib.swin.edu.au/sourceid/29284?origin=resultslist), 41(2), pp. 123-128

# [Poverty as a determinant of young drivers' fatal crash risks](https://www-scopus-com.ezproxy.lib.swin.edu.au/record/display.uri?eid=2-s2.0-71649083348&origin=resultslist&sort=plf-f&src=s&st1=%28predict*+OR+model%29+AND+%28%22serious+crash*%22+OR+%22serious+accident%22+OR+%22serious+collision%22+OR+%22fatal+accident%22+OR+%22fatal+crash%22+OR+%22fatal+collision%22+OR+%22road+deaths%22+OR+%22road+fatal*%22+OR+%22traffic+fatal*%22+OR+%22collision+fatal*%22+OR+%22accident+fatal*%22%29+AND+%28driver%29+&nlo=&nlr=&nls=&sid=34fc91d90b0a1eb0b79c181c6b17236b&sot=b&sdt=b&sl=290&s=TITLE-ABS-KEY%28%28predict*+OR+model%29+AND+%28%22serious+crash*%22+OR+%22serious+accident%22+OR+%22serious+collision%22+OR+%22fatal+accident%22+OR+%22fatal+crash%22+OR+%22fatal+collision%22+OR+%22road+deaths%22+OR+%22road+fatal*%22+OR+%22traffic+fatal*%22+OR+%22collision+fatal*%22+OR+%22accident+fatal*%22%29+AND+%28driver%29+%29+AND+PUBYEAR+%3e+1983&relpos=316&citeCnt=24&searchTerm=)

[Males, M.A.](https://www-scopus-com.ezproxy.lib.swin.edu.au/authid/detail.uri?origin=resultslist&authorId=6701337105&zone=) 2009, [Journal of Safety Research](https://www-scopus-com.ezproxy.lib.swin.edu.au/sourceid/29284?origin=resultslist), 40(6), pp. 443-448

# [Inter-group differences in road-traffic crash involvement](https://www-scopus-com.ezproxy.lib.swin.edu.au/record/display.uri?eid=2-s2.0-55049113532&origin=resultslist&sort=plf-f&src=s&st1=%28predict*+OR+model%29+AND+%28%22serious+crash*%22+OR+%22serious+accident%22+OR+%22serious+collision%22+OR+%22fatal+accident%22+OR+%22fatal+crash%22+OR+%22fatal+collision%22+OR+%22road+deaths%22+OR+%22road+fatal*%22+OR+%22traffic+fatal*%22+OR+%22collision+fatal*%22+OR+%22accident+fatal*%22%29+AND+%28driver%29+&nlo=&nlr=&nls=&sid=34fc91d90b0a1eb0b79c181c6b17236b&sot=b&sdt=b&sl=290&s=TITLE-ABS-KEY%28%28predict*+OR+model%29+AND+%28%22serious+crash*%22+OR+%22serious+accident%22+OR+%22serious+collision%22+OR+%22fatal+accident%22+OR+%22fatal+crash%22+OR+%22fatal+collision%22+OR+%22road+deaths%22+OR+%22road+fatal*%22+OR+%22traffic+fatal*%22+OR+%22collision+fatal*%22+OR+%22accident+fatal*%22%29+AND+%28driver%29+%29+AND+PUBYEAR+%3e+1983&relpos=336&citeCnt=43&searchTerm=)

[Factor, R.](https://www-scopus-com.ezproxy.lib.swin.edu.au/authid/detail.uri?origin=resultslist&authorId=21733667900&zone=), [Mahalel, D.](https://www-scopus-com.ezproxy.lib.swin.edu.au/authid/detail.uri?origin=resultslist&authorId=7004151400&zone=), [Yair, G.](https://www-scopus-com.ezproxy.lib.swin.edu.au/authid/detail.uri?origin=resultslist&authorId=6602765045&zone=) 2008, [Accident Analysis and Prevention](https://www-scopus-com.ezproxy.lib.swin.edu.au/sourceid/19532?origin=resultslist), 40(6), pp. 2000-2007

# [Injury severity analysis of accidents involving young male drivers in Great Britain](https://www-scopus-com.ezproxy.lib.swin.edu.au/record/display.uri?eid=2-s2.0-55749083857&origin=resultslist&sort=plf-f&src=s&st1=%28predict*+OR+model%29+AND+%28%22serious+crash*%22+OR+%22serious+accident%22+OR+%22serious+collision%22+OR+%22fatal+accident%22+OR+%22fatal+crash%22+OR+%22fatal+collision%22+OR+%22road+deaths%22+OR+%22road+fatal*%22+OR+%22traffic+fatal*%22+OR+%22collision+fatal*%22+OR+%22accident+fatal*%22%29+AND+%28driver%29+&nlo=&nlr=&nls=&sid=34fc91d90b0a1eb0b79c181c6b17236b&sot=b&sdt=b&sl=290&s=TITLE-ABS-KEY%28%28predict*+OR+model%29+AND+%28%22serious+crash*%22+OR+%22serious+accident%22+OR+%22serious+collision%22+OR+%22fatal+accident%22+OR+%22fatal+crash%22+OR+%22fatal+collision%22+OR+%22road+deaths%22+OR+%22road+fatal*%22+OR+%22traffic+fatal*%22+OR+%22collision+fatal*%22+OR+%22accident+fatal*%22%29+AND+%28driver%29+%29+AND+PUBYEAR+%3e+1983&relpos=337&citeCnt=66&searchTerm=)

[Gray, R.C.](https://www-scopus-com.ezproxy.lib.swin.edu.au/authid/detail.uri?origin=resultslist&authorId=15769197300&zone=), [Quddus, M.A.](https://www-scopus-com.ezproxy.lib.swin.edu.au/authid/detail.uri?origin=resultslist&authorId=7004336654&zone=), [Evans, A.](https://www-scopus-com.ezproxy.lib.swin.edu.au/authid/detail.uri?origin=resultslist&authorId=7404362710&zone=) 2008, [Journal of Safety Research](https://www-scopus-com.ezproxy.lib.swin.edu.au/sourceid/29284?origin=resultslist), 39(5), pp. 483-495

# [Risk factors for death among older child and teenaged motor vehicle passengers](https://www-scopus-com.ezproxy.lib.swin.edu.au/record/display.uri?eid=2-s2.0-40349102068&origin=resultslist&sort=plf-f&src=s&st1=%28predict*+OR+model%29+AND+%28%22serious+crash*%22+OR+%22serious+accident%22+OR+%22serious+collision%22+OR+%22fatal+accident%22+OR+%22fatal+crash%22+OR+%22fatal+collision%22+OR+%22road+deaths%22+OR+%22road+fatal*%22+OR+%22traffic+fatal*%22+OR+%22collision+fatal*%22+OR+%22accident+fatal*%22%29+AND+%28driver%29+&nlo=&nlr=&nls=&sid=34fc91d90b0a1eb0b79c181c6b17236b&sot=b&sdt=b&sl=290&s=TITLE-ABS-KEY%28%28predict*+OR+model%29+AND+%28%22serious+crash*%22+OR+%22serious+accident%22+OR+%22serious+collision%22+OR+%22fatal+accident%22+OR+%22fatal+crash%22+OR+%22fatal+collision%22+OR+%22road+deaths%22+OR+%22road+fatal*%22+OR+%22traffic+fatal*%22+OR+%22collision+fatal*%22+OR+%22accident+fatal*%22%29+AND+%28driver%29+%29+AND+PUBYEAR+%3e+1983&relpos=342&citeCnt=22&searchTerm=)

[Winston, F.K.](https://www-scopus-com.ezproxy.lib.swin.edu.au/authid/detail.uri?origin=resultslist&authorId=7103265490&zone=), [Kallan, M.J.](https://www-scopus-com.ezproxy.lib.swin.edu.au/authid/detail.uri?origin=resultslist&authorId=6602150952&zone=), [Senserrick, T.M.](https://www-scopus-com.ezproxy.lib.swin.edu.au/authid/detail.uri?origin=resultslist&authorId=14036415400&zone=), [Elliott, M.R.](https://www-scopus-com.ezproxy.lib.swin.edu.au/authid/detail.uri?origin=resultslist&authorId=7402622248&zone=) 2008. [Archives of Pediatrics and Adolescent Medicine](https://www-scopus-com.ezproxy.lib.swin.edu.au/sourceid/14493?origin=resultslist), 162(3), pp. 253-260

# [Regional economic conditions and crash fatality rates - a cross-county analysis](https://www-scopus-com.ezproxy.lib.swin.edu.au/record/display.uri?eid=2-s2.0-39849089018&origin=resultslist&sort=plf-f&src=s&st1=%28predict*+OR+model%29+AND+%28%22serious+crash*%22+OR+%22serious+accident%22+OR+%22serious+collision%22+OR+%22fatal+accident%22+OR+%22fatal+crash%22+OR+%22fatal+collision%22+OR+%22road+deaths%22+OR+%22road+fatal*%22+OR+%22traffic+fatal*%22+OR+%22collision+fatal*%22+OR+%22accident+fatal*%22%29+AND+%28driver%29+&nlo=&nlr=&nls=&sid=34fc91d90b0a1eb0b79c181c6b17236b&sot=b&sdt=b&sl=290&s=TITLE-ABS-KEY%28%28predict*+OR+model%29+AND+%28%22serious+crash*%22+OR+%22serious+accident%22+OR+%22serious+collision%22+OR+%22fatal+accident%22+OR+%22fatal+crash%22+OR+%22fatal+collision%22+OR+%22road+deaths%22+OR+%22road+fatal*%22+OR+%22traffic+fatal*%22+OR+%22collision+fatal*%22+OR+%22accident+fatal*%22%29+AND+%28driver%29+%29+AND+PUBYEAR+%3e+1983&relpos=344&citeCnt=28&searchTerm=)

[Traynor, T.L.](https://www-scopus-com.ezproxy.lib.swin.edu.au/authid/detail.uri?origin=resultslist&authorId=6603704230&zone=) 2008, [Journal of Safety Research](https://www-scopus-com.ezproxy.lib.swin.edu.au/sourceid/29284?origin=resultslist), 39(1), pp. 33-39

# [Young female drivers in fatal crashes: Recent trends, 1995-2004](https://www-scopus-com.ezproxy.lib.swin.edu.au/record/display.uri?eid=2-s2.0-40749125330&origin=resultslist&sort=plf-f&src=s&st1=%28predict*+OR+model%29+AND+%28%22serious+crash*%22+OR+%22serious+accident%22+OR+%22serious+collision%22+OR+%22fatal+accident%22+OR+%22fatal+crash%22+OR+%22fatal+collision%22+OR+%22road+deaths%22+OR+%22road+fatal*%22+OR+%22traffic+fatal*%22+OR+%22collision+fatal*%22+OR+%22accident+fatal*%22%29+AND+%28driver%29+&nlo=&nlr=&nls=&sid=34fc91d90b0a1eb0b79c181c6b17236b&sot=b&sdt=b&sl=290&s=TITLE-ABS-KEY%28%28predict*+OR+model%29+AND+%28%22serious+crash*%22+OR+%22serious+accident%22+OR+%22serious+collision%22+OR+%22fatal+accident%22+OR+%22fatal+crash%22+OR+%22fatal+collision%22+OR+%22road+deaths%22+OR+%22road+fatal*%22+OR+%22traffic+fatal*%22+OR+%22collision+fatal*%22+OR+%22accident+fatal*%22%29+AND+%28driver%29+%29+AND+PUBYEAR+%3e+1983&relpos=348&citeCnt=17&searchTerm=)

[Tsai, V.W.](https://www-scopus-com.ezproxy.lib.swin.edu.au/authid/detail.uri?origin=resultslist&authorId=16148099900&zone=), [Anderson, C.L.](https://www-scopus-com.ezproxy.lib.swin.edu.au/authid/detail.uri?origin=resultslist&authorId=35556095500&zone=), [Vaca, F.E.](https://www-scopus-com.ezproxy.lib.swin.edu.au/authid/detail.uri?origin=resultslist&authorId=7003561651&zone=) 2008, [Traffic Injury Prevention](https://www-scopus-com.ezproxy.lib.swin.edu.au/sourceid/22112?origin=resultslist), 9(1), pp. 65-69

# [Relationship between traffic fatalities and drunk driving in Japan](https://www-scopus-com.ezproxy.lib.swin.edu.au/record/display.uri?eid=2-s2.0-33751179872&origin=resultslist&sort=plf-f&src=s&st1=%28predict*+OR+model%29+AND+%28%22serious+crash*%22+OR+%22serious+accident%22+OR+%22serious+collision%22+OR+%22fatal+accident%22+OR+%22fatal+crash%22+OR+%22fatal+collision%22+OR+%22road+deaths%22+OR+%22road+fatal*%22+OR+%22traffic+fatal*%22+OR+%22collision+fatal*%22+OR+%22accident+fatal*%22%29+AND+%28driver%29+&nlo=&nlr=&nls=&sid=34fc91d90b0a1eb0b79c181c6b17236b&sot=b&sdt=b&sl=290&s=TITLE-ABS-KEY%28%28predict*+OR+model%29+AND+%28%22serious+crash*%22+OR+%22serious+accident%22+OR+%22serious+collision%22+OR+%22fatal+accident%22+OR+%22fatal+crash%22+OR+%22fatal+collision%22+OR+%22road+deaths%22+OR+%22road+fatal*%22+OR+%22traffic+fatal*%22+OR+%22collision+fatal*%22+OR+%22accident+fatal*%22%29+AND+%28driver%29+%29+AND+PUBYEAR+%3e+1983&relpos=360&citeCnt=10&searchTerm=)

[Fujita, Y.](https://www-scopus-com.ezproxy.lib.swin.edu.au/authid/detail.uri?origin=resultslist&authorId=57206896070&zone=), [Shibata, A.](https://www-scopus-com.ezproxy.lib.swin.edu.au/authid/detail.uri?origin=resultslist&authorId=7201740751&zone=) 2006, [Traffic Injury Prevention](https://www-scopus-com.ezproxy.lib.swin.edu.au/sourceid/22112?origin=resultslist), 7(4), pp. 325-327

# [Factors associated with automobile accidents and survival](https://www-scopus-com.ezproxy.lib.swin.edu.au/record/display.uri?eid=2-s2.0-33745749589&origin=resultslist&sort=plf-f&src=s&st1=%28predict*+OR+model%29+AND+%28%22serious+crash*%22+OR+%22serious+accident%22+OR+%22serious+collision%22+OR+%22fatal+accident%22+OR+%22fatal+crash%22+OR+%22fatal+collision%22+OR+%22road+deaths%22+OR+%22road+fatal*%22+OR+%22traffic+fatal*%22+OR+%22collision+fatal*%22+OR+%22accident+fatal*%22%29+AND+%28driver%29+&nlo=&nlr=&nls=&sid=34fc91d90b0a1eb0b79c181c6b17236b&sot=b&sdt=b&sl=290&s=TITLE-ABS-KEY%28%28predict*+OR+model%29+AND+%28%22serious+crash*%22+OR+%22serious+accident%22+OR+%22serious+collision%22+OR+%22fatal+accident%22+OR+%22fatal+crash%22+OR+%22fatal+collision%22+OR+%22road+deaths%22+OR+%22road+fatal*%22+OR+%22traffic+fatal*%22+OR+%22collision+fatal*%22+OR+%22accident+fatal*%22%29+AND+%28driver%29+%29+AND+PUBYEAR+%3e+1983&relpos=366&citeCnt=14&searchTerm=)

[Kim, H.S.](https://www-scopus-com.ezproxy.lib.swin.edu.au/authid/detail.uri?origin=resultslist&authorId=7410128767&zone=), [Kim, H.J.](https://www-scopus-com.ezproxy.lib.swin.edu.au/authid/detail.uri?origin=resultslist&authorId=34770459800&zone=), [Son, B.](https://www-scopus-com.ezproxy.lib.swin.edu.au/authid/detail.uri?origin=resultslist&authorId=8985750900&zone=) 2006, [Accident Analysis and Prevention](https://www-scopus-com.ezproxy.lib.swin.edu.au/sourceid/19532?origin=resultslist), 38(5), pp. 981-987

# [Distractions and motor vehicle accidents: Data mining application on fatality analysis reporting system (FARS) data files](https://www-scopus-com.ezproxy.lib.swin.edu.au/record/display.uri?eid=2-s2.0-28644451085&origin=resultslist&sort=plf-f&src=s&st1=%28predict*+OR+model%29+AND+%28%22serious+crash*%22+OR+%22serious+accident%22+OR+%22serious+collision%22+OR+%22fatal+accident%22+OR+%22fatal+crash%22+OR+%22fatal+collision%22+OR+%22road+deaths%22+OR+%22road+fatal*%22+OR+%22traffic+fatal*%22+OR+%22collision+fatal*%22+OR+%22accident+fatal*%22%29+AND+%28driver%29+&nlo=&nlr=&nls=&sid=34fc91d90b0a1eb0b79c181c6b17236b&sot=b&sdt=b&sl=290&s=TITLE-ABS-KEY%28%28predict*+OR+model%29+AND+%28%22serious+crash*%22+OR+%22serious+accident%22+OR+%22serious+collision%22+OR+%22fatal+accident%22+OR+%22fatal+crash%22+OR+%22fatal+collision%22+OR+%22road+deaths%22+OR+%22road+fatal*%22+OR+%22traffic+fatal*%22+OR+%22collision+fatal*%22+OR+%22accident+fatal*%22%29+AND+%28driver%29+%29+AND+PUBYEAR+%3e+1983&relpos=373&citeCnt=18&searchTerm=)

[Tseng, W.-S.](https://www-scopus-com.ezproxy.lib.swin.edu.au/authid/detail.uri?origin=resultslist&authorId=9744026100&zone=), [Nguyen, H.](https://www-scopus-com.ezproxy.lib.swin.edu.au/authid/detail.uri?origin=resultslist&authorId=16024964700&zone=), [Liebowitz, J.](https://www-scopus-com.ezproxy.lib.swin.edu.au/authid/detail.uri?origin=resultslist&authorId=7005873491&zone=), [Agresti, W.](https://www-scopus-com.ezproxy.lib.swin.edu.au/authid/detail.uri?origin=resultslist&authorId=6603240299&zone=) 2005, [Industrial Management and Data Systems](https://www-scopus-com.ezproxy.lib.swin.edu.au/sourceid/19170?origin=resultslist), 105(9), pp. 1188-1205

# [Student drivers: A study of fatal motor vehicle crashes involving 16-year-old drivers](https://www-scopus-com.ezproxy.lib.swin.edu.au/record/display.uri?eid=2-s2.0-12444260664&origin=resultslist&sort=plf-f&src=s&st1=%28predict*+OR+model%29+AND+%28%22serious+crash*%22+OR+%22serious+accident%22+OR+%22serious+collision%22+OR+%22fatal+accident%22+OR+%22fatal+crash%22+OR+%22fatal+collision%22+OR+%22road+deaths%22+OR+%22road+fatal*%22+OR+%22traffic+fatal*%22+OR+%22collision+fatal*%22+OR+%22accident+fatal*%22%29+AND+%28driver%29+&nlo=&nlr=&nls=&sid=34fc91d90b0a1eb0b79c181c6b17236b&sot=b&sdt=b&sl=290&s=TITLE-ABS-KEY%28%28predict*+OR+model%29+AND+%28%22serious+crash*%22+OR+%22serious+accident%22+OR+%22serious+collision%22+OR+%22fatal+accident%22+OR+%22fatal+crash%22+OR+%22fatal+collision%22+OR+%22road+deaths%22+OR+%22road+fatal*%22+OR+%22traffic+fatal*%22+OR+%22collision+fatal*%22+OR+%22accident+fatal*%22%29+AND+%28driver%29+%29+AND+PUBYEAR+%3e+1983&relpos=386&citeCnt=52&searchTerm=)

[Gonzales, M.M.](https://www-scopus-com.ezproxy.lib.swin.edu.au/authid/detail.uri?origin=resultslist&authorId=16636406400&zone=), [Dickinson, L.M.](https://www-scopus-com.ezproxy.lib.swin.edu.au/authid/detail.uri?origin=resultslist&authorId=7006768802&zone=), [DiGuiseppi, C.](https://www-scopus-com.ezproxy.lib.swin.edu.au/authid/detail.uri?origin=resultslist&authorId=7003484763&zone=), [Lowenstein, S.R.](https://www-scopus-com.ezproxy.lib.swin.edu.au/authid/detail.uri?origin=resultslist&authorId=56950329500&zone=) 2005, [Annals of Emergency Medicine](https://www-scopus-com.ezproxy.lib.swin.edu.au/sourceid/15220?origin=resultslist), 45(2), pp. 140-146

# [Alcohol, public policy, and highway crashes: A time-series analysis of older-driver safety](https://www-scopus-com.ezproxy.lib.swin.edu.au/record/display.uri?eid=2-s2.0-13244283347&origin=resultslist&sort=plf-f&src=s&st1=%28predict*+OR+model%29+AND+%28%22serious+crash*%22+OR+%22serious+accident%22+OR+%22serious+collision%22+OR+%22fatal+accident%22+OR+%22fatal+crash%22+OR+%22fatal+collision%22+OR+%22road+deaths%22+OR+%22road+fatal*%22+OR+%22traffic+fatal*%22+OR+%22collision+fatal*%22+OR+%22accident+fatal*%22%29+AND+%28driver%29+&nlo=&nlr=&nls=&sid=34fc91d90b0a1eb0b79c181c6b17236b&sot=b&sdt=b&sl=290&s=TITLE-ABS-KEY%28%28predict*+OR+model%29+AND+%28%22serious+crash*%22+OR+%22serious+accident%22+OR+%22serious+collision%22+OR+%22fatal+accident%22+OR+%22fatal+crash%22+OR+%22fatal+collision%22+OR+%22road+deaths%22+OR+%22road+fatal*%22+OR+%22traffic+fatal*%22+OR+%22collision+fatal*%22+OR+%22accident+fatal*%22%29+AND+%28driver%29+%29+AND+PUBYEAR+%3e+1983&relpos=387&citeCnt=11&searchTerm=)

[McCarthy, P.S.](https://www-scopus-com.ezproxy.lib.swin.edu.au/authid/detail.uri?origin=resultslist&authorId=56214665800&zone=) 2005, [Journal of Transport Economics and Policy](https://www-scopus-com.ezproxy.lib.swin.edu.au/sourceid/18900?origin=resultslist), 39(1), pp. 109-125

# [The independent contribution of driver, crash, and vehicle characteristics to driver fatalities](https://www-scopus-com.ezproxy.lib.swin.edu.au/record/display.uri?eid=2-s2.0-0036826987&origin=resultslist&sort=plf-f&src=s&st1=%28predict*+OR+model%29+AND+%28%22serious+crash*%22+OR+%22serious+accident%22+OR+%22serious+collision%22+OR+%22fatal+accident%22+OR+%22fatal+crash%22+OR+%22fatal+collision%22+OR+%22road+deaths%22+OR+%22road+fatal*%22+OR+%22traffic+fatal*%22+OR+%22collision+fatal*%22+OR+%22accident+fatal*%22%29+AND+%28driver%29+&nlo=&nlr=&nls=&sid=34fc91d90b0a1eb0b79c181c6b17236b&sot=b&sdt=b&sl=290&s=TITLE-ABS-KEY%28%28predict*+OR+model%29+AND+%28%22serious+crash*%22+OR+%22serious+accident%22+OR+%22serious+collision%22+OR+%22fatal+accident%22+OR+%22fatal+crash%22+OR+%22fatal+collision%22+OR+%22road+deaths%22+OR+%22road+fatal*%22+OR+%22traffic+fatal*%22+OR+%22collision+fatal*%22+OR+%22accident+fatal*%22%29+AND+%28driver%29+%29+AND+PUBYEAR+%3e+1983&relpos=398&citeCnt=256&searchTerm=)

[Bédard, M.](https://www-scopus-com.ezproxy.lib.swin.edu.au/authid/detail.uri?origin=resultslist&authorId=35594547000&zone=), [Guyatt, G.H.](https://www-scopus-com.ezproxy.lib.swin.edu.au/authid/detail.uri?origin=resultslist&authorId=8841196600&zone=), [Stones, M.J.](https://www-scopus-com.ezproxy.lib.swin.edu.au/authid/detail.uri?origin=resultslist&authorId=7004267060&zone=), [Hirdes, J.P.](https://www-scopus-com.ezproxy.lib.swin.edu.au/authid/detail.uri?origin=resultslist&authorId=7005040465&zone=) 2002, [Accident Analysis and Prevention](https://www-scopus-com.ezproxy.lib.swin.edu.au/sourceid/19532?origin=resultslist), 34(6), pp. 717-727

# [Children in fatal crashes: Driver blood alcohol concentration and demographics of child passengers and their drivers](https://www-scopus-com.ezproxy.lib.swin.edu.au/record/display.uri?eid=2-s2.0-0036847521&origin=resultslist&sort=plf-f&src=s&st1=%28predict*+OR+model%29+AND+%28%22serious+crash*%22+OR+%22serious+accident%22+OR+%22serious+collision%22+OR+%22fatal+accident%22+OR+%22fatal+crash%22+OR+%22fatal+collision%22+OR+%22road+deaths%22+OR+%22road+fatal*%22+OR+%22traffic+fatal*%22+OR+%22collision+fatal*%22+OR+%22accident+fatal*%22%29+AND+%28driver%29+&nlo=&nlr=&nls=&sid=34fc91d90b0a1eb0b79c181c6b17236b&sot=b&sdt=b&sl=290&s=TITLE-ABS-KEY%28%28predict*+OR+model%29+AND+%28%22serious+crash*%22+OR+%22serious+accident%22+OR+%22serious+collision%22+OR+%22fatal+accident%22+OR+%22fatal+crash%22+OR+%22fatal+collision%22+OR+%22road+deaths%22+OR+%22road+fatal*%22+OR+%22traffic+fatal*%22+OR+%22collision+fatal*%22+OR+%22accident+fatal*%22%29+AND+%28driver%29+%29+AND+PUBYEAR+%3e+1983&relpos=399&citeCnt=19&searchTerm=)

[Voas, R.B.](https://www-scopus-com.ezproxy.lib.swin.edu.au/authid/detail.uri?origin=resultslist&authorId=7006351337&zone=), [Fisher, D.A.](https://www-scopus-com.ezproxy.lib.swin.edu.au/authid/detail.uri?origin=resultslist&authorId=57001040500&zone=), [Tippetts, A.S.](https://www-scopus-com.ezproxy.lib.swin.edu.au/authid/detail.uri?origin=resultslist&authorId=6701828070&zone=) 2002. [Addiction](https://www-scopus-com.ezproxy.lib.swin.edu.au/sourceid/24745?origin=resultslist), 97(11), pp. 1439-1448

# [Sex differences in single vehicle fatal crashes: A research note](https://www-scopus-com.ezproxy.lib.swin.edu.au/record/display.uri?eid=2-s2.0-0036107277&origin=resultslist&sort=plf-f&src=s&st1=%28predict*+OR+model%29+AND+%28%22serious+crash*%22+OR+%22serious+accident%22+OR+%22serious+collision%22+OR+%22fatal+accident%22+OR+%22fatal+crash%22+OR+%22fatal+collision%22+OR+%22road+deaths%22+OR+%22road+fatal*%22+OR+%22traffic+fatal*%22+OR+%22collision+fatal*%22+OR+%22accident+fatal*%22%29+AND+%28driver%29+&nlo=&nlr=&nls=&sid=34fc91d90b0a1eb0b79c181c6b17236b&sot=b&sdt=b&sl=290&s=TITLE-ABS-KEY%28%28predict*+OR+model%29+AND+%28%22serious+crash*%22+OR+%22serious+accident%22+OR+%22serious+collision%22+OR+%22fatal+accident%22+OR+%22fatal+crash%22+OR+%22fatal+collision%22+OR+%22road+deaths%22+OR+%22road+fatal*%22+OR+%22traffic+fatal*%22+OR+%22collision+fatal*%22+OR+%22accident+fatal*%22%29+AND+%28driver%29+%29+AND+PUBYEAR+%3e+1983&relpos=400&citeCnt=14&searchTerm=)

[Bergdahl, J.](https://www-scopus-com.ezproxy.lib.swin.edu.au/authid/detail.uri?origin=resultslist&authorId=9939503000&zone=), [Norris, M.R.](https://www-scopus-com.ezproxy.lib.swin.edu.au/authid/detail.uri?origin=resultslist&authorId=15825543300&zone=) 2002, [Social Science Journal](https://www-scopus-com.ezproxy.lib.swin.edu.au/sourceid/26437?origin=resultslist), 39(2), pp. 287-293

# [Fatality risk assessment and modeling of drivers responsibility for causing traffic accidents in Dubai.](https://www-scopus-com.ezproxy.lib.swin.edu.au/record/display.uri?eid=2-s2.0-0036882148&origin=resultslist&sort=plf-f&src=s&st1=%28predict*+OR+model%29+AND+%28%22serious+crash*%22+OR+%22serious+accident%22+OR+%22serious+collision%22+OR+%22fatal+accident%22+OR+%22fatal+crash%22+OR+%22fatal+collision%22+OR+%22road+deaths%22+OR+%22road+fatal*%22+OR+%22traffic+fatal*%22+OR+%22collision+fatal*%22+OR+%22accident+fatal*%22%29+AND+%28driver%29+&nlo=&nlr=&nls=&sid=34fc91d90b0a1eb0b79c181c6b17236b&sot=b&sdt=b&sl=290&s=TITLE-ABS-KEY%28%28predict*+OR+model%29+AND+%28%22serious+crash*%22+OR+%22serious+accident%22+OR+%22serious+collision%22+OR+%22fatal+accident%22+OR+%22fatal+crash%22+OR+%22fatal+collision%22+OR+%22road+deaths%22+OR+%22road+fatal*%22+OR+%22traffic+fatal*%22+OR+%22collision+fatal*%22+OR+%22accident+fatal*%22%29+AND+%28driver%29+%29+AND+PUBYEAR+%3e+1983&relpos=403&citeCnt=15&searchTerm=)

[Abdalla, I.M.](https://www-scopus-com.ezproxy.lib.swin.edu.au/authid/detail.uri?origin=resultslist&authorId=7004151272&zone=) 2002, [Journal of safety research](https://www-scopus-com.ezproxy.lib.swin.edu.au/sourceid/29284?origin=resultslist), 33(4), pp. 483-496

# [Accident risks of car drivers in wintertime traffic](https://www-scopus-com.ezproxy.lib.swin.edu.au/record/display.uri?eid=2-s2.0-26944497665&origin=resultslist&sort=plf-f&src=s&st1=%28predict*+OR+model%29+AND+%28%22serious+crash*%22+OR+%22serious+accident%22+OR+%22serious+collision%22+OR+%22fatal+accident%22+OR+%22fatal+crash%22+OR+%22fatal+collision%22+OR+%22road+deaths%22+OR+%22road+fatal*%22+OR+%22traffic+fatal*%22+OR+%22collision+fatal*%22+OR+%22accident+fatal*%22%29+AND+%28driver%29+&nlo=&nlr=&nls=&sid=34fc91d90b0a1eb0b79c181c6b17236b&sot=b&sdt=b&sl=290&s=TITLE-ABS-KEY%28%28predict*+OR+model%29+AND+%28%22serious+crash*%22+OR+%22serious+accident%22+OR+%22serious+collision%22+OR+%22fatal+accident%22+OR+%22fatal+crash%22+OR+%22fatal+collision%22+OR+%22road+deaths%22+OR+%22road+fatal*%22+OR+%22traffic+fatal*%22+OR+%22collision+fatal*%22+OR+%22accident+fatal*%22%29+AND+%28driver%29+%29+AND+PUBYEAR+%3e+1983&relpos=417&citeCnt=1&searchTerm=)

[Roine, M.](https://www-scopus-com.ezproxy.lib.swin.edu.au/authid/detail.uri?origin=resultslist&authorId=6506484634&zone=) 1999, [VTT Publications](https://www-scopus-com.ezproxy.lib.swin.edu.au/sourceid/52733?origin=resultslist), (401), pp. 2-137

# [Crash involvement rates by driver gender and the role of average annual mileage](https://www-scopus-com.ezproxy.lib.swin.edu.au/record/display.uri?eid=2-s2.0-0031227729&origin=resultslist&sort=plf-f&src=s&st1=%28predict*+OR+model%29+AND+%28%22serious+crash*%22+OR+%22serious+accident%22+OR+%22serious+collision%22+OR+%22fatal+accident%22+OR+%22fatal+crash%22+OR+%22fatal+collision%22+OR+%22road+deaths%22+OR+%22road+fatal*%22+OR+%22traffic+fatal*%22+OR+%22collision+fatal*%22+OR+%22accident+fatal*%22%29+AND+%28driver%29+&nlo=&nlr=&nls=&sid=34fc91d90b0a1eb0b79c181c6b17236b&sot=b&sdt=b&sl=290&s=TITLE-ABS-KEY%28%28predict*+OR+model%29+AND+%28%22serious+crash*%22+OR+%22serious+accident%22+OR+%22serious+collision%22+OR+%22fatal+accident%22+OR+%22fatal+crash%22+OR+%22fatal+collision%22+OR+%22road+deaths%22+OR+%22road+fatal*%22+OR+%22traffic+fatal*%22+OR+%22collision+fatal*%22+OR+%22accident+fatal*%22%29+AND+%28driver%29+%29+AND+PUBYEAR+%3e+1983&relpos=431&citeCnt=83&searchTerm=)

[Massie, D.L.](https://www-scopus-com.ezproxy.lib.swin.edu.au/authid/detail.uri?origin=resultslist&authorId=7003723781&zone=), [Green, P.E.](https://www-scopus-com.ezproxy.lib.swin.edu.au/authid/detail.uri?origin=resultslist&authorId=36999771600&zone=), [Campbell, K.L.](https://www-scopus-com.ezproxy.lib.swin.edu.au/authid/detail.uri?origin=resultslist&authorId=7402003660&zone=) 1997, [Accident Analysis and Prevention](https://www-scopus-com.ezproxy.lib.swin.edu.au/sourceid/19532?origin=resultslist), 29(5), pp. 675-685

# [The driver's role in fatal two-car crashes: A paired "case-control" study](https://www-scopus-com.ezproxy.lib.swin.edu.au/record/display.uri?eid=2-s2.0-0026320728&origin=resultslist&sort=plf-f&src=s&st1=%28predict*+OR+model%29+AND+%28%22serious+crash*%22+OR+%22serious+accident%22+OR+%22serious+collision%22+OR+%22fatal+accident%22+OR+%22fatal+crash%22+OR+%22fatal+collision%22+OR+%22road+deaths%22+OR+%22road+fatal*%22+OR+%22traffic+fatal*%22+OR+%22collision+fatal*%22+OR+%22accident+fatal*%22%29+AND+%28driver%29+&nlo=&nlr=&nls=&sid=34fc91d90b0a1eb0b79c181c6b17236b&sot=b&sdt=b&sl=290&s=TITLE-ABS-KEY%28%28predict*+OR+model%29+AND+%28%22serious+crash*%22+OR+%22serious+accident%22+OR+%22serious+collision%22+OR+%22fatal+accident%22+OR+%22fatal+crash%22+OR+%22fatal+collision%22+OR+%22road+deaths%22+OR+%22road+fatal*%22+OR+%22traffic+fatal*%22+OR+%22collision+fatal*%22+OR+%22accident+fatal*%22%29+AND+%28driver%29+%29+AND+PUBYEAR+%3e+1983&relpos=448&citeCnt=57&searchTerm=)

[Perneger, T.](https://www-scopus-com.ezproxy.lib.swin.edu.au/authid/detail.uri?origin=resultslist&authorId=7102259897&zone=), [Smith, G.S.](https://www-scopus-com.ezproxy.lib.swin.edu.au/authid/detail.uri?origin=resultslist&authorId=7406738484&zone=) 1991, [American Journal of Epidemiology](https://www-scopus-com.ezproxy.lib.swin.edu.au/sourceid/27058?origin=resultslist), 134(10), pp. 1138-1145

# [An application of proportional hazards model to study the recurrent time between traffic accidents or infractions and subsequent fatal automobile crashes, 1986-1988](https://www-scopus-com.ezproxy.lib.swin.edu.au/record/display.uri?eid=2-s2.0-28844463122&origin=resultslist&sort=plf-f&src=s&st1=%28predict*+OR+model%29+AND+%28%22serious+crash*%22+OR+%22serious+accident%22+OR+%22serious+collision%22+OR+%22fatal+accident%22+OR+%22fatal+crash%22+OR+%22fatal+collision%22+OR+%22road+deaths%22+OR+%22road+fatal*%22+OR+%22traffic+fatal*%22+OR+%22collision+fatal*%22+OR+%22accident+fatal*%22%29+AND+%28driver%29+&nlo=&nlr=&nls=&sid=34fc91d90b0a1eb0b79c181c6b17236b&sot=b&sdt=b&sl=290&s=TITLE-ABS-KEY%28%28predict*+OR+model%29+AND+%28%22serious+crash*%22+OR+%22serious+accident%22+OR+%22serious+collision%22+OR+%22fatal+accident%22+OR+%22fatal+crash%22+OR+%22fatal+collision%22+OR+%22road+deaths%22+OR+%22road+fatal*%22+OR+%22traffic+fatal*%22+OR+%22collision+fatal*%22+OR+%22accident+fatal*%22%29+AND+%28driver%29+%29+AND+PUBYEAR+%3e+1983&relpos=450&citeCnt=0&searchTerm=)

[Lui, K.-J.](https://www-scopus-com.ezproxy.lib.swin.edu.au/authid/detail.uri?origin=resultslist&authorId=7103389973&zone=), [Pollock, D.](https://www-scopus-com.ezproxy.lib.swin.edu.au/authid/detail.uri?origin=resultslist&authorId=7202569042&zone=) 1991, [Journal of Safety Research](https://www-scopus-com.ezproxy.lib.swin.edu.au/sourceid/29284?origin=resultslist), 22(3), pp. 163-170

# [Administrative license suspension: Does length of suspension matter?](http://apps.webofknowledge.com.ezproxy.lib.swin.edu.au/full_record.do?product=WOS&search_mode=GeneralSearch&qid=1&SID=C5jzgzYMvSSmhbkgBbF&page=2&doc=59)

By: [Fell, James C.](http://apps.webofknowledge.com.ezproxy.lib.swin.edu.au/DaisyOneClickSearch.do?product=WOS&search_mode=DaisyOneClickSearch&colName=WOS&SID=C5jzgzYMvSSmhbkgBbF&author_name=Fell,%20James%20C.&dais_id=27878808&excludeEventConfig=ExcludeIfFromFullRecPage); [Scherer, Michael](http://apps.webofknowledge.com.ezproxy.lib.swin.edu.au/DaisyOneClickSearch.do?product=WOS&search_mode=DaisyOneClickSearch&colName=WOS&SID=C5jzgzYMvSSmhbkgBbF&author_name=Scherer,%20Michael&dais_id=951581&excludeEventConfig=ExcludeIfFromFullRecPage)

[TRAFFIC INJURY PREVENTION](javascript:;)  Volume: 18   Issue: 6   Pages: 577-584   Published: 2017

# [ANALYSIS OF FACTORS INFLUENCING THE VEHICLE DAMAGE LEVEL IN FATAL TRUCK-RELATED ACCIDENTS AND DIFFERENCES IN RURAL AND URBAN AREAS](http://apps.webofknowledge.com.ezproxy.lib.swin.edu.au/full_record.do?product=WOS&search_mode=GeneralSearch&qid=1&SID=C5jzgzYMvSSmhbkgBbF&page=2&doc=92)

By: [Li Linchao](http://apps.webofknowledge.com.ezproxy.lib.swin.edu.au/DaisyOneClickSearch.do?product=WOS&search_mode=DaisyOneClickSearch&colName=WOS&SID=C5jzgzYMvSSmhbkgBbF&author_name=Li%20Linchao&dais_id=3125625&excludeEventConfig=ExcludeIfFromFullRecPage); [Fratrovic, Tomislav](http://apps.webofknowledge.com.ezproxy.lib.swin.edu.au/DaisyOneClickSearch.do?product=WOS&search_mode=DaisyOneClickSearch&colName=WOS&SID=C5jzgzYMvSSmhbkgBbF&author_name=Fratrovic,%20Tomislav&dais_id=5743554&excludeEventConfig=ExcludeIfFromFullRecPage)

[PROMET-TRAFFIC & TRANSPORTATION](javascript:;)  Volume: 28   Issue: 4   Pages: 331-340   Published: 2016

# [A comprehensive examination of US laws enacted to reduce alcohol-related crashes among underage drivers](http://apps.webofknowledge.com.ezproxy.lib.swin.edu.au/full_record.do?product=WOS&search_mode=GeneralSearch&qid=1&SID=C5jzgzYMvSSmhbkgBbF&page=2&doc=95)

By: [Romano, Eduardo](http://apps.webofknowledge.com.ezproxy.lib.swin.edu.au/DaisyOneClickSearch.do?product=WOS&search_mode=DaisyOneClickSearch&colName=WOS&SID=C5jzgzYMvSSmhbkgBbF&author_name=Romano,%20Eduardo&dais_id=178153&excludeEventConfig=ExcludeIfFromFullRecPage); [Scherer, Michael](http://apps.webofknowledge.com.ezproxy.lib.swin.edu.au/DaisyOneClickSearch.do?product=WOS&search_mode=DaisyOneClickSearch&colName=WOS&SID=C5jzgzYMvSSmhbkgBbF&author_name=Scherer,%20Michael&dais_id=951581&excludeEventConfig=ExcludeIfFromFullRecPage); [Fell, James](http://apps.webofknowledge.com.ezproxy.lib.swin.edu.au/DaisyOneClickSearch.do?product=WOS&search_mode=DaisyOneClickSearch&colName=WOS&SID=C5jzgzYMvSSmhbkgBbF&author_name=Fell,%20James&dais_id=27878808&excludeEventConfig=ExcludeIfFromFullRecPage); et al.

[JOURNAL OF SAFETY RESEARCH](javascript:;)  Volume: 55   Pages: 213-221   Published: DEC 2015

# [Pooling data from fatality analysis reporting system (FARS) and generalized estimates system (GES) to explore the continuum of injury severity spectrum](http://apps.webofknowledge.com.ezproxy.lib.swin.edu.au/full_record.do?product=WOS&search_mode=GeneralSearch&qid=1&SID=C5jzgzYMvSSmhbkgBbF&page=2&doc=97)

By: [Yasmin, Sharnsunnahar](http://apps.webofknowledge.com.ezproxy.lib.swin.edu.au/DaisyOneClickSearch.do?product=WOS&search_mode=DaisyOneClickSearch&colName=WOS&SID=C5jzgzYMvSSmhbkgBbF&author_name=Yasmin,%20Sharnsunnahar&dais_id=1727550&excludeEventConfig=ExcludeIfFromFullRecPage); [Eluru, Naveen](http://apps.webofknowledge.com.ezproxy.lib.swin.edu.au/DaisyOneClickSearch.do?product=WOS&search_mode=DaisyOneClickSearch&colName=WOS&SID=C5jzgzYMvSSmhbkgBbF&author_name=Eluru,%20Naveen&dais_id=461953&excludeEventConfig=ExcludeIfFromFullRecPage); [Pinjari, Abdul R.](http://apps.webofknowledge.com.ezproxy.lib.swin.edu.au/DaisyOneClickSearch.do?product=WOS&search_mode=DaisyOneClickSearch&colName=WOS&SID=C5jzgzYMvSSmhbkgBbF&author_name=Pinjari,%20Abdul%20R.&dais_id=1018389&excludeEventConfig=ExcludeIfFromFullRecPage)

[ACCIDENT ANALYSIS AND PREVENTION](javascript:;)  Volume: 84   Pages: 112-127   Published: NOV 2015

# [Analyzing the continuum of fatal crashes: A generalized ordered approach](http://apps.webofknowledge.com.ezproxy.lib.swin.edu.au/full_record.do?product=WOS&search_mode=GeneralSearch&qid=1&SID=C5jzgzYMvSSmhbkgBbF&page=3&doc=105)

By: [Yasmin, Shamsunnahar](http://apps.webofknowledge.com.ezproxy.lib.swin.edu.au/DaisyOneClickSearch.do?product=WOS&search_mode=DaisyOneClickSearch&colName=WOS&SID=C5jzgzYMvSSmhbkgBbF&author_name=Yasmin,%20Shamsunnahar&dais_id=1727550&excludeEventConfig=ExcludeIfFromFullRecPage); [Eluru, Naveen](http://apps.webofknowledge.com.ezproxy.lib.swin.edu.au/DaisyOneClickSearch.do?product=WOS&search_mode=DaisyOneClickSearch&colName=WOS&SID=C5jzgzYMvSSmhbkgBbF&author_name=Eluru,%20Naveen&dais_id=461953&excludeEventConfig=ExcludeIfFromFullRecPage); [Pinjari, Abdul R.](http://apps.webofknowledge.com.ezproxy.lib.swin.edu.au/DaisyOneClickSearch.do?product=WOS&search_mode=DaisyOneClickSearch&colName=WOS&SID=C5jzgzYMvSSmhbkgBbF&author_name=Pinjari,%20Abdul%20R.&dais_id=1018389&excludeEventConfig=ExcludeIfFromFullRecPage)

ANALYTIC METHODS IN ACCIDENT RESEARCH   Volume: 7   Pages: 1-15   Published: JUL 2015

# [Can cars and trucks coexist peacefully on highways? Analyzing the effectiveness of road safety policies in Europe](http://apps.webofknowledge.com.ezproxy.lib.swin.edu.au/full_record.do?product=WOS&search_mode=GeneralSearch&qid=1&SID=C5jzgzYMvSSmhbkgBbF&page=3&doc=108)

By: [Castillo-Manzano, Jose I.](http://apps.webofknowledge.com.ezproxy.lib.swin.edu.au/DaisyOneClickSearch.do?product=WOS&search_mode=DaisyOneClickSearch&colName=WOS&SID=C5jzgzYMvSSmhbkgBbF&author_name=Castillo-Manzano,%20Jose%20I.&dais_id=726466&excludeEventConfig=ExcludeIfFromFullRecPage); [Castro-Nano, Mercedes](http://apps.webofknowledge.com.ezproxy.lib.swin.edu.au/DaisyOneClickSearch.do?product=WOS&search_mode=DaisyOneClickSearch&colName=WOS&SID=C5jzgzYMvSSmhbkgBbF&author_name=Castro-Nano,%20Mercedes&dais_id=18256075&excludeEventConfig=ExcludeIfFromFullRecPage); [Fageda, Xavier](http://apps.webofknowledge.com.ezproxy.lib.swin.edu.au/DaisyOneClickSearch.do?product=WOS&search_mode=DaisyOneClickSearch&colName=WOS&SID=C5jzgzYMvSSmhbkgBbF&author_name=Fageda,%20Xavier&dais_id=608153&excludeEventConfig=ExcludeIfFromFullRecPage)

[ACCIDENT ANALYSIS AND PREVENTION](javascript:;)  Volume: 77   Pages: 120-126   Published: APR 2015

# [Age, period, and cohort effects in motor vehicle mortality in the United States, 1980-2010: The role of sex, alcohol involvement, and position in vehicle](http://apps.webofknowledge.com.ezproxy.lib.swin.edu.au/full_record.do?product=WOS&search_mode=GeneralSearch&qid=1&SID=C5jzgzYMvSSmhbkgBbF&page=3&doc=111)

By: [Macinko, James](http://apps.webofknowledge.com.ezproxy.lib.swin.edu.au/DaisyOneClickSearch.do?product=WOS&search_mode=DaisyOneClickSearch&colName=WOS&SID=C5jzgzYMvSSmhbkgBbF&author_name=Macinko,%20James&dais_id=346592&excludeEventConfig=ExcludeIfFromFullRecPage); [Silver, Diana](http://apps.webofknowledge.com.ezproxy.lib.swin.edu.au/DaisyOneClickSearch.do?product=WOS&search_mode=DaisyOneClickSearch&colName=WOS&SID=C5jzgzYMvSSmhbkgBbF&author_name=Silver,%20Diana&dais_id=1387231&excludeEventConfig=ExcludeIfFromFullRecPage); [Bae, Jin Yung](http://apps.webofknowledge.com.ezproxy.lib.swin.edu.au/DaisyOneClickSearch.do?product=WOS&search_mode=DaisyOneClickSearch&colName=WOS&SID=C5jzgzYMvSSmhbkgBbF&author_name=Bae,%20Jin%20Yung&dais_id=3451225&excludeEventConfig=ExcludeIfFromFullRecPage)

[JOURNAL OF SAFETY RESEARCH](javascript:;)  Volume: 52   Pages: 47-57   Published: FEB 2015

# [Are traffic violators criminals? Searching for answers in the experiences of European countries](http://apps.webofknowledge.com.ezproxy.lib.swin.edu.au/full_record.do?product=WOS&search_mode=GeneralSearch&qid=1&SID=C5jzgzYMvSSmhbkgBbF&page=3&doc=112)

By: [Castillo-Manzano, Jose I.](http://apps.webofknowledge.com.ezproxy.lib.swin.edu.au/DaisyOneClickSearch.do?product=WOS&search_mode=DaisyOneClickSearch&colName=WOS&SID=C5jzgzYMvSSmhbkgBbF&author_name=Castillo-Manzano,%20Jose%20I.&dais_id=726466&excludeEventConfig=ExcludeIfFromFullRecPage); [Castro-Nuno, Mercedes](http://apps.webofknowledge.com.ezproxy.lib.swin.edu.au/DaisyOneClickSearch.do?product=WOS&search_mode=DaisyOneClickSearch&colName=WOS&SID=C5jzgzYMvSSmhbkgBbF&author_name=Castro-Nuno,%20Mercedes&dais_id=1801528&excludeEventConfig=ExcludeIfFromFullRecPage); [Fageda, Xavier](http://apps.webofknowledge.com.ezproxy.lib.swin.edu.au/DaisyOneClickSearch.do?product=WOS&search_mode=DaisyOneClickSearch&colName=WOS&SID=C5jzgzYMvSSmhbkgBbF&author_name=Fageda,%20Xavier&dais_id=608153&excludeEventConfig=ExcludeIfFromFullRecPage)

[TRANSPORT POLICY](javascript:;)  Volume: 38   Pages: 86-94   Published: FEB 2015

# [Variation in US traffic safety policy environments and motor vehicle fatalities 1980-2010](http://apps.webofknowledge.com.ezproxy.lib.swin.edu.au/full_record.do?product=WOS&search_mode=GeneralSearch&qid=1&SID=C5jzgzYMvSSmhbkgBbF&page=3&doc=134)

By: [Silver, D.](http://apps.webofknowledge.com.ezproxy.lib.swin.edu.au/DaisyOneClickSearch.do?product=WOS&search_mode=DaisyOneClickSearch&colName=WOS&SID=C5jzgzYMvSSmhbkgBbF&author_name=Silver,%20D.&dais_id=1387231&excludeEventConfig=ExcludeIfFromFullRecPage); [Macinko, J.](http://apps.webofknowledge.com.ezproxy.lib.swin.edu.au/DaisyOneClickSearch.do?product=WOS&search_mode=DaisyOneClickSearch&colName=WOS&SID=C5jzgzYMvSSmhbkgBbF&author_name=Macinko,%20J.&dais_id=346592&excludeEventConfig=ExcludeIfFromFullRecPage); [Bae, J. Y.](http://apps.webofknowledge.com.ezproxy.lib.swin.edu.au/DaisyOneClickSearch.do?product=WOS&search_mode=DaisyOneClickSearch&colName=WOS&SID=C5jzgzYMvSSmhbkgBbF&author_name=Bae,%20J.%20Y.&dais_id=3451225&excludeEventConfig=ExcludeIfFromFullRecPage); et al.

[PUBLIC HEALTH](javascript:;)  Volume: 127   Issue: 12   Pages: 1117-1125   Published: DEC 2013

# [The deterrent effects of the penalty points system for driving offences: a regression discontinuity approach](http://apps.webofknowledge.com.ezproxy.lib.swin.edu.au/full_record.do?product=WOS&search_mode=GeneralSearch&qid=1&SID=C5jzgzYMvSSmhbkgBbF&page=3&doc=140)

By: [De Paola, Maria](http://apps.webofknowledge.com.ezproxy.lib.swin.edu.au/DaisyOneClickSearch.do?product=WOS&search_mode=DaisyOneClickSearch&colName=WOS&SID=C5jzgzYMvSSmhbkgBbF&author_name=De%20Paola,%20Maria&dais_id=763405&excludeEventConfig=ExcludeIfFromFullRecPage); [Scoppa, Vincenzo](http://apps.webofknowledge.com.ezproxy.lib.swin.edu.au/DaisyOneClickSearch.do?product=WOS&search_mode=DaisyOneClickSearch&colName=WOS&SID=C5jzgzYMvSSmhbkgBbF&author_name=Scoppa,%20Vincenzo&dais_id=1036959&excludeEventConfig=ExcludeIfFromFullRecPage); [Falcone, Mariatiziana](http://apps.webofknowledge.com.ezproxy.lib.swin.edu.au/DaisyOneClickSearch.do?product=WOS&search_mode=DaisyOneClickSearch&colName=WOS&SID=C5jzgzYMvSSmhbkgBbF&author_name=Falcone,%20Mariatiziana&dais_id=25210827&excludeEventConfig=ExcludeIfFromFullRecPage)

[EMPIRICAL ECONOMICS](javascript:;)  Volume: 45   Issue: 2   Pages: 965-985   Published: OCT 2013

# [Fatal accident distribution by age, gender and head injury, and death probability at accident scene in Mashhad, Iran, 2006-2009](http://apps.webofknowledge.com.ezproxy.lib.swin.edu.au/full_record.do?product=WOS&search_mode=GeneralSearch&qid=1&SID=C5jzgzYMvSSmhbkgBbF&page=3&doc=148)

By: [Dovom, Hossein Zangooei](http://apps.webofknowledge.com.ezproxy.lib.swin.edu.au/DaisyOneClickSearch.do?product=WOS&search_mode=DaisyOneClickSearch&colName=WOS&SID=C5jzgzYMvSSmhbkgBbF&author_name=Dovom,%20Hossein%20Zangooei&dais_id=12569337&excludeEventConfig=ExcludeIfFromFullRecPage); [Shafahi, Yousef](http://apps.webofknowledge.com.ezproxy.lib.swin.edu.au/DaisyOneClickSearch.do?product=WOS&search_mode=DaisyOneClickSearch&colName=WOS&SID=C5jzgzYMvSSmhbkgBbF&author_name=Shafahi,%20Yousef&dais_id=1403110&excludeEventConfig=ExcludeIfFromFullRecPage); [Dovom, Mehdi Zangooei](http://apps.webofknowledge.com.ezproxy.lib.swin.edu.au/DaisyOneClickSearch.do?product=WOS&search_mode=DaisyOneClickSearch&colName=WOS&SID=C5jzgzYMvSSmhbkgBbF&author_name=Dovom,%20Mehdi%20Zangooei&dais_id=11009458&excludeEventConfig=ExcludeIfFromFullRecPage)

[INTERNATIONAL JOURNAL OF INJURY CONTROL AND SAFETY PROMOTION](javascript:;)  Volume: 20   Issue: 2  Pages: 121-133   Published: JUN 1 2013

# [A two-stage mining framework to explore key risk conditions on one-vehicle crash severity](http://apps.webofknowledge.com.ezproxy.lib.swin.edu.au/full_record.do?product=WOS&search_mode=GeneralSearch&qid=1&SID=C5jzgzYMvSSmhbkgBbF&page=4&doc=155)

By: [Chiou, Yu-Chiun](http://apps.webofknowledge.com.ezproxy.lib.swin.edu.au/DaisyOneClickSearch.do?product=WOS&search_mode=DaisyOneClickSearch&colName=WOS&SID=C5jzgzYMvSSmhbkgBbF&author_name=Chiou,%20Yu-Chiun&dais_id=407794&excludeEventConfig=ExcludeIfFromFullRecPage); [Lan, Lawrence W.](http://apps.webofknowledge.com.ezproxy.lib.swin.edu.au/DaisyOneClickSearch.do?product=WOS&search_mode=DaisyOneClickSearch&colName=WOS&SID=C5jzgzYMvSSmhbkgBbF&author_name=Lan,%20Lawrence%20W.&dais_id=667770&excludeEventConfig=ExcludeIfFromFullRecPage); [Chen, Wen-Pin](http://apps.webofknowledge.com.ezproxy.lib.swin.edu.au/DaisyOneClickSearch.do?product=WOS&search_mode=DaisyOneClickSearch&colName=WOS&SID=C5jzgzYMvSSmhbkgBbF&author_name=Chen,%20Wen-Pin&dais_id=938108&excludeEventConfig=ExcludeIfFromFullRecPage)

[ACCIDENT ANALYSIS AND PREVENTION](javascript:;)  Volume: 50   Pages: 405-415   Published: JAN 2013

# [Graduated Driver Licensing and Fatal Crashes Involving 16-to 19-Year-Old Drivers](http://apps.webofknowledge.com.ezproxy.lib.swin.edu.au/full_record.do?product=WOS&search_mode=GeneralSearch&qid=1&SID=C5jzgzYMvSSmhbkgBbF&page=4&doc=173)

By: [Masten, Scott V.](http://apps.webofknowledge.com.ezproxy.lib.swin.edu.au/DaisyOneClickSearch.do?product=WOS&search_mode=DaisyOneClickSearch&colName=WOS&SID=C5jzgzYMvSSmhbkgBbF&author_name=Masten,%20Scott%20V.&dais_id=3256038&excludeEventConfig=ExcludeIfFromFullRecPage); [Foss, Robert D.](http://apps.webofknowledge.com.ezproxy.lib.swin.edu.au/DaisyOneClickSearch.do?product=WOS&search_mode=DaisyOneClickSearch&colName=WOS&SID=C5jzgzYMvSSmhbkgBbF&author_name=Foss,%20Robert%20D.&dais_id=483390&excludeEventConfig=ExcludeIfFromFullRecPage); [Marshall, Stephen W.](http://apps.webofknowledge.com.ezproxy.lib.swin.edu.au/DaisyOneClickSearch.do?product=WOS&search_mode=DaisyOneClickSearch&colName=WOS&SID=C5jzgzYMvSSmhbkgBbF&author_name=Marshall,%20Stephen%20W.&dais_id=48687&excludeEventConfig=ExcludeIfFromFullRecPage)

[JAMA-JOURNAL OF THE AMERICAN MEDICAL ASSOCIATION](javascript:;)  Volume: 306   Issue: 10   Pages: 1098-1103   Published: SEP 14 2011

# [Gasoline prices and their relationship to drunk-driving crashes](http://apps.webofknowledge.com.ezproxy.lib.swin.edu.au/full_record.do?product=WOS&search_mode=GeneralSearch&qid=1&SID=C5jzgzYMvSSmhbkgBbF&page=4&doc=179)

By: [Chi, Guangqing](http://apps.webofknowledge.com.ezproxy.lib.swin.edu.au/DaisyOneClickSearch.do?product=WOS&search_mode=DaisyOneClickSearch&colName=WOS&SID=C5jzgzYMvSSmhbkgBbF&author_name=Chi,%20Guangqing&dais_id=1103570&excludeEventConfig=ExcludeIfFromFullRecPage); [Zhou, Xuan](http://apps.webofknowledge.com.ezproxy.lib.swin.edu.au/DaisyOneClickSearch.do?product=WOS&search_mode=DaisyOneClickSearch&colName=WOS&SID=C5jzgzYMvSSmhbkgBbF&author_name=Zhou,%20Xuan&dais_id=1557204&excludeEventConfig=ExcludeIfFromFullRecPage); [McClure, Timothy E.](http://apps.webofknowledge.com.ezproxy.lib.swin.edu.au/DaisyOneClickSearch.do?product=WOS&search_mode=DaisyOneClickSearch&colName=WOS&SID=C5jzgzYMvSSmhbkgBbF&author_name=McClure,%20Timothy%20E.&dais_id=3802550&excludeEventConfig=ExcludeIfFromFullRecPage); et al.

[ACCIDENT ANALYSIS AND PREVENTION](javascript:;)  Volume: 43   Issue: 1   Pages: 194-203   Published: JAN 2011

# [Measurement and dimension of road fatality in Brunei](http://apps.webofknowledge.com.ezproxy.lib.swin.edu.au/full_record.do?product=WOS&search_mode=GeneralSearch&qid=1&SID=C5jzgzYMvSSmhbkgBbF&page=4&doc=181)

[INTERNATIONAL JOURNAL OF INJURY CONTROL AND SAFETY PROMOTION](javascript:;)  Volume: 18   Issue: 1  Pages: 45-55   Article Number: PII 934941771   Published: 2011

# [ALCOHOL PREVALENCE, ALCOHOL POLICIES, AND CHILD FATAL INJURY RATES FROM MOTOR VEHICLE CRASHES](http://apps.webofknowledge.com.ezproxy.lib.swin.edu.au/full_record.do?product=WOS&search_mode=GeneralSearch&qid=1&SID=C5jzgzYMvSSmhbkgBbF&page=4&doc=186)

By: [Sen, Bisakha](http://apps.webofknowledge.com.ezproxy.lib.swin.edu.au/DaisyOneClickSearch.do?product=WOS&search_mode=DaisyOneClickSearch&colName=WOS&SID=C5jzgzYMvSSmhbkgBbF&author_name=Sen,%20Bisakha&dais_id=242691&excludeEventConfig=ExcludeIfFromFullRecPage); [Campbell, Christine M.](http://apps.webofknowledge.com.ezproxy.lib.swin.edu.au/DaisyOneClickSearch.do?product=WOS&search_mode=DaisyOneClickSearch&colName=WOS&SID=C5jzgzYMvSSmhbkgBbF&author_name=Campbell,%20Christine%20M.&dais_id=848765&excludeEventConfig=ExcludeIfFromFullRecPage)

[CONTEMPORARY ECONOMIC POLICY](javascript:;)  Volume: 28   Issue: 3   Pages: 392-405   Published: JUL 2010

# [Toward Understanding the Recent Large Reductions in U.S. Road Fatalities](http://apps.webofknowledge.com.ezproxy.lib.swin.edu.au/full_record.do?product=WOS&search_mode=GeneralSearch&qid=1&SID=C5jzgzYMvSSmhbkgBbF&page=4&doc=191)

By: [Sivak, Michael](http://apps.webofknowledge.com.ezproxy.lib.swin.edu.au/DaisyOneClickSearch.do?product=WOS&search_mode=DaisyOneClickSearch&colName=WOS&SID=C5jzgzYMvSSmhbkgBbF&author_name=Sivak,%20Michael&dais_id=15597&excludeEventConfig=ExcludeIfFromFullRecPage); [Schoettle, Brandon](http://apps.webofknowledge.com.ezproxy.lib.swin.edu.au/DaisyOneClickSearch.do?product=WOS&search_mode=DaisyOneClickSearch&colName=WOS&SID=C5jzgzYMvSSmhbkgBbF&author_name=Schoettle,%20Brandon&dais_id=1758169&excludeEventConfig=ExcludeIfFromFullRecPage)

[TRAFFIC INJURY PREVENTION](javascript:;)  Volume: 11   Issue: 6   Pages: 561-566   Article Number: PII 930468348  Published: 2010

# [Poverty as a determinant of young drivers' fatal crash risks](http://apps.webofknowledge.com.ezproxy.lib.swin.edu.au/full_record.do?product=WOS&search_mode=GeneralSearch&qid=1&SID=C5jzgzYMvSSmhbkgBbF&page=4&doc=200)

By: [Males, Mike A.](http://apps.webofknowledge.com.ezproxy.lib.swin.edu.au/DaisyOneClickSearch.do?product=WOS&search_mode=DaisyOneClickSearch&colName=WOS&SID=C5jzgzYMvSSmhbkgBbF&author_name=Males,%20Mike%20A.&dais_id=1106853&excludeEventConfig=ExcludeIfFromFullRecPage)

[JOURNAL OF SAFETY RESEARCH](javascript:;)  Volume: 40   Issue: 6   Pages: 443-448   Published: 2009

# [Use of Car Crashes Resulting in Fatal and Serious Injuries to Analyze a Safe Road Transport System Model and to Identify System Weaknesses](http://apps.webofknowledge.com.ezproxy.lib.swin.edu.au/full_record.do?product=WOS&search_mode=GeneralSearch&qid=1&SID=C5jzgzYMvSSmhbkgBbF&page=5&doc=202)

[TRAFFIC INJURY PREVENTION](javascript:;)  Volume: 10   Issue: 5   Pages: 441-450   Article Number: PII 914723776  Published: 2009

# [Factors Associated with Fatal Traffic Accidents in Tirana, Albania: Cross-sectional Study](http://apps.webofknowledge.com.ezproxy.lib.swin.edu.au/full_record.do?product=WOS&search_mode=GeneralSearch&qid=1&SID=C5jzgzYMvSSmhbkgBbF&page=5&doc=204)

By: [Qirjako, Gentiana](http://apps.webofknowledge.com.ezproxy.lib.swin.edu.au/DaisyOneClickSearch.do?product=WOS&search_mode=DaisyOneClickSearch&colName=WOS&SID=C5jzgzYMvSSmhbkgBbF&author_name=Qirjako,%20Gentiana&dais_id=3382946&excludeEventConfig=ExcludeIfFromFullRecPage); [Burazeri, Genc](http://apps.webofknowledge.com.ezproxy.lib.swin.edu.au/DaisyOneClickSearch.do?product=WOS&search_mode=DaisyOneClickSearch&colName=WOS&SID=C5jzgzYMvSSmhbkgBbF&author_name=Burazeri,%20Genc&dais_id=458585&excludeEventConfig=ExcludeIfFromFullRecPage); [Hysa, Bajram](http://apps.webofknowledge.com.ezproxy.lib.swin.edu.au/DaisyOneClickSearch.do?product=WOS&search_mode=DaisyOneClickSearch&colName=WOS&SID=C5jzgzYMvSSmhbkgBbF&author_name=Hysa,%20Bajram&dais_id=8005720&excludeEventConfig=ExcludeIfFromFullRecPage); et al.

[CROATIAN MEDICAL JOURNAL](javascript:;)  Volume: 49   Issue: 6   Pages: 734-740   Published: DEC 2008

# [Impact of BAC limit reduction on different population segments: A Poisson fixed effect analysis](http://apps.webofknowledge.com.ezproxy.lib.swin.edu.au/full_record.do?product=WOS&search_mode=GeneralSearch&qid=1&SID=C5jzgzYMvSSmhbkgBbF&page=5&doc=213)

By: [Kaplan, Sigal](http://apps.webofknowledge.com.ezproxy.lib.swin.edu.au/DaisyOneClickSearch.do?product=WOS&search_mode=DaisyOneClickSearch&colName=WOS&SID=C5jzgzYMvSSmhbkgBbF&author_name=Kaplan,%20Sigal&dais_id=579001&excludeEventConfig=ExcludeIfFromFullRecPage); [Prato, Carlo Giacomo](http://apps.webofknowledge.com.ezproxy.lib.swin.edu.au/DaisyOneClickSearch.do?product=WOS&search_mode=DaisyOneClickSearch&colName=WOS&SID=C5jzgzYMvSSmhbkgBbF&author_name=Prato,%20Carlo%20Giacomo&dais_id=480355&excludeEventConfig=ExcludeIfFromFullRecPage)

[ACCIDENT ANALYSIS AND PREVENTION](javascript:;)  Volume: 39   Issue: 6   Pages: 1146-1154   Published: NOV 2007

# [General deterrence effects of US statutory DUI fine and jail penalties: Long-term follow-up in 32 states](http://apps.webofknowledge.com.ezproxy.lib.swin.edu.au/full_record.do?product=WOS&search_mode=GeneralSearch&qid=1&SID=C5jzgzYMvSSmhbkgBbF&page=5&doc=216)

By: [Wagenaar, Alexander C.](http://apps.webofknowledge.com.ezproxy.lib.swin.edu.au/DaisyOneClickSearch.do?product=WOS&search_mode=DaisyOneClickSearch&colName=WOS&SID=C5jzgzYMvSSmhbkgBbF&author_name=Wagenaar,%20Alexander%20C.&dais_id=167754&excludeEventConfig=ExcludeIfFromFullRecPage); [Maldonado-Molina, Mildred M.](http://apps.webofknowledge.com.ezproxy.lib.swin.edu.au/DaisyOneClickSearch.do?product=WOS&search_mode=DaisyOneClickSearch&colName=WOS&SID=C5jzgzYMvSSmhbkgBbF&author_name=Maldonado-Molina,%20Mildred%20M.&dais_id=643654&excludeEventConfig=ExcludeIfFromFullRecPage); [Erickson, Darin J.](http://apps.webofknowledge.com.ezproxy.lib.swin.edu.au/DaisyOneClickSearch.do?product=WOS&search_mode=DaisyOneClickSearch&colName=WOS&SID=C5jzgzYMvSSmhbkgBbF&author_name=Erickson,%20Darin%20J.&dais_id=92578&excludeEventConfig=ExcludeIfFromFullRecPage); et al.

[ACCIDENT ANALYSIS AND PREVENTION](javascript:;)  Volume: 39   Issue: 5   Pages: 982-994   Published: SEP 2007

# [Graduated driver licensing and teen traffic fatalities](http://apps.webofknowledge.com.ezproxy.lib.swin.edu.au/full_record.do?product=WOS&search_mode=GeneralSearch&qid=1&SID=C5jzgzYMvSSmhbkgBbF&page=5&doc=229)

By: [Dee, TS](http://apps.webofknowledge.com.ezproxy.lib.swin.edu.au/DaisyOneClickSearch.do?product=WOS&search_mode=DaisyOneClickSearch&colName=WOS&SID=C5jzgzYMvSSmhbkgBbF&author_name=Dee,%20TS&dais_id=975367&excludeEventConfig=ExcludeIfFromFullRecPage); [Grabowski, DC](http://apps.webofknowledge.com.ezproxy.lib.swin.edu.au/DaisyOneClickSearch.do?product=WOS&search_mode=DaisyOneClickSearch&colName=WOS&SID=C5jzgzYMvSSmhbkgBbF&author_name=Grabowski,%20DC&dais_id=179603&excludeEventConfig=ExcludeIfFromFullRecPage); [Morrisey, MA](http://apps.webofknowledge.com.ezproxy.lib.swin.edu.au/DaisyOneClickSearch.do?product=WOS&search_mode=DaisyOneClickSearch&colName=WOS&SID=C5jzgzYMvSSmhbkgBbF&author_name=Morrisey,%20MA&dais_id=176359&excludeEventConfig=ExcludeIfFromFullRecPage)

[JOURNAL OF HEALTH ECONOMICS](javascript:;)  Volume: 24   Issue: 3   Pages: 571-589   Published: MAY 2005

# [Alcohol, public policy, and highway crashes - A time-series analysis of older-driver safety](http://apps.webofknowledge.com.ezproxy.lib.swin.edu.au/full_record.do?product=WOS&search_mode=GeneralSearch&qid=1&SID=C5jzgzYMvSSmhbkgBbF&page=5&doc=235)

By: [McCarthy, PS](http://apps.webofknowledge.com.ezproxy.lib.swin.edu.au/DaisyOneClickSearch.do?product=WOS&search_mode=DaisyOneClickSearch&colName=WOS&SID=C5jzgzYMvSSmhbkgBbF&author_name=McCarthy,%20PS&dais_id=867692&excludeEventConfig=ExcludeIfFromFullRecPage)

[JOURNAL OF TRANSPORT ECONOMICS AND POLICY](javascript:;)  Volume: 39   Pages: 109-125   Part: 1   Published: JAN 2005

# [THE CONNECTION BETWEEN RISKY DRIVING AND INVOLVEMENT IN FATAL ACCIDENTS](http://apps.webofknowledge.com.ezproxy.lib.swin.edu.au/full_record.do?product=WOS&search_mode=GeneralSearch&qid=1&SID=C5jzgzYMvSSmhbkgBbF&page=6&doc=264)

By: [RAJALIN, S](http://apps.webofknowledge.com.ezproxy.lib.swin.edu.au/DaisyOneClickSearch.do?product=WOS&search_mode=DaisyOneClickSearch&colName=WOS&SID=C5jzgzYMvSSmhbkgBbF&author_name=RAJALIN,%20S&dais_id=4260062&excludeEventConfig=ExcludeIfFromFullRecPage)

[ACCIDENT ANALYSIS AND PREVENTION](javascript:;)  Volume: 26   Issue: 5   Pages: 555-562   Published: OCT 1994

# [Investigation of driver injury severities in rural single-vehicle crashes under rain conditions using mixed logit and latent class models](https://search-informit-com-au.ezproxy.lib.swin.edu.au/documentSummary;res=ATRI;dn=1903AR685E)

Personal Author:[Li, Z](https://search-informit-com-au.ezproxy.lib.swin.edu.au/search;search=pa%3D%22Li,%20Z%22);   [Ci, Y](https://search-informit-com-au.ezproxy.lib.swin.edu.au/search;search=pa%3D%22Ci,%20Y%22);   [Chen, C](https://search-informit-com-au.ezproxy.lib.swin.edu.au/search;search=pa%3D%22Chen,%20C%22);   [Zhang, G](https://search-informit-com-au.ezproxy.lib.swin.edu.au/search;search=pa%3D%22Zhang,%20G%22);   [Wu, Q](https://search-informit-com-au.ezproxy.lib.swin.edu.au/search;search=pa%3D%22Wu,%20Q%22);   [Qian, Z](https://search-informit-com-au.ezproxy.lib.swin.edu.au/search;search=pa%3D%22Qian,%20Z%22);   [Prevedouros, P](https://search-informit-com-au.ezproxy.lib.swin.edu.au/search;search=pa%3D%22Prevedouros,%20P%22);   [Ma, D](https://search-informit-com-au.ezproxy.lib.swin.edu.au/search;search=pa%3D%22Ma,%20D%22)
Source:[PN: Accident Analysis and Prevention, VOL: 124, NO: 0, DATE: 2019-03, PAGES: 219-229, PUBLISHER: Elsevier, ISSN: 0001-4575](https://search-informit-com-au.ezproxy.lib.swin.edu.au/documentSummary;res=ATRI;dn=1903AR685E)

# [Factors affecting the injury severity of out-of-control single-vehicle crashes in Singapore](https://search-informit-com-au.ezproxy.lib.swin.edu.au/documentSummary;res=ATRI;dn=1903AR702E) Personal Author:[Zhou, M](https://search-informit-com-au.ezproxy.lib.swin.edu.au/search;search=pa%3D%22Zhou,%20M%22);   [Chin, H](https://search-informit-com-au.ezproxy.lib.swin.edu.au/search;search=pa%3D%22Chin,%20H%22) Source:[PN: Accident Analysis and Prevention, VOL: 124, NO: 0, DATE: 2019-03, PAGES: 104-112, PUBLISHER: Elsevier, ISSN: 0001-4575](https://search-informit-com-au.ezproxy.lib.swin.edu.au/documentSummary;res=ATRI;dn=1903AR702E)

# [The Bristol Twenty Miles per Hour Limit Evaluation (BRITE) study](https://search-informit-com-au.ezproxy.lib.swin.edu.au/documentSummary;res=ATRI;dn=1803AR106E) Personal Author:[Pilkington, P](https://search-informit-com-au.ezproxy.lib.swin.edu.au/search;search=pa%3D%22Pilkington,%20P%22);   [Bornioli, A](https://search-informit-com-au.ezproxy.lib.swin.edu.au/search;search=pa%3D%22Bornioli,%20A%22);   [Bray, I](https://search-informit-com-au.ezproxy.lib.swin.edu.au/search;search=pa%3D%22Bray,%20I%22);   [Bird, E](https://search-informit-com-au.ezproxy.lib.swin.edu.au/search;search=pa%3D%22Bird,%20E%22) Source:[PN: Project Report, DATE: 2018-02, PAGES: 1 file, PUBLISHER: University of the West of England, T: Bristol, C: United Kingdom](https://search-informit-com-au.ezproxy.lib.swin.edu.au/documentSummary;res=ATRI;dn=1803AR106E)

# [Modeling wrong-way crashes and fatalities on arterials and freeways](https://search-informit-com-au.ezproxy.lib.swin.edu.au/documentSummary;res=ATRI;dn=1807AR206E)

Personal Author:[Ponnaluri, R](https://search-informit-com-au.ezproxy.lib.swin.edu.au/search;search=pa%3D%22Ponnaluri,%20R%22)
Source:[PN: IATSS Research, VOL: 42, NO: 1, DATE: 2018-04, PAGES: 8-17, PUBLISHER: Elsevier, ISSN: 0386-1112](https://search-informit-com-au.ezproxy.lib.swin.edu.au/documentSummary;res=ATRI;dn=1807AR206E)

# [Relationship between heavy vehicle periodic inspections, crash contributing factors and crash severity](https://search-informit-com-au.ezproxy.lib.swin.edu.au/documentSummary;res=ATRI;dn=1809AR106E) Personal Author:[Assemi, B (The University of Queensland)](https://search-informit-com-au.ezproxy.lib.swin.edu.au/search;search=pa%3D%22Assemi,%20B%20(The%20University%20of%20Queensland)%22);   [Hickman, M (The University of Queensland)](https://search-informit-com-au.ezproxy.lib.swin.edu.au/search;search=pa%3D%22Hickman,%20M%20(The%20University%20of%20Queensland)%22) Source:[PN: Transportation Research Part A: Policy and Practice, VOL: 113, DATE: 2018-07, PAGES: 441-459, PUBLISHER: Elsevier, ISSN: 0965-8564](https://search-informit-com-au.ezproxy.lib.swin.edu.au/documentSummary;res=ATRI;dn=1809AR106E)

# [Comparison of older and younger novice driver crash rates: Informing the need for extended Graduated Driver Licensing restrictions](https://search-informit-com-au.ezproxy.lib.swin.edu.au/documentSummary;res=ATRI;dn=1712AR486E) Personal Author:[Curry, A](https://search-informit-com-au.ezproxy.lib.swin.edu.au/search;search=pa%3D%22Curry,%20A%22);   [Metzger, K](https://search-informit-com-au.ezproxy.lib.swin.edu.au/search;search=pa%3D%22Metzger,%20K%22);   [Williams, A](https://search-informit-com-au.ezproxy.lib.swin.edu.au/search;search=pa%3D%22Williams,%20A%22);   [Tefft, B](https://search-informit-com-au.ezproxy.lib.swin.edu.au/search;search=pa%3D%22Tefft,%20B%22) Source:[PN: Accident Analysis and Prevention, VOL: 108, DATE: 2017-11, PAGES: 66- 73, PUBLISHER: Elsevier, ISSN: 0001-4575](https://search-informit-com-au.ezproxy.lib.swin.edu.au/documentSummary;res=ATRI;dn=1712AR486E)

# [Investigating driver injury severity patterns in rollover crashes using support vector machine models](https://search-informit-com-au.ezproxy.lib.swin.edu.au/documentSummary;res=ATRI;dn=1604AR597E) Personal Author:[Chen, C](https://search-informit-com-au.ezproxy.lib.swin.edu.au/search;search=pa%3D%22Chen,%20C%22);   [Zhang, G](https://search-informit-com-au.ezproxy.lib.swin.edu.au/search;search=pa%3D%22Zhang,%20G%22);   [Qian, Z](https://search-informit-com-au.ezproxy.lib.swin.edu.au/search;search=pa%3D%22Qian,%20Z%22);   [Tarefder, R](https://search-informit-com-au.ezproxy.lib.swin.edu.au/search;search=pa%3D%22Tarefder,%20R%22);   [Tian, Z](https://search-informit-com-au.ezproxy.lib.swin.edu.au/search;search=pa%3D%22Tian,%20Z%22) Source:[PN: Accident Analysis and Prevention, VOL: 90, DATE: 2016-05, PAGES: 128- 139, PUBLISHER: Elsevier, ISSN: 0001-4575](https://search-informit-com-au.ezproxy.lib.swin.edu.au/documentSummary;res=ATRI;dn=1604AR597E)

# [Comprehensive Injury Severity Analysis of SUV and Pickup Truck Rollover Crashes: Alabama Case Study](https://search-informit-com-au.ezproxy.lib.swin.edu.au/documentSummary;res=ATRI;dn=1706AR137E)

Personal Author:[Islam, S](https://search-informit-com-au.ezproxy.lib.swin.edu.au/search;search=pa%3D%22Islam,%20S%22);   [Hossain, AB](https://search-informit-com-au.ezproxy.lib.swin.edu.au/search;search=pa%3D%22Hossain,%20AB%22);   [Barnett, TE](https://search-informit-com-au.ezproxy.lib.swin.edu.au/search;search=pa%3D%22Barnett,%20TE%22)
Source:[PN: Transportation Research Record, NO: 2601, DATE: 2016-00, PAGES: 1-9, PUBLISHER: Transportation Research Board (TRB), ISSN: 0361-1981, ISBN: 9780309441407](https://search-informit-com-au.ezproxy.lib.swin.edu.au/documentSummary;res=ATRI;dn=1706AR137E)

# [Factors contributing to hit-and-run crashes in China](https://search-informit-com-au.ezproxy.lib.swin.edu.au/documentSummary;res=ATRI;dn=1406AR469E)

Personal Author:[Zhang, G](https://search-informit-com-au.ezproxy.lib.swin.edu.au/search;search=pa%3D%22Zhang,%20G%22);   [Li, G](https://search-informit-com-au.ezproxy.lib.swin.edu.au/search;search=pa%3D%22Li,%20G%22);   [Cai, T](https://search-informit-com-au.ezproxy.lib.swin.edu.au/search;search=pa%3D%22Cai,%20T%22);   [Bishai, D](https://search-informit-com-au.ezproxy.lib.swin.edu.au/search;search=pa%3D%22Bishai,%20D%22);   [Wu, C](https://search-informit-com-au.ezproxy.lib.swin.edu.au/search;search=pa%3D%22Wu,%20C%22);   [Chan, Z](https://search-informit-com-au.ezproxy.lib.swin.edu.au/search;search=pa%3D%22Chan,%20Z%22)
Source:[PN: Transportation Research Part F: Traffic Psychology and Behaviour, VOL: 23, NO: 0, DATE: 2014-03, PAGES: 113-124, PUBLISHER: Elsevier](https://search-informit-com-au.ezproxy.lib.swin.edu.au/documentSummary;res=ATRI;dn=1406AR469E)

# [Analysis of Crash Severity Based on Vehicle Damage and Occupant Injuries](https://search-informit-com-au.ezproxy.lib.swin.edu.au/documentSummary;res=ATRI;dn=1407AR351E)

Personal Author:[Qin, X](https://search-informit-com-au.ezproxy.lib.swin.edu.au/search;search=pa%3D%22Qin,%20X%22);   [Wang, K](https://search-informit-com-au.ezproxy.lib.swin.edu.au/search;search=pa%3D%22Wang,%20K%22);   [Cutler, CE](https://search-informit-com-au.ezproxy.lib.swin.edu.au/search;search=pa%3D%22Cutler,%20CE%22)
Source:[PN: Transportation Research Record, NO: 2386, DATE: 2013-00, PAGES: 95–102, PUBLISHER: Transportation Research Board (TRB), ISSN: 0361-1981, ISBN: 9780309287036](https://search-informit-com-au.ezproxy.lib.swin.edu.au/documentSummary;res=ATRI;dn=1407AR351E)

# [Analysis of alcohol-impaired young drivers in fatal crashes](https://search-informit-com-au.ezproxy.lib.swin.edu.au/documentSummary;res=ATRI;dn=1202AR251E) Personal Author:[Pickrell, TM](https://search-informit-com-au.ezproxy.lib.swin.edu.au/search;search=pa%3D%22Pickrell,%20TM%22);   [Starnes, M](https://search-informit-com-au.ezproxy.lib.swin.edu.au/search;search=pa%3D%22Starnes,%20M%22) Source:[NO: DOT HS 811 525, DATE: 2011-12, PAGES: 35p, PUBLISHER: United States. National Highway Traffic Safety Administration (NHTSA), T: Washington, S: DC, C: USA](https://search-informit-com-au.ezproxy.lib.swin.edu.au/documentSummary;res=ATRI;dn=1202AR251E)

# [Analysis of fatal run-off-the-road crashes involving overcorrection](https://search-informit-com-au.ezproxy.lib.swin.edu.au/documentSummary;res=ATRI;dn=0908AR015E) Personal Author:[Spainhour, LK](https://search-informit-com-au.ezproxy.lib.swin.edu.au/search;search=pa%3D%22Spainhour,%20LK%22);   [Mishra, A](https://search-informit-com-au.ezproxy.lib.swin.edu.au/search;search=pa%3D%22Mishra,%20A%22) Source:[PN: Transportation Research Record, NO: 2069, DATE: 2008, PAGES: 1-8, PUBLISHER: Transportation Research Board (TRB), T: Washington, S: DC, C: USA, ISSN: 0361-1981, ISBN: 9780309113410](https://search-informit-com-au.ezproxy.lib.swin.edu.au/documentSummary;res=ATRI;dn=0908AR015E)

# [Bus operator types and driver factors in fatal bus crashes: results from the buses involved in fatal accidents survey](https://search-informit-com-au.ezproxy.lib.swin.edu.au/documentSummary;res=ATRI;dn=0909AR129E) Personal Author:[Blower, D](https://search-informit-com-au.ezproxy.lib.swin.edu.au/search;search=pa%3D%22Blower,%20D%22);   [Green, PE](https://search-informit-com-au.ezproxy.lib.swin.edu.au/search;search=pa%3D%22Green,%20PE%22);   [Matteson, A](https://search-informit-com-au.ezproxy.lib.swin.edu.au/search;search=pa%3D%22Matteson,%20A%22) Source:[PN: Report, NO: FMCSA-RRA-09-041, DATE: 2008-06, PAGES: 23p, PUBLISHER: Federal Motor Carrier Safety Administration, T: Washington, S: DC, C: USA](https://search-informit-com-au.ezproxy.lib.swin.edu.au/documentSummary;res=ATRI;dn=0909AR129E)

# [Examination of macrolevel annual safety performance measures for Virginia](https://search-informit-com-au.ezproxy.lib.swin.edu.au/documentSummary;res=ATRI;dn=0910AR836E) Personal Author:[Kweon, Y-J](https://search-informit-com-au.ezproxy.lib.swin.edu.au/search;search=pa%3D%22Kweon,%20Y-J%22) Source:[PN: Transportation Research Record, NO: 2083, DATE: 2008, PAGES: 9-15, PUBLISHER: Transportation Research Board (TRB), T: Washington, S: DC, C: USA, ISSN: 0361-1981, ISBN: 9780309125956](https://search-informit-com-au.ezproxy.lib.swin.edu.au/documentSummary;res=ATRI;dn=0910AR836E)

# [Comparison of severity affecting factors between young and older drivers involved in single vehicle crashes](https://search-informit-com-au.ezproxy.lib.swin.edu.au/documentSummary;res=ATRI;dn=0501AR074E)

Personal Author:[Dissanayake, S](https://search-informit-com-au.ezproxy.lib.swin.edu.au/search;search=pa%3D%22Dissanayake,%20S%22)
Source:PN: IATSS Research, VOL: 28, NO: 2, DATE: 2004, PAGES: 48-54

# [Effectiveness of the anti-drink driving advertising campaign in New Zealand](https://search-informit-com-au.ezproxy.lib.swin.edu.au/documentSummary;res=ATRI;dn=0001AR282E)

Personal Author:[Tay, R (Queensland University of Technology Centre for Accident Research and Road Safety (CARRS-Q))](https://search-informit-com-au.ezproxy.lib.swin.edu.au/search;search=pa%3D%22Tay,%20R%20(Queensland%20University%20of%20Technology%20Centre%20for%20Accident%20Research%20and%20Road%20Safety%20(CARRS-Q))%22)
Source:[PN: Road and Transport Research, VOL: 8, NO: 4, DATE: 1999-12, PAGES: 3- 15, PUBLISHER: ARRB Transport Research Ltd, ISSN: 1037-5783](https://search-informit-com-au.ezproxy.lib.swin.edu.au/documentSummary;res=ATRI;dn=0001AR282E)

# [A comprehensive study of single and multiple truck crashes using violation and crash data](https://www-scopus-com.ezproxy.lib.swin.edu.au/record/display.uri?eid=2-s2.0-85043514005&origin=resultslist&sort=plf-f&src=s&st1=%28predict*+OR+model%29+AND+%28%22serious+crash*%22+OR+%22serious+accident%22+OR+%22serious+collision%22+OR+%22fatal+accident%22+OR+%22fatal+crash%22+OR+%22fatal+collision%22+OR+%22road+deaths%22+OR+%22road+fatal*%22+OR+%22traffic+fatal*%22+OR+%22collision+fatal*%22+OR+%22accident+fatal*%22%29+AND+%28driver%29+&nlo=&nlr=&nls=&sid=34fc91d90b0a1eb0b79c181c6b17236b&sot=b&sdt=b&sl=290&s=TITLE-ABS-KEY%28%28predict*+OR+model%29+AND+%28%22serious+crash*%22+OR+%22serious+accident%22+OR+%22serious+collision%22+OR+%22fatal+accident%22+OR+%22fatal+crash%22+OR+%22fatal+collision%22+OR+%22road+deaths%22+OR+%22road+fatal*%22+OR+%22traffic+fatal*%22+OR+%22collision+fatal*%22+OR+%22accident+fatal*%22%29+AND+%28driver%29+%29+AND+PUBYEAR+%3e+1983&relpos=52&citeCnt=4&searchTerm=)

[Mashhadi, M.M.R.](https://www-scopus-com.ezproxy.lib.swin.edu.au/authid/detail.uri?origin=resultslist&authorId=57201135587&zone=), [Wulff, S.S.](https://www-scopus-com.ezproxy.lib.swin.edu.au/authid/detail.uri?origin=resultslist&authorId=6604078375&zone=), [Ksaibati, K.](https://www-scopus-com.ezproxy.lib.swin.edu.au/authid/detail.uri?origin=resultslist&authorId=6701626167&zone=) 2018. [Open Transportation Journal](https://www-scopus-com.ezproxy.lib.swin.edu.au/sourceid/21100201912?origin=resultslist). 12, pp. 43-56

# [Adolescent antecedents of high-risk driving behavior into young adulthood: Substance use and parental influences](https://www-scopus-com.ezproxy.lib.swin.edu.au/record/display.uri?eid=2-s2.0-0035437884&origin=resultslist&sort=plf-f&src=s&st1=%28predict*+OR+model%29+AND+%28%22serious+crash*%22+OR+%22serious+accident%22+OR+%22serious+collision%22+OR+%22fatal+accident%22+OR+%22fatal+crash%22+OR+%22fatal+collision%22+OR+%22road+deaths%22+OR+%22road+fatal*%22+OR+%22traffic+fatal*%22+OR+%22collision+fatal*%22+OR+%22accident+fatal*%22%29+AND+%28driver%29+&nlo=&nlr=&nls=&sid=34fc91d90b0a1eb0b79c181c6b17236b&sot=b&sdt=b&sl=290&s=TITLE-ABS-KEY%28%28predict*+OR+model%29+AND+%28%22serious+crash*%22+OR+%22serious+accident%22+OR+%22serious+collision%22+OR+%22fatal+accident%22+OR+%22fatal+crash%22+OR+%22fatal+collision%22+OR+%22road+deaths%22+OR+%22road+fatal*%22+OR+%22traffic+fatal*%22+OR+%22collision+fatal*%22+OR+%22accident+fatal*%22%29+AND+%28driver%29+%29+AND+PUBYEAR+%3e+1983&relpos=406&citeCnt=84&searchTerm=)

[Shope, J.T.](https://www-scopus-com.ezproxy.lib.swin.edu.au/authid/detail.uri?origin=resultslist&authorId=7005362795&zone=), [Waller, P.F.](https://www-scopus-com.ezproxy.lib.swin.edu.au/authid/detail.uri?origin=resultslist&authorId=7101913640&zone=), [Raghunathan, T.E.](https://www-scopus-com.ezproxy.lib.swin.edu.au/authid/detail.uri?origin=resultslist&authorId=35421199600&zone=), [Patil, S.M.](https://www-scopus-com.ezproxy.lib.swin.edu.au/authid/detail.uri?origin=resultslist&authorId=12786121500&zone=) 2001. [Accident Analysis and Prevention](https://www-scopus-com.ezproxy.lib.swin.edu.au/sourceid/19532?origin=resultslist), 33(5), pp. 649-658

**Appendix S5: Table of studies, vehicle type, data level and geographic location**

|  | All vehicles | Buses | Heavy trucks | Cars and light trucks |
| --- | --- | --- | --- | --- |
| Population data | USA: 4  North Dakota: 1  Canada: 1  South Australia: 1 | USA: 2 | USA: 4 | USA: 3  Finland: 1 |
| Sample data | Israel: 1  USA: 1 | None | Wyoming: 1 | None |

**Appendix S6: Visual comparison of reviewer quality scores**

Figure 2. Comparison of reviewer scores for overall statistical quality of studies in chronological order.

Note: Studies scored low (0 to 0.333), medium (0.334 to 0.666) or high (0.667 to 1.00), the division of which is indicated by the low and high dotted line in the figure 2.
